# Gouregine, an *α*-Gem-Dimethyltetradehydrocularine Alkaloid, and Other Aporphinoid Alkaloids from the Bark of *Guatteria olivacea* (Annonaceae) and Their In Vitro Cytotoxic Activities

**DOI:** 10.3390/molecules29163834

**Published:** 2024-08-13

**Authors:** Emmanoel V. Costa, José Guilherme C. Freitas, Steve Pereira Manickchand, Morgana de S. Araújo, Valdenizia R. Silva, Luciano de S. Santos, Hector Henrique Ferreira Koolen, Felipe M. A. da Silva, Milena Botelho Pereira Soares, Daniel P. Bezerra

**Affiliations:** 1 Department of Chemistry, Federal University of Amazonas (UFAM), Manaus 69080-900, AM, Brazil; jguilhermefreitas.00@gmail.com (J.G.C.F.); stevemanickchand@gmail.com (S.P.M.); morgana.souza.araujo@gmail.com (M.d.S.A.); 2 Postgraduate Program in Chemistry, Federal University of Amazonas (UFAM), Manaus 69080-900, AM, Brazil; 3 Gonçalo Moniz Institute, Oswaldo Cruz Foundation (IGM-FIOCRUZ/BA), Salvador 40296-710, BA, Brazil; valdeniziar@gmail.com (V.R.S.); luciano.biomed@gmail.com (L.d.S.S.); milena.soares@fiocruz.br (M.B.P.S.); 4 Metabolomics and Mass Spectrometry Research Group, Amazonas State University (UEA), Manaus 690065-130, AM, Brazil; hkoolen@uea.edu.br; 5 Analytical Center-Multidisciplinary Support Center (CA-CAM), Federal University of Amazonas (UFAM), Manaus 69080-900, AM, Brazil; felipemourams@gmail.com; 6 SENAI Institute of Innovation (ISI) in Health Advanced Systems, University Center SENAI/CIMATEC, Salvador 41650-010, BA, Brazil

**Keywords:** *Guatteria olivacea*, 7,7-dimethylaporhine alkaloids, in vitro cytotoxic activity

## Abstract

*Guatteria olivacea* R.E. Fries is an Amazonian species known as ‘envira-bobó’ and ‘envira-fofa’ and is common in the states of Amazonas, Acre, and Pará. Recently, the essential oil from the leaves of this species has shown promising antitumor activity both in vitro and in vivo. The presence of isoquinoline-derived alkaloids, including aporphinoids and tetrahydroprotoberberine alkaloids, has also been previously reported. In our ongoing search for bioactive compounds from Annonaceae Amazonian plants, the bark of *G. olivacea* was investigated via classical chromatography techniques, which revealed nine compounds, eight isoquinoline-derived alkaloids, a rare alkaloid with a *α*-gem-dimethyltetradehydrocularine structure known as gouregine, seven known aporphinoid alkaloids: isopiline, *O*-methylisopiline, melosmine, 9-hydroxyiguattescine, dihydromelosmine, lysicamine, and guattouregidine, and one known pimaradiene diterpene: acanthoic acid. All the isolated compounds were described for the first time in the bark of *G. olivacea,* and their structures were elucidated by extensive analyses of their 1D and 2D NMR spectra in combination with MS data. The NMR data of the alkaloids isopiline, *O*-methylisopiline, melosmine, dihydromelosmine, and guattouregidine were revised due to incomplete data in the literature and some ambiguities. The in vitro cytotoxic activities of the isolated compounds were evaluated against human cancer (HepG2, KG-1a, and HCT116) and noncancerous (MRC-5) cell lines via the Alamar blue assay after 72 h of incubation. Among the compounds evaluated against human cancer cell lines, the most active was the oxoaporphine alkaloid lysicamine, which has strong activity against HCT116 cells, with an IC_50_ value of 6.64 µg/mL (22.79 µmol/L). Melosmine had a moderate effect on HCT116 cells, with an IC_50_ value of 16.77 µg/mL (49.70 µmol/L), whereas acanthoic acid had moderate effects on HepG2 and HCT116 cells, with IC_50_ values of 14.63 µg/mL (48.37 µmol/L) and 21.25 µg/mL (70.25 µmol/L), respectively.

## 1. Introduction

*Guatteria* is one of the oldest genera of the Annonaceae family and is widely distributed in the Neotropical region, covering approximately 177 recognized species [1]. For more than 220 years, more than 425 species of this genus have been described. A subsequent review carried out by Erkens et al. [2] led to a new reclassification with a total of 307 names accepted thus far. However, a significant decrease in its number of species has been observed due to synonymization. For example, a few years ago, 40 names were synonymized into *Guatteria australis* Saint-Hilaire, leading to a large reduction in the species diversity of the genus [3]. More reductions of this type occurred in the publication by Maas et al. [1], with 34 species names synonymized in *Guatteria punctata* (Aubl.) R. A. Howard (bringing the total number of synonyms to 46), including the type species of the genus *Guatteria glauca* Ruiz & Pav. [1].

On the other hand, these synomimizations do not consider the chemical composition of the previously researched species, demonstrating that they are distinct species or even vary within the same species. Several species synonymous with *G. australis* and *G. punctata* have very different chemical compositions, indicating that they are distinct species. When synonymizing, chemophenetic considerations must be considered. Otherwise, identifying species with pharmacological qualities might be a significant challenge [4,5,6,7,8,9,10,11,12].

*Guatteria olivacea* R. E. Fr. (synonym *G. punctata* (Aubl.) R. A. Howard) is a tree of 10–27 m high and 20–32 cm in diameter with thick, greenish bark. Its flowers are rust colored. It is found in non-flooded forests on clayey soil and can be recognized by its leaves, which are black to dark brown when dry, and by the long leaf base. It is popularly known as “envira-bobó”, “envira-fofa”, “envireira”, “embira”, “embira-branca”, “embira-preta”, envira-branca”, and “envira-preta”, with wide occurrence in Brazil, particularly in the Amazon Biome, mainly in the states of Amazonas, Acre and Pará. Its wood is good quality and widely used in heavy and light construction, furniture, household decorative items, toys, boxes, and crates. In Suriname, it is used as an edible fruit, and its leaves are used in baths as herbs [1,13,14].

A previous phytochemical study of the bark of *G. olivacea* described the isolation and identification of several alkaloids derived from the isoquinoline skeleton, including three phenanthrenes: atherosperminine, argentinine, and atherosperminine *N*-oxide; three aporphines: asimilobine, puterine and discoguatine; two oxoaporphines: liriodenine and oxoputerine; and two tetrahydroprotoberberines: coripalmine and discretine [15]. Recently, reported essential oils from the leaves of this species have shown promising in vitro and in vivo antitumor activity against different tumor cell lines, with their main chemical constituents being terpenoids, such as germacrene D (17.65%), 1-*epi*-cubenol (13.21%), caryophyllene oxide (12.03%), spathulenol (11.26%), (*E*)-caryophyllene (7.26%), bicyclogermacrene (5.87%), and δ-elemene (4.95%) [12]. In another study, the essential oil of the aerial parts of *G. olivacea* was reported to have trypanocidal and antibacterial properties, with the main constituents being terpenoid (*E*)-caryophyllene, germacrene D, *cis*-β-guaiene, δ-cadinene, germacrene B, (*E*)-nerolidol, and spathulenol [16].

These results, compared with some species synonymized within *G. punctata,* indicate that *G. olivacea* could not be synonymized; therefore, it is another species. These results confirm the importance of chemical composition in the chemophenetic relationship for reclassification and synonymization of species.

Thus, the aim of the present study was to continue the phytochemical investigation of the bark of *G. olivacea*, aiming to study its chemical composition for the chemophenetic relationships of the species and in search of bioactive compounds with in vitro antitumor properties.

## 2. Results and Discussion

### 2.1. Structural Elucidation of Compounds

**Figure 1:**
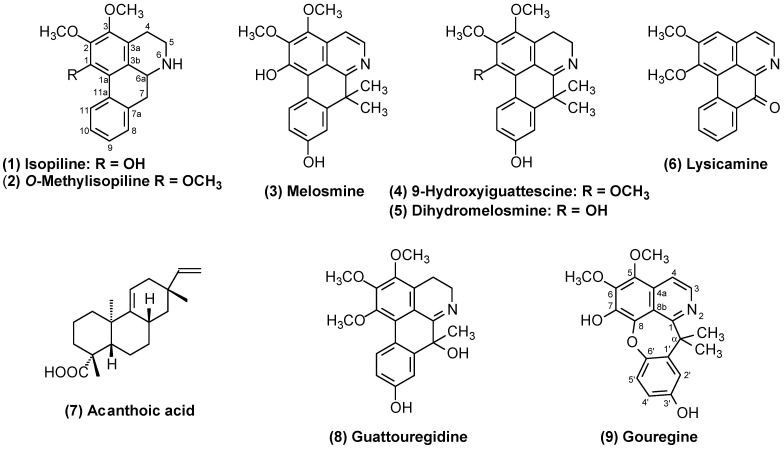
Chemical structures of the isolated compounds from the bark of *G. olivacea*.

After the presence of nitrogen-containing compounds was detected in the methanolic extract according to Dragendorff’s reagent, the extract was subjected to acid–base treatment according to the methodology of Costa et al. [17], resulting in alkaloidal and neutral fractions. A high concentration of nitrogen-containing compounds was observed in the alkaloidal fraction that was subjected to chromatographic investigation. Subsequent classical chromatographic techniques (normal column chromatography—CC, and preparative thin-layer chromatography—PTLC), as described in the Extraction and Isolation section, led to the isolation and identification of nine chemical constituents (**1**–**9**, Figure 1), eight aporphinoid alkaloids, two aporphines (**1** and **2**), three 7,7-dimethylaporphines (**3**, **4** and **5**), one oxoaporhine (**6**), one 7-hydroxy-7-methylaporphine (**8**), one *α*-gem-dimethyltetradehydrocularine (**9**), and one diterpene (**7**). This is the first study of this species in which all its compounds were isolated. The structures of these isolated compounds (Figure 1) were established via extensive analysis via 1D and 2D NMR spectroscopy in combination with MS (Supplementary Materials), as well as comparison with data from previous studies (^1^H and ^13^C NMR datasets).

Compound **9** was obtained as an orange amorphous powder and tested positive for Dragendorff’s reagent. A protonated peak at *m*/*z* 354 [M + H]^+^ in the LR-ESI(+)MS spectrum was compatible with the molecular formula C_20_H_20_NO_5_. The molecular formula C_20_H_20_NO_5_ was confirmed by HRESIMS analysis (*m*/*z* 354.1323 [M + H]^+^, calcd 354.1341). A previous analysis of ^1^H and ^13^C NMR, infrared, and ultraviolet spectra revealed data in agreement with an alkaloid skeleton of the 7,7-dimethylaporphine type [18,19], particularly with the alkaloid melosmine **3** [19,20] (Table 1). On the other hand, a detailed comparative analysis with the data described in this study without ambiguities for the alkaloid melosmine **3** revealed some significant differences, indicating that these compounds are similar but differ in their respective structures (Table 1). A comparison of the molecular formulas of the alkaloids melosmine **3** and compound **9** revealed that the only difference between them is the presence of an additional oxygen in compound **9**, which is clearly involved in the cycle on the basis of the ^1^H and ^13^C NMR spectral data (Table 1), similar to a cularine alkaloid [20]. The presence of hydroxyl groups in the molecule was confirmed by the strong absorption band at 3393 cm^−1^ in the IR spectrum.

In the ^1^H NMR spectrum, eight signals corresponding to the integration of 17 hydrogens were observed, 5 of which were aromatic hydrogens typical of the ABX system at δ_H_ 7.11 (1H, d, *J* = 8.5 Hz, H-5′), δ_H_ 6.95 (1H, d, *J* = 2.9 Hz, H-2′), and δ_H_ 6.71 (1H, dd, *J* = 8.5 and 2.9 Hz, H-4′), and two of which were characteristic of pyridine hydrogens at δ_H_ 8.17 (1H, d, *J* = 2.9 Hz, H-3) and δ_H_ 7.74 (1H, d, *J* = 5.9 Hz, H-4). The other signals correspond to the presence of two methoxy groups at δ_H_ 4.13 (3H, s, H_3_CO-6) and δ_H_ 3.94 (3H, s, H_3_CO-5), and one signal at δ_H_ 1.87 (6H, s, (CH_3_)_2_-α), which are typical of the methyl groups of alkaloids of the 7,7-dimethylapophine type. These signals are very close to the ^1^H NMR signals of melosmine, with some differences in the chemical shifts of some hydrogens, such as the chemical shift of the signals of the methyl groups substituted on the *α*-carbon at δ_H_ 1.87 (6H, s, (CH_3_)_2_-*α*) [19] for compound **9** [20]. (Table 1). These small differences observed between the alkaloids melosmine (**3**) and compound **9** suggest the presence of a cycle dihydrooxepinone, similar to a cularine alkaloid skeleton.

In the ^13^C NMR spectrum, together with the HSQC and HMBC correlation maps, the presence of 20 carbon resonances was verified, 14 of which were aromatic carbons, one imine group (δ_C_ 160.2, C-6a), five aliphatic carbons, two methoxyl groups (δ_C_ 61.6, H_3_CO-5 and δ_C_ 61.2, H_3_CO-6), one quaternary aliphatic carbon (δ_C_ 45.6, C-*α*), and two overlapping methyl groups (δ_C_ 27.5, (CH_3_)_2_-*α*). The presence of oxygenated aromatic carbons of hydroxyl and methoxyl groups can also be confirmed on the basis of the ^13^C NMR signals at δ_C_ 153.8 (C-3′) and δ_C_ 135.8 (C-7), which are in agreement with the IR spectrum. As observed in the ^1^H NMR spectrum, the presence of a probable cularine alkaloid skeleton was confirmed by the shielding of H-5′, C-7, C8a, C-1′, and C-5, and the de-shielding of (CH_3_)_2_-*α*, C-8, C-6′, and C-α (Table 1).

**Figure 2:**
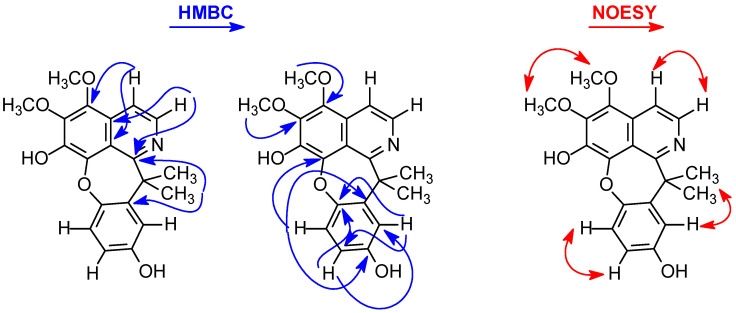
Key HMBC and NOESY correlations for alkaloid **9**.

The location of the methoxyl group in the A ring was established on the basis of the long-range ^1^H-^13^C correlation map from the HMBC NMR experiment (Table 1 and Figure 2). This analysis revealed that the hydrogen at δ_H_ 7.74 (H-4) had a long-range ^1^H-^13^C correlation with *^3^J,* with the carbon at δ_C_ 116.9 (C-8a) and δ_C_ 142.2 (C-5), and with *^2^J,* with the carbon at δ_C_ 127.2 (C-4a) and δ_C_ 137.9 (C-3), confirming that one of the methoxy groups was substituted at C-3 of the A ring. The same analysis was performed for the presence of one of the hydroxyl groups located in the C ring. The signal of the hydrogen at δ_H_ 7.11 (H-5′) showed long-range ^1^H-^13^C correlation to *^3^J* with the signals of the carbons at δ_C_ 140.0 (C-8), δ_C_ 140.2 (C-1′), and δ_C_ 153.9 (C-3′), and to *^2^J* with the signals of the carbons at δ_C_ 149.9 (C-6′) and δ_C_ 114.8 (C-4′). The signal of the hydrogen at δ_H_ 6.95 (H-2′) showed a long-range ^1^H-^13^C correlation to *^3^J* with the signals of the carbons at δ_C_ 114.8 (C-4′) and δ_C_ 149.9 (C-6′), and to *^2^J* with the signals of the carbons at δ_C_ 140.2 (C-1′) and δ_C_ 153.9 (C-3′). The resonate carbon at δ_C_ 153.9 (C-9) did not correlate with any of the methoxy group signals, confirming the presence of one of the hydroxyl groups in the C ring (Figure 2). Thus, the locations of the other hydroxyl and methoxyl groups on the A ring were established on the basis of the NOESY data (Figure 2). In this experiment, the signal of the hydrogen at δ_H_ 7.11 (H-5′) was correlated only with the signal at δ_H_ 6.71 (H-4′), indicating the presence of a hydroxyl group at the C-7 position of ring A. Consequently, the second methoxy group was established at the C-6 (δ_H_ 4.13) position of the A ring due to the correlation in the NOESY experiment (Figure 2) of the signal at δ_H_ 7.74 (H-4), with signals at δ_H_ 3.94 (H_3_CO-5) and δ_H_ 8.17 (H-3). The presence of a methoxyl substituent at C-2 was also confirmed on the basis of the NOESY data by multiple correlations of the signals at δ_H_ 4.13 (H_3_CO-6) with those at δ_H_ 3.94 (H_3_CO-5). Therefore, on the basis of MS, 1D/2D NMR, and IR data and comparisons with the literature data, compound **9** was determined to be a cularine-type alkaloid known as gouregine. This is a rare alkaloid found in the literature with records to date showing it to be in the Annonaceae family, being the second reported alkaloid in the family and described in the literature for the first time in the species *Guatteria ouregou* (Aubl.) Dunal. [20,21].

**Figure 3:**
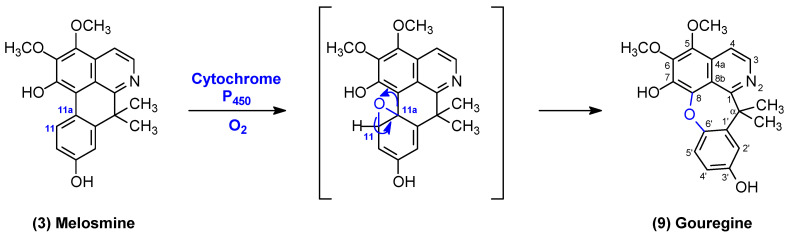
Proposal for the rearrangement of melosmine to generate gouregine. Adapted from Leboeuf et al. [20].

According to Leboeuf et al. [20], the biosynthesis of gouregine apparently does not come from the classical pattern of cularins, that is, from the intramolecular oxidative coupling of an 8-hydroxybenzylisoquinoline. These authors suggested that gouregine is likely biosynthesized from its major constituent, melosmine, through a rearrangement of its skeleton with consecutive oxidation of the double bond between C-11 and C-11a, generating an arene oxide that is rearranged to gouregine. This explanation can be confirmed by treating melosmine with Fenton’s reagent (hydroxyl radicals generated by the decomposition of hydrogen peroxide with ferrous sulfate), which led to the formation of gouregine (Figure 3) in a 90% yield [20]. This oxidation between C-11 and C-11a of the aromatic D-ring of melosmine, which transforms it into an arene oxide (Figure 3), can be easily carried out by the enzyme cytochrome P_450_ [22].

**Figure 4:**
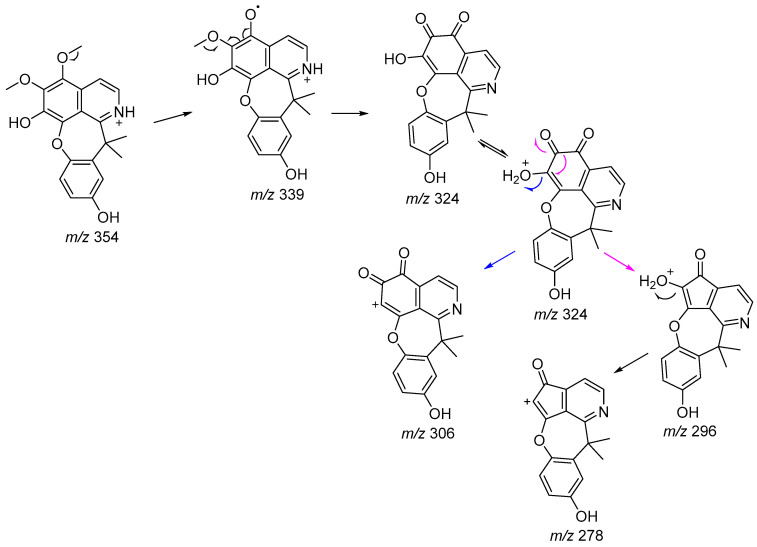
Proposed fragmentation pathway for the major product ions observed in the MS/MS spectrum of the protonated molecule at *m*/*z* 354.

The MS/MS spectrum of protonated molecule **9** at *m*/*z* 354 revealed a fragmentation pathway (Figure 4) consistent with that of isoquinoline-derived alkaloids, which possess methoxyl and hydroxyl groups as substituents in the A ring and iminium nitrogen in the B ring [23]. Thus, two sequential methyl losses (−15 Da) (*m*/*z* 354 → 339 and *m*/*z* 339 → 324) were observed, followed by the competitive loss of water −18 Da (*m*/*z* 324 → 306), which in this case occurred via gas-phase proton transfer [23] and carbon monoxide (−28 Da) (*m*/*z* 324 → 296). These observations agree with the structure of **9**. These results corroborate the ^1^H and ^13^C 1D/2D NMR data, as well as the information from the IR spectrum, confirming the structure of the new alkaloid of the 7,7-dimethylaporphin type.

Compounds **1**–**8** were identified as isopiline (**1**) [19,24], *O*-methylisopiline (**2**) [24], melosmine **3** [19], 9-hydroxyiguattescine **4** [18], dihydromelosmine **5** [19], lysicamine **6** [25], acanthoic acid **7** [26], and guattouregidine **8** [19] on the basis of their spectroscopic profiles and comparison with values in the literature. Although the NMR data of isopiline, *O*-methylisopiline, melosmine, dihydromelosmine, and guattouregidine have already been described in the literature, they are incomplete and contain some ambiguities. Thus, the complete and unequivocal NMR data for these alkaloids were reviewed according to their 1D and 2D NMR data (Table 1 and Table 2). The ^1^H and ^13^C 1D and 2D-NMR spectra, as well as the mass spectra of all the isolated compounds, are available in the Supplementary Materials. All the isolated compounds from this species are described for the first time and contribute significantly to the chemophenetic knowledge of the species, as well as the genus and family.

From a chemophenetic (a new term for plant chemosystematics/plant chemotaxonomy) point of view, the results obtained in this study differ from the results obtained by Araújo et al. [15], who also investigated the bark of another specimen of *G. olivacea* collected in the same collection area as the investigated species in this study, highlighting phenanthrene alkaloids as the main constituents and chemophenetic markers. In this study, the main chemical constituents and chemophenetic markers were 7,7-dimethylaporphine alkaloids, indicating that even between the same species, there is a difference in chemical composition and it can be considered a chemotype. Notably, Maas et al. [1] recently synonymized 34 species names of *G. punctata* (bringing the total number of synonyms to 46), including the type of species of the genus *G. glauca* [1]. Several of these synonymized species have different chemical compositions and are classified as distinct species rather than synonymized species. On the basis of this study and data on the chemical composition of synonymized *Guatteria* species described in the literature, there is a need to include the chemical composition of *Guatteria* species in molecular phylogenetic studies when a given species is synonymized. In this case, *G. olivacea* could not be synonymized, nor could other species of *Guatteria* synonymized by Maas et al. [1].

According to the literature, alkaloids of the 7,7-dimethylaporphine and/or 7-hydroxy-7-methylaporphine types are clearly rare in Annonaceae, being found practically in species of the genus *Guatteria*, with a certain selectivity between species that make up the genus. To date, the presence of 7,7-dimethylaporphine and/or 7-hydroxy-7-methylaporphine alkaloids has been reported in eight of the 177 recognized species, such as *G. ouregou* [19,21], *Guatteria melosma* Diels [27], *Guatteria scandens* Diels [28], *Guatteria discolor* R.E. Fries [29,30], *Guatteria schomburgkiana* Mart. [31], *Guatteria foliosa* Benth. [32], *Guatteria multivenia* Diels [33], and *Guatteria friesiana* [18,34]. Among these species, the most representative in terms of the presence of 7,7-dimethylaporphine and/or 7-hydroxy-7-methylaporphine alkaloids are *G. ouregou*, *G. melosma*, and *G. friesiana*, the major chemical constituents of which have been isolated and identified.

Among the 7,7-dimethylaporphine and/or 7-hydroxy-7-methylaporphine alkaloids found in the *Guatteria* species described above, the following occurrences have been reported: melosmine (**3**) was described in the stem bark of *G. melosma* [27], *G. discolor* [29], and *G. ouregou* [21], and now also in the stem bark of *G. olivacea*; 9-hydroxyiguattescine (**4**) is the second record in the literature, being described for the first time in the stem bark of *G. friesiana* [18]; dihydromelosmine (**5**) was an original synthetic product [35] and was found as a natural product in the stem bark of *G. ouregou* [21], being the second report in the literature; guattouregidine (**8**) was also found in the stem bark of *G. ouregou* [21] and was the second report in the literature; and finally, the gouregine alkaloid, a rare cularine-type alkaloid that has a pattern similar to that of the 7,7-dimethylaporphine-type alkaloids, has been recorded only in the species *G. ouregou* and now in *G. olivacea*.

It is also important to highlight the similarity in terms of the isolated and identified alkaloids between the species *G. ouregou* and *G. olivacea*, which have different morphological characteristics, with emphasis on the alkaloid containing the cularine skeleton named gouregine (Figure 1), which has been found only in these two species to date. These observations further reinforce the importance of the chemical composition of species in phylogenetic studies with the aim of contributing to the correct classification of species within a given genus, as well as synonymy and even variation or chemotyping within the same species, which is observed for the studies described for *G. olivacea*.

### 2.2. Cytotoxicity Assay

The in vitro cytotoxic activities of the isolated compounds **1**–**9** were evaluated against the cancer cell lines HepG2 (human hepatocellular carcinoma), KG-1a (human myeloid leukemia), and HCT116 (human colon carcinoma), and the noncancerous cell line MRC-5 (human lung fibroblast) via the Alamar blue assay after 72 h of incubation.

Among the compounds evaluated (Table 3), the most promising result was verified for the oxoaporphine alkaloid lysicamine (**6**), which has strong activity against HCT116 cells, with an IC_50_ value of 6.64 μg/mL (22.79 µmol/L) and a selectivity index of 2.60 (Table S1). These results agree with the results of De Souza et al. [36], who reported the activity of this alkaloid against HL-60 and K562 leukemia cells, with IC_50_ values of 7.11 μg/mL (24.40 µmol/L) and 11.29 μg/mL (38.75 µmol/L), respectively. Similarly, Omar et al. [37] reported that lysicamine has IC_50_ values of 26 μg/mL (89.24 µmol/L) for MCF-7 breast cancer cells and 27 μg/mL (92.67 µmol/L) for HepG2 cells. Interestingly, it has been reported that lysicamine can inhibit Akt activation in anaplastic human thyroid cancer cells [38] and suppress human colon cancer cell lines and cancer stem cells through the inhibition of Wnt/β-catenin [39]. Similarly, liriodenine caused apoptosis in CAOV-3 ovarian cancer cells via the mitochondrial pathway via the activation of caspase-3 and caspase-9 [40]. The alkaloid melosmine (**3**) showed moderate activity against HCT116, with an IC_50_ value of 16.77 μg/mL (49.70 µmol/L). Previously, melosmine was reported to be an antimalarial agent with low cytotoxicity to human cancer cells [41]. The same moderate activity was observed for the pimaradiene diterpene acanthoic acid against HepG2 and HCT116 cells, with IC_50_ values of 14.63 μg/mL (48.37 µmol/L) and 21.25 μg/mL (70.25 µmol/L), respectively. On the other hand, it should be noted that acanthoic acid (**7**), among the active compounds, did not show cytotoxic activity against noncancerous MRC-5 cells up to the evaluated concentration of 25 μg/mL (82.65 µmol/L). Importantly, acanthoic acid has been reported to have anti-inflammatory activity with low cytotoxicity to cancer cells [42,43,44,45,46]. Furthermore, Kim et al. [47] demonstrated that acanthoic acid increased the levels of cleaved caspase 3 and cleaved PARP1 and reduced the levels of the antiapoptotic protein Bcl-xL, causing apoptotic cell death via activation of the p38 MAPK pathway in HL-60 leukemia cells. The other compounds did not show cytotoxic activity against either cancer cells or noncancerous cells, which is still a good result considering the results of other biological assays, such as those evaluating antimicrobial, antiparasitic, and antiviral activities.

## 3. Materials and Methods

### 3.1. General Experimental Procedures

Fourier transform infrared (FTIR) spectra were obtained on a Thermo Scientific Nicolet iS5 spectrometer (Thermo Fisher Scientific Inc., Waltham, MA, USA) coupled to an ATR iD3 instrument with ZnSe crystals. The 1D and 2D NMR data were acquired in CDCl_3_ (chloroform-*d*) at 298 K on an AVANCE III HD NMR spectrometer (Bruker, Billerica, MA, USA) operating at 11.75 T (^1^H and ^13^C at 500 and 125 MHz, respectively). All the ^1^H- and ^13^C-NMR chemical shifts (δ) are presented in ppm relative to the tetramethylsilane signal at 0.00 ppm as an internal reference, and the coupling constants (*J*) are given in Hz. The NMR spectrometer was equipped with a 5 mm multinuclear inverse detection probe (for 1D and 2D NMR experiments) with a z gradient. One-bond (HSQC) and two- and three-bond (HMBC) ^1^H-^13^C-NMR correlation experiments were optimized for average coupling constants ^1^*J*_(C,H)_ and ^LR^*J*_(C,H)_ of 140 and 8 Hz, respectively. For low-resolution mass spectrometry (LR-MS) analysis, the samples of the isolated compounds were resuspended in methanol (HPLC grade), creating stock solutions (1 mg/mL). Aliquots (5 µL) of the stock solutions were further diluted to 5 µg/mL and analyzed by direct infusion into a triple-quadrupole mass spectrometer, model TSQ Quantum Access (Thermo Scientific, San Jose, CA, USA), equipped with electrospray ionization (ESI) or atmospheric-pressure chemical ionization (APCI) sources in negative or positive mode. An HPLC Shimadzu (Kyoto, Japan) coupled with a MicroTOF II (Bruker Daltonics, Billerica, MA, USA) with an electrospray ionization (ESI) source was used to obtain high-resolution mass spectra (HRESIMS) in positive mode. The parameters were as follows: capillary voltage of 4.5 kV, ESI in positive mode, final plate offset of 500 V, 40 psi nebulizer, dry gas (N_2_) flow rate of 8 mL/min, and temperature of 200 °C. The mass spectra (*m*/*z* 50–1000) were recorded every 2 s. Silica gel 60 (Sigma-Aldrich, San Luis, MO, USA, 70–230 mesh) was used for column chromatography (CC), whereas silica gel 60 F254 (Macherey-Nagel, Düren, Germany, 0.25 mm, aluminum) was used for analysis and preparation with thin-layer chromatography (PTLC) (Macherey-Nagel, 1.00 mm, glass). The compounds were visualized by exposure to UV_254/365_ light, spraying with *p*-anisaldehyde reagent, heating on a hot plate, and spraying with Dragendorff’s reagent.

### 3.2. Plant Material

In the present investigation, the botanical material (bark) of *G. olivacea* was collected on 16 December 2021, at the Adolpho Ducke Reserve (geographic coordinates: 2°54′47″ S and 59°58′48″ W), Manaus, Amazonas State, Brazil, and identified by Prof. Dr. Antonio Carlos Webber, a plant taxonomist of the Department of Biology of the Federal University of Amazonas (DB/UFAM). The voucher specimen number 11423 was deposited at the Herbarium of DB/UFAM. The accession (specimen) was registered in the ‘Sistema Nacional de Gestão do Patrimônio Genético e do Conhecimento Tradicional Associado (SISGEN)’ with the record A70EDCD.

### 3.3. Extraction and Isolation

The bark of *G. olivacea* was dried in an air-circulating oven at approximately 45 °C for 72 h and subsequently pulverized in a four-knife mill grinder (Marconi) to obtain the powdered material (1263.37 g). Then, exhaustive maceration with hexane (5 × 4.5 L, 25 °C) followed by MeOH (5 × 4.5 L, 25 °C) was performed. The extractive solutions obtained were concentrated in a rotary evaporator at reduced pressure (40–50 °C) to obtain hexane (19.45 g) and MeOH (107.17 g) extracts.

TLC analysis with Dragendorff’s reagent revealed a high presence of alkaloids in the MeOH extract. Therefore, an aliquot of MeOH extract (105.0 g) was initially subjected to acid–base extraction [12] to obtain alkaloidal (2.36 g) and neutral (13.07 g) fractions. Subsequently, part of the alkaloidal fraction (2.20 g) was subjected to silica-gel chromatographic column (CC) treatment with a 10% NaHCO_3_ solution [12] and eluted with hexane (100%), hexane–CH_2_Cl_2_ (90:10, 80:20, 70:30, 60:40, 50:50, 40:60, 30:70, 20:80, and 10:90, *v*/*v*), CH_2_Cl_2_ (100%), CH_2_Cl_2_–EtOAc (90:10, 80:20, 70:30, 60:40, 50:50, 40:60, 30:70, 20:80, and 10:90, *v*/*v*), EtOAc (100%), EtOAc–MeOH (95:05, 90:10, 85:05, 80:10, 75:25, 70:30, 60:40, and 50:50), and finally, MeOH. For each eluent, 200 mL of the mobile phase was used and collected in 30 mL vials during chromatographic elution, yielding 213 fractions at the end of the chromatographic separation process. After TLC evaluation using a mixture of CH_2_Cl_2_–MeOH at proportions of 95:05, 90:10, 85:15, and 80:20 as the eluent system (*v*/*v*), similar samples were pooled to yield 17 fractions (F1 to F17).

Fraction F5 (301.6 mg) from CC eluted with hexane–CH_2_Cl_2_ (10:90, *v*/*v*), CH_2_Cl_2_ (100%), and CH_2_Cl_2_–EtOAc (90:10, *v*/*v*) was subjected to preparative TLC and eluted with CH_2_Cl_2_–MeOH (95:05, *v*/*v*, three elutions), affording **1** (5.7 mg), **2** (8.6 mg), and **3** (57.7 mg), respectively. Fraction F6 (84.3 mg) from CC eluted with CH_2_Cl_2_–EtOAc (90:10, 80:20, and 70:30 *v*/*v*) was also subjected to preparative TLC and eluted with CH_2_Cl_2_–MeOH (95:05, *v*/*v*, two elutions), yielding **3** (45.0 mg) and **4** (5.7 mg) again.

Fractions F7 (26.3 mg) and F8 (14.0 mg), both from CC eluted with CH_2_Cl_2_–EtOAc (70:30 and 60:40 *v*/*v*), were pooled and subjected to preparative TLC eluted with CH_2_Cl_2_–MeOH (95:05, *v*/*v*, three elutions), yielding **3** (11.8 mg), **4** (9.6 mg), **5** (5.1 mg), and **6** (2.8 mg), respectively.

Fraction F9 (94.7 mg) from CC eluted with CH_2_Cl_2_–EtOAc (60:40, 50:50, 40:60, 30:70, 20:80 and 10:90 *v*/*v*), EtOAc (100%), and EtOAc–CH_3_OH (90:10) was subjected to preparative TLC eluted with CH_2_Cl_2_–MeOH (95:05, *v*/*v*, three elutions), affording **3** (16.6 mg) and **5** (11.4 mg), respectively. Fraction 10 (64.0 mg) from CC eluted with CH_2_Cl_2_–EtOAc (90:10 *v*/*v*) was also subjected to the same methodology described above, affording **3** (5.0 mg) and **7** (3.0 mg) again.

Fraction F11 (206.1 mg) from CC eluted with CH_2_Cl_2_–EtOAc (90:10 and 80:20, *v*/*v*) was subjected to preparative TLC and eluted with CH_2_Cl_2_–MeOH (95:05, *v*/*v*, three elutions), yielding **3** (5.0 mg), **8** (2.4 mg), and **9** (3.4 mg). Both Fraction 12 (71.6 mg) from CC eluted with CH_2_Cl_2_–EtOAc (80:20 and 70:30, *v*/*v*) and Fraction 13 (86.0 mg) from CC eluted with CH_2_Cl_2_–EtOAc (70:30 and 60:34, *v*/*v*) were also subjected to the same methodology described above, yielding **9** (38.0 mg).

As observed in the isolation and purification methodology of the compounds, compound **3** was present in practically all the fractions studied, indicating that the plant biosynthesizes this compound in large quantities and is the major compound.

*Isopiline* (**1**): Brown amorphous powder; ^1^H-NMR and ^13^C-NMR data were reviewed on the basis of 1D and 2D-NMR experiments and are described in Table 2. LR-APCI(+)-MS [M + H]^+^ *m*/*z* 298.

*O-Methylisopiline* (**2**): Brown amorphous powder; ^1^H-NMR and ^13^C-NMR data were reviewed on the basis of 1D and 2D-NMR experiments and are described in Table 2. LR-APCI(+)-MS [M + H]^+^ *m*/*z* 312.

*Melosmine* (**3**): Orange amorphous powder; ^1^H-NMR and ^13^C-NMR data were reviewed on the basis of 1D and 2D-NMR experiments and are described in Table 2. LR-APCI(+)-MS [M + H]^+^ *m*/*z* 338.

*9-Hydroxyguattescine* (**4**): Orange–yellow amorphous powder; ^1^H-NMR and ^13^C-NMR in accordance with the literature [18]. LR-APCI(+)-MS [M + H]^+^ *m*/*z* 354.

*Dihydromelosmine* (**5**): Brown amorphous powder; ^1^H-NMR and ^13^C-NMR data were reviewed on the basis of 1D and 2D-NMR experiments and are described in Table 1. LR-APCI(+)-MS [M + H]^+^ *m*/*z* 340.

*Lysicamine* (**6**): Yellow crystals (CH_2_Cl_2_-MeOH 3:1); ^1^H-NMR and ^13^C-NMR in accordance with previously reported methods [25]. LR-APCI(+)-MS [M + H]^+^ *m*/*z* 292.

*Acanthoic acid* (**7**): White needles (hexane CH_2_Cl_2_ 3:1); ^1^H-NMR and ^13^C-NMR in accordance with previously reported methods [26]. LR-APCI(−)-MS [M − H]^−^ *m*/*z* 301.

*Guattouregidine* (**8**): Brown amorphous powder; ^1^H-NMR and ^13^C-NMR data were reviewed on the basis of 1D and 2D-NMR experiments and are described in Table 1. LR-APCI(+)-MS [M + H]^+^ *m*/*z* 342.

*Gouregine* (**9**): Orange amorphous powder; ^1^H-NMR and ^13^C-NMR data are described in Table 1. LR-ESI(+)-MS [M + H]^+^ *m*/*z* 354. HRESIMS(+) 354.1323 [M + H]^+^.

#### 3.3.1. Cells

The HepG2 (human hepatocellular carcinoma), KG-1a (human myeloid leukemia), HCT116 (human colon carcinoma), and MRC-5 (human lung fibroblast) cell lines were obtained from the American Type Culture Collection (ATCC, Manassas, VA, USA) and were cultured as recommended by the American Type Culture Collection (ATCC) animal culture guide [48]. All the cell lines were tested for mycoplasma via a mycoplasma stain kit (Sigma-Aldrich) to validate the use of cells that were free from contamination.

#### 3.3.2. Cytotoxicity Assay

For the cytotoxicity assay, cell viability was quantified via the Alamar blue method, as previously described [49]. For all the experiments, the cells were plated in 96-well plates. The chemical constituents evaluated varied in purity between 90% and 99.8%, which was determined on the basis of the relative integrals of the signals of the constituents in relation to the signals of the impurities contained. Most of the constituents had purities greater than 95%, except for constituents **4** and **5**, which had purities of approximately 90%. The chemical constituents were dissolved in dimethyl sulfoxide (DMSO, Vetec Química Fina Ltda., Duque de Caxias, RJ, Brazil), added to each well, and incubated for 72 h. Doxorubicin (doxorubicin hydrochloride, purity ≥95%, Laboratory IMA S.A.I.C., Buenos Aires, Argentina) was used as a positive control. At the end of the treatment, 20 µL of a stock solution (0.312 mg/mL) of resazurin (Sigma-Aldrich Co.) was added to each well. The absorbances at 570 nm and 600 nm were measured via a SpectraMax 190 Microplate Reader (Molecular Devices, Sunnyvale, CA, USA). The half-inhibitory concentration (IC_50_) was obtained via nonlinear regression with 95% confidence intervals (CIs 95%) via the software GraphPad Prism 8 (Intuitive Software for Science; San Diego, CA, USA).

## 4. Conclusions

The phytochemical investigation of the bark of *G. olivacea* led to the isolation and identification of nine compounds (**1**–**9**); eight isoquinoline-derived alkaloids, including a rare alkaloid with a *α*-*gem*-dimethyltetradehydrocularine structure known as gouregine (**9**); seven known aporphinoid alkaloids: isopiline (**1**), *O*-methylisopiline (**2**), melosmine (**3**), 9-hydroxyiguattescine (**4**), dihydromelosmine (**5**), lysicamine (**6**), and guattouregidine (**8**); and one known pimaradiene diterpene: acanthoic acid (**7**). These isolated compounds were described for the first time in the bark of *G. olivacea*. Most of the constituents identified were the alkaloids 7,7-dimethylaporphines (melosmine, 9-hydroxyiguattescine, and dihydromelosmine) and/or 7-hydroxy-7-methylaporphine (guattouregidine), with an emphasis on the alkaloid melosmine.

These results contribute significantly to the chemophenetic knowledge of the species, considering that the species *G. olivacea* was synonymized as *G. punctata*, which currently comprises approximately 46 synonyms. This result reinforces the importance of chemical composition today in the reclassification of a given species and must be taken into consideration, not only with molecular phylogenetic data.

The in vitro cytotoxic activities of the isolated compounds were evaluated against cancer (HepG2, KG-1a, and HCT116) and noncancerous (MRC-5) cell lines via the Alamar blue assay after 72 h of incubation. Among the compounds evaluated against human cancer cell lines, the most active was the oxoaporphine alkaloid lysicamine, which has strong activity against HCT116 cells, with an IC_50_ value of 6.64 µg/mL (22.79 µmol/L). Melosmine had a moderate effect on HCT116 cells, with an IC_50_ value of 16.77 µg/mL (49.70 µmol/L), whereas acanthoic acid had moderate effects on HepG2 and HCT116 cells, with IC_50_ values of 14.63 µg/mL (48.37 µmol/L) and 21.25 µg/mL (70.25 µmol/L), respectively.

The results obtained in this study indicate that *G. olivacea* is a typical Annonaceae species belonging to a genus (*Guatteria*) where selective species biosynthesize alkaloids of the 7,7-dimethylaporphine and 7-hydroxy-7-methylaporphine types and deserves further investigation of other compounds belonging to these classes with cytotoxic activities.

## Figures and Tables

**Table 1:** ^1^H (500 MHz) and ^13^C NMR (125 MHz) data for alkaloids **3**, **5**, **8,** and **9**.

Position	3		5		8		Position	9	
	*δ*_C_ mult.	*δ*_H_ mult. (*J* in Hz)	*δ*_C_ mult.	*δ*_H_ mult. (*J* in Hz)	*δ*_C_ mult.	*δ*_H_ mult. (*J* in Hz)		*δ*_C_ mult.	*δ*_H_ mult. (*J* in Hz)
**1**	144.4, qC		144.5, qC		144.0, qC		1	160.2, qC	
1a	111.3, qC		116.7, qC		116.0, qC		3	137.9, CH	8.17 *d* (5.9)
2	142.2, qC		142.6, qC		142.9, qC		4	114.2, CH	7.74 *d* (5.9)
3	142.8, qC		146.7, qC		147.0, qC		4a	127.2, qC	
3a	127.2, qC		122.8, qC		122.4, qC		5	142.2, qC	
3b	119.4, qC		119.1, qC		117.4, qC		6	142.7, qC	
4ax 4eq	112.3, CH	7.68 *d* (5.6)	19.4, CH_2_	2.61 *t* (7.2)	19.5, CH_2_	2.42 *td* (15.3; 5.5) 2.94 *dddd* (16.5; 15.3; 4.7; 0.8)	7	135.8, qC	
5ax 5eq	140.8, CH	8.46 *d* (5.6)	46.0, CH_2_	3.62 *t* (7.2)	45.6, CH_2_	3.20 *dddd* (16.5; 15.3; 5.5; 0.8) 4.09 *dd* (15.3; 5.5)	8	140.0, qC	
6a	163.0, qC		173.0 qC		170.7, qC		8a	116.9, qC	
7	42.1 qC		42.9, qC		72.7, qC		1′	140.2, qC	
7a	147.0, qC		145.1, qC		144.1, qC		2′	113.8, CH	6.95 *d* (2.9)
8	113.4, CH	7.17 *d* (2.7)	111.8, CH	7.01 *d* (2.6)	111.5, CH	7.30 *d* (2.7)	3′	153.9, qC	
9	155.5, qC		156.0, qC		155.5, qC		4′	114.8, CH	6.71 *dd* (8.5; 2.9)
10	113.7, CH	6.86 *dd* (8.8; 2.7)	113.4, CH	6.78 *dd* (8.7; 2.6)	127.5, CH	6.80 *dd* (8.7; 2.7)	5′	122.3, CH	7.11 *d* (8.5)
11	129.0, CH	8.87 *d* (8.8)	129.8, CH	8.45 *d* (8.7)	129.6, CH	8.44 *d* (8.7)	6′	149.9, qC	
11a	121.7, qC		121.9, qC		120.9, qC		α	45,6, qC	
1-OH							α-(CH_3_)_2_	27.5, CH_3_	1.87 *s*
2-OCH_3_	61.2, CH_3_	4.17 *s*	61.0	4.04 *s*	61.1	4.04 *s*	5-OCH_3_	61.6, CH_3_	3.94 *s*
3-OCH_3_	61.1, CH_3_	3.99 *s*	60.6	3.86 *s*	60.7	3.87 *s*	6-OCH_3_	61.2, CH_3_	4.13 *s*
9-OH							7-OH		
7-(CH_3_)_2_	32.6, CH_3_	1.73 *s*	27.3	1.47 *s*	33.3	1.47 *s*	3′-OH		
7-OH									

The data were obtained at 298 K with TMS as an internal reference (0.00 ppm) in CDCl_3_.

**Table 2:** ^1^H (500 MHz) and ^13^C NMR (125 MHz) data for alkaloids **1** and **2**.

Position	1		2	
	*δ*_C_ mult.	*δ*_H_ mult. (*J* in Hz)	*δ*_C_ mult.	*δ*_H_ mult. (*J* in Hz)
1	145.5, qC		150.3, qC	
1a	115.4, qC		122.3, qC	
2	138.9, qC		145.6, qC	
3	148.9, qC		150.6, qC	
3a	130.1, qC		131.0, qC	
3b	117.7, qC		122.4, qC	
4ax 4eq	22.4, CH_2_	2.84 *m*	23.2, CH_2_	2.84 *m*
5ax 5eq	42.2, CH_2_	2.99 *m* 3.54 *m*	42.5, CH_2_	2.97 *m* 3.48 *ddd* (12.2; 5.0; 1.9)
6a	53.5, CH	3.97 *m*	53.7, CH	3.87 *d* (13.8; 4.7)
7ax 7eq	36.0, CH_2_	2.88 t (13.8) 2.99 dd (13.8; 4.5)	36.5, CH_2_	2.82 *t* (13.8) 2.92 *dd* (13.8; 4.7)
7a	134.3, qC		135.0, qC	
8	127.8, CH	7.21 *br d* (7.2)	127.83, CH	7.23 *dd* (7.3; 1.7)
9	126.8, CH	7.17 *td* (7.2; 1.2)	127.09, CH	7.19 *td* (7.3; 1.3)
10	126.9, CH	7.31 *ddd* (7.9; 7.2; 1.1)	127.06, CH	7.29 *ddd* (7.9; 7.3; 1.7)
11	127.8, CH	8.31 *br dd* (7.9; 0.8)	127.81, CH	8.27 *br dd* (7.9; 0.8)
11a	131.9, qC		131.9, qC	
1-OCH_3_ (OH)			60.6, CH_3_	3.73 *s*
2-OCH_3_	60.8, CH_3_	3.95 *s*	60.9, CH_3_	3.95 *s*
3-OCH_3_	59.9, CH_3_	3.87 *s*	60.4, CH_3_	3.91 *s*

The data were obtained at 298 K with TMS as an internal reference (0.00 ppm) in CDCl_3_.

**Table 3:** Cytotoxic activity of the isolated compounds from the bark of *G. olivacea*.

Compounds	IC_50_, in µg mL^−1^ (µmol/L), and 95% CI ^a^			
	HepG2	KG-1a	HCT116	MRC-5
Isopiline (**1**)	N.T.	N.T.	>25 (>84.07)	>25 (>84.07)
*O*-Methylisopiline (**2**)	N.T.	N.T.	>25 (>80.28)	>25 (>80.28)
Melosmine (**3**)	N.T.	N.T.	16.77 (49.70) 10.42–27.02	23.72 (70.30) 18.79–33.97
9-hydroxyiguattescine (**4**)	>25 (>70.73)	N.T.	>25 (>70.73)	>25 (>70.73)
Dihydromelosmine (**5**)	>25 (>73.66)	N.T.	>25 (>73.66)	>25 (>73.66)
Lysicamine (**6**)	N.T.	N.T.	6.64 (22.79) 5.35–8.24	17.24 (59.18) 11.04–29.22
Acanthoic acid (**7**)	14.63 (48.37) 10.67–20.07	N.T.	21.25 (70.25) 13.25–34.10	>25 (>82.65)
Guattouregidine (**8**)	>25 (>73.23)	N.T.	>25 (>73.23)	>25 (>73.23)
Gouregine (**9**)	>25 (>70.74)	>25 (>70.74)	N.T.	>25 (>70.74)
Doxorubicin ^b^	0.04 (0.07) 0.03–0.05	0.22 (0.40) 0.15–0.33	0.20 (0.36) 0.11–0,35	1.42 (2.61) 0.22–2.12

^a^ Data are presented as IC_50_ values in µg mL^−1^ (μmol L^−1^) and their 95% confidence intervals (CIs) obtained by nonlinear regression from three independent experiments performed in duplicate, measured via the Alamar blue assay after 72 h of incubation. The cancer cell lines used were HepG2 (human hepatocellular carcinoma), KG-1a (human myeloid leukemia), and HCT116 (human colon carcinoma) cells. Noncancerous MRC-5 cells (human lung fibroblasts) were used. ^b^ Doxorubicin was used as a positive control. N.T: Not tested.

## Data Availability

The data presented in this study are available in the Supplementary Materials.

## Supplementary Materials

The following supporting information can be downloaded at: https://www.mdpi.com/article/10.3390/molecules29163834/s1, Figures S1–S115: ^1^H and ^13^C 1D and 2D NMR, IR and MS spectra for compounds **1**–**9**. Table S1: Selectivity indices (SI) for compounds **1**–**9**.
