# Supplementary material for: Gouregine, an α-Gem-Dimethyltetradehydrocularine Alkaloid, and Other Aporphinoid Alkaloids from the Bark of Guatteria olivacea (Annonaceae) and Their In Vitro Cytotoxic Activities
Source: Molecules. 2024 Aug 13;29(16):3834. doi: 10.3390/molecules29163834 (PMC11357160; doi:10.3390/molecules29163834)
Supplement: Supplementary file 1 [file molecules-29-03834-s001.zip › molecules-3106635-supplementary.pdf]

Supplementary Materials

Gouregine, an α-*gem*-dimethyltetradehydrocularine alkaloid, and other aporphinoid alkaloids from the bark of *Guatteria olivacea* (Annonaceae) and their in vitro cytotoxic activities

Emmanoel V. Costa [1,2*], José Guilherme C. Freitas [1], Steve Pereira Manickchand [1], Morgana de S. Araújo [1,2], Valdenizia R. Silva [3,] Luciano de S. Santos [3], Hector Henrique Ferreira Koolen [4], Felipe M. A. da Silva [5], Milena Botelho Pereira Soares [3,6], Daniel P. Bezerra [3,*]

[1] Department of Chemistry, Federal University of Amazonas (UFAM), Manaus 69080-900, AM, Brazil; evc@ufam.edu.br or emmanoelvc@gmail.com (E.V.C.); jguilhermefreitas.00@gmail.com (J.G.C.F.); stevemanickchand@gmail.com (S.P.M.); morgana.souza.araujo@gmail.com (M.d.S.A.)

[2] Postgraduate Program in Chemistry, Federal University of Amazonas (UFAM), Manaus 69080-900, AM, Brazil; evc@ufam.edu.br (E.V.C.); morgana.souza.araujo@gmail.com (M.d.S.A.)

[3] Gonçalo Moniz Institute, Oswaldo Cruz Foundation (IGM-FIOCRUZ/BA), Salvador 40296-710, BA, Brazil; valdeniziar@gmail.com (V.R.S.); luciano.biomed@gmail.com (L.d.S.S.); milena.soares@fiocruz.br (M.B.P.S.); daniel.bezerra@fiocruz.br (D.P.B.)

[4] Metabolomics and Mass Spectrometry Research Group, Amazonas State University (UEA), Manaus 690065-130, AM, Brazil; hkoolen@uea.edu.br (H.H.F.K.)

[5] Analytical Center - Multidisciplinary Support Center (CA-CAM), Federal University of Amazonas (UFAM), Manaus 69080-900, AM, Brazil; felipemourams@gmail.com (F.M.A.d.S.)

[6] SENAI Institute of Innovation (ISI) in Health Advanced Systems, University Center SENAI/CIMATEC, Salvador 41650-010, BA, Brazil; milena.soares@fiocruz.br (M.B.P.S.)

*Correspondence: evc@ufam.edu.br (E.V.C.); daniel.bezerra@fiocruz.br (D.P.B.); Tel./Fax: +55-92-3305-1181 (ext. 2870) (E.V.C.); +55-71-3176-2272 (D.P.B.)

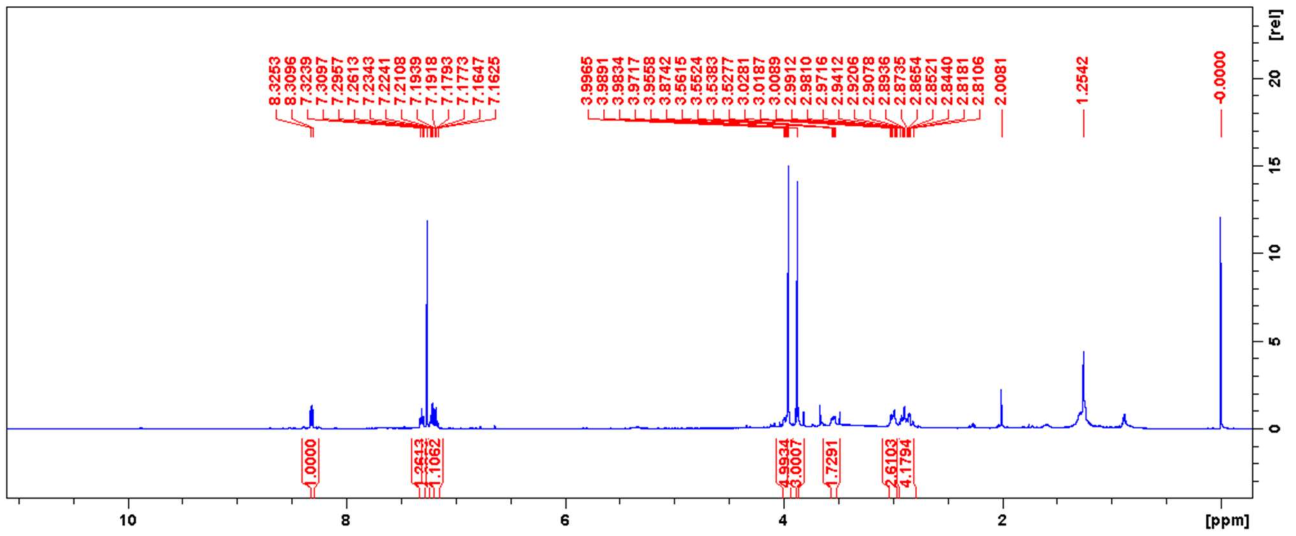

Figure S1. ^{1}H NMR spectrum of **isopiline** (**1**) in $CDCl_3$ at 500 MHz.

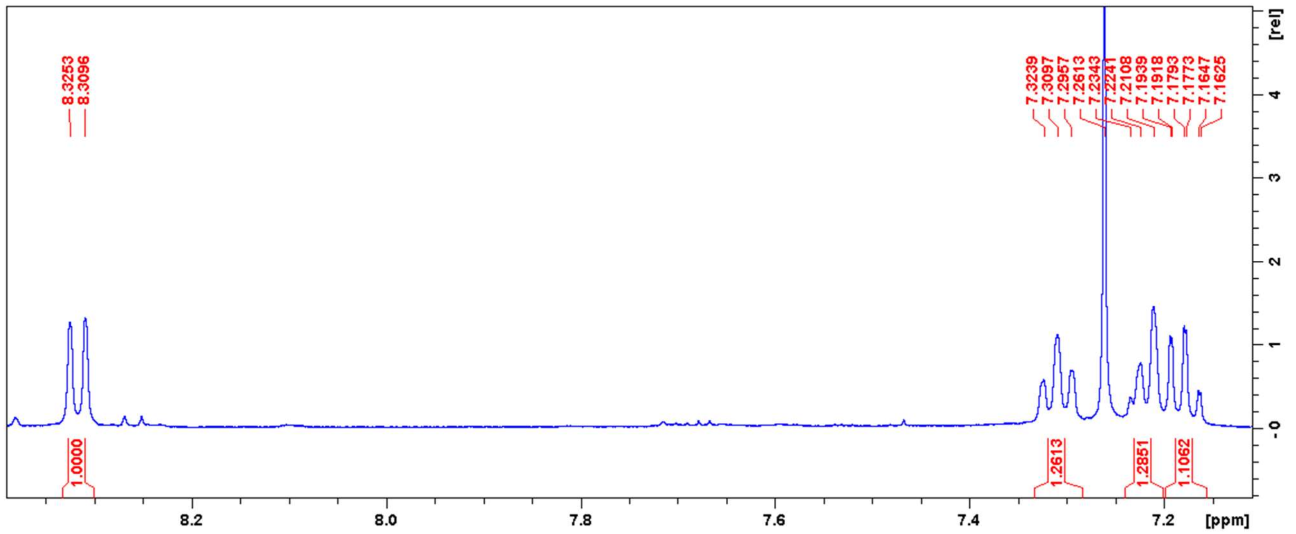

Figure S2. Expansion of aromatic hydrogen signals in the ^{1}H NMR spectrum of **isopiline** (**1**) in $CDCl_3$ at 500 MHz.

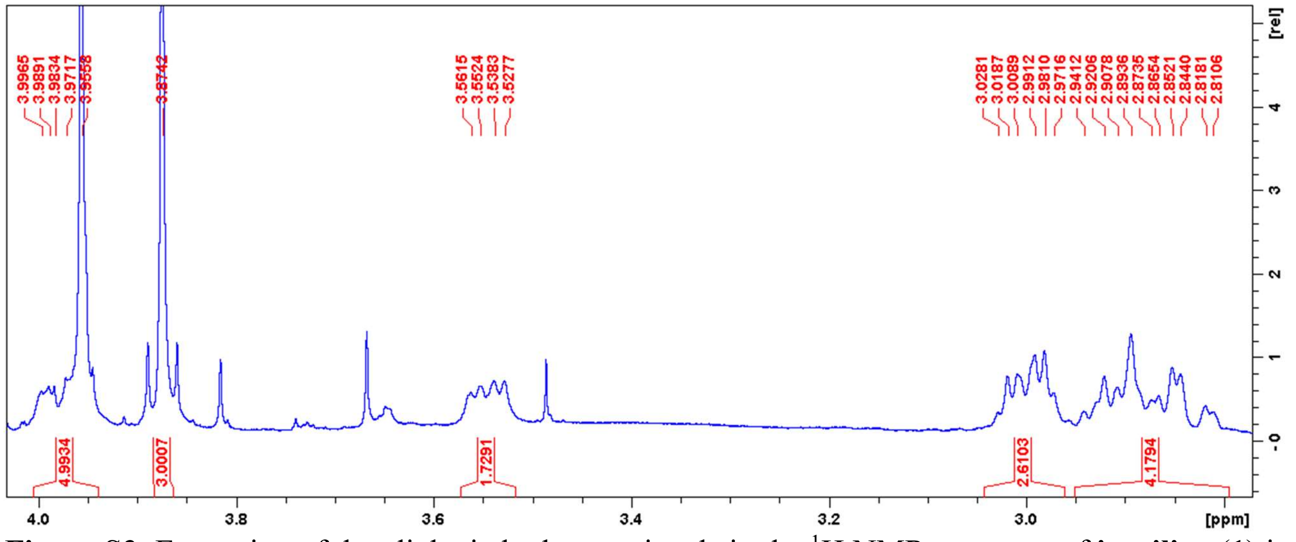

Figure S3. Expansion of the aliphatic hydrogen signals in the ^{1}H NMR spectrum of **isopiline** (**1**) in $CDCl_3$ at 500 MHz.

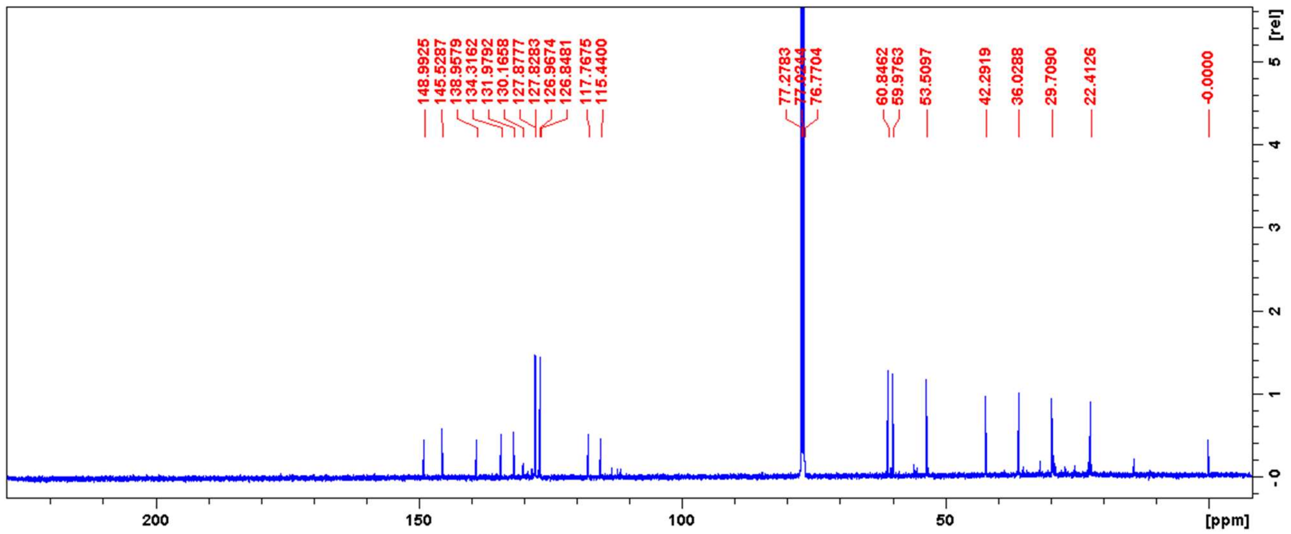

Figure S4. ^{13}C NMR spectrum of **isopiline** (**1**) in $CDCl_3$ at 125 MHz.

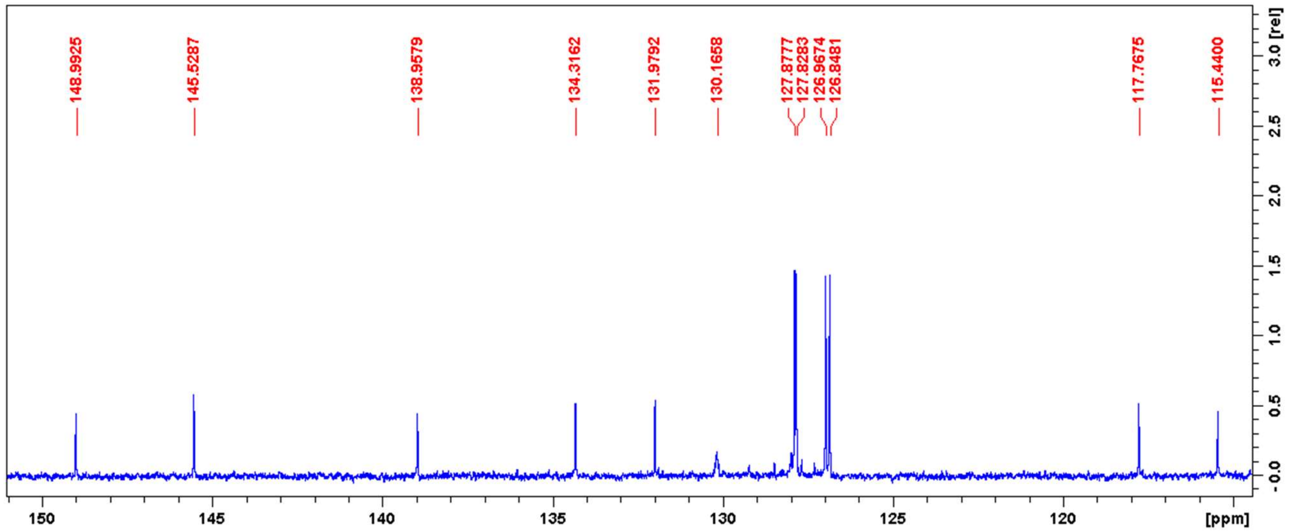

Figure S5. Expansion of aromatic carbon signals in the ^{13}C NMR spectrum of **isopiline** (**1**) in $CDCl_3$ at 125 MHz.

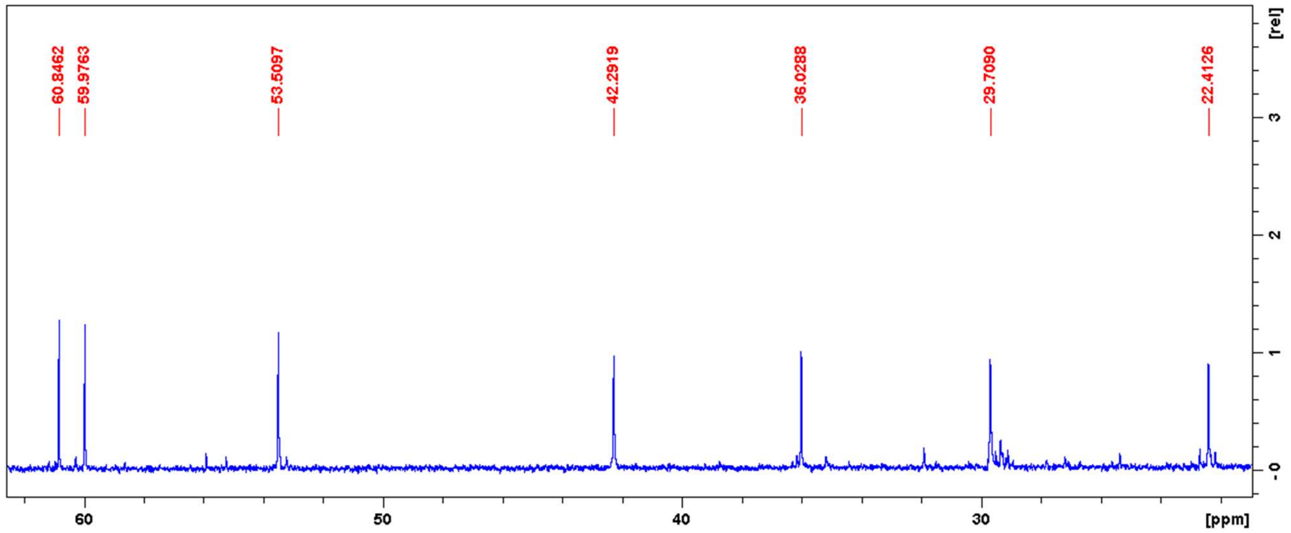

Figure S6. Expansion of the aliphatic carbon signals in the ^{13}C NMR spectrum of **isopiline** (**1**) in $CDCl_3$ at 125 MHz.

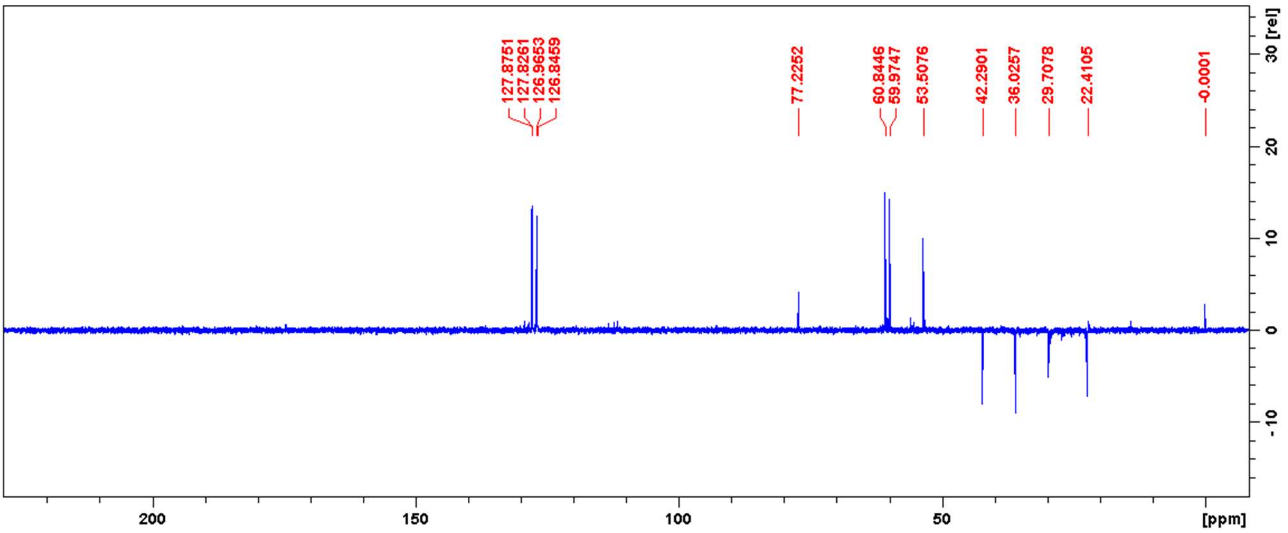

Figure S7. ^{13}C NMR DEPT 135 spectrum of **isopiline** (**1**) in $CDCl_3$ at 125 MHz.

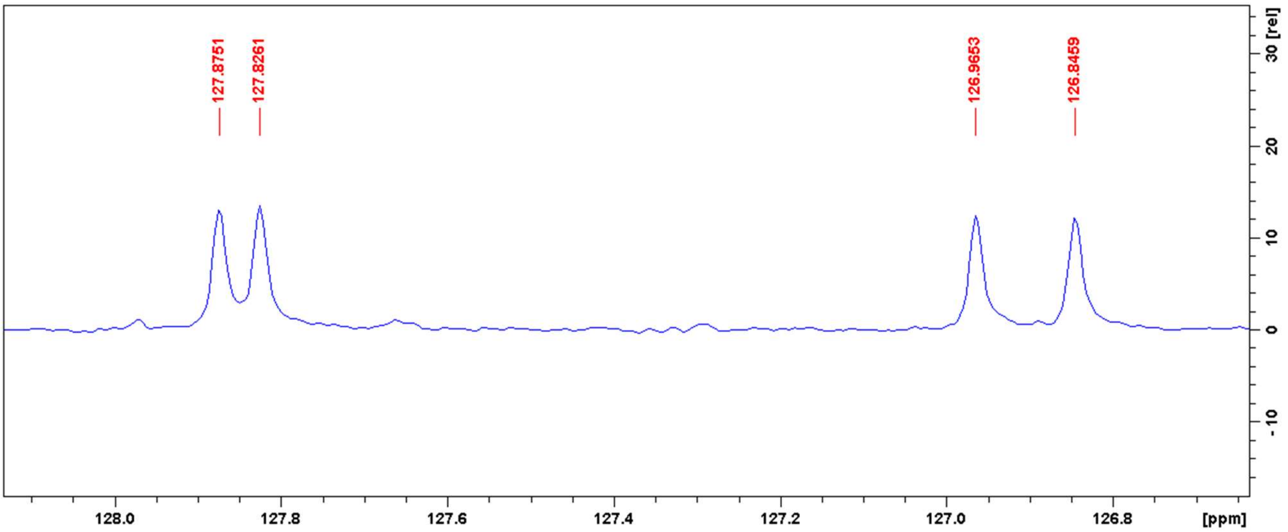

Figure S8. Expansion of aromatic carbon signals in the ^{13}C NMR DEPT 135 spectrum of **isopiline** (**1**) in $CDCl_3$ at 125 MHz.

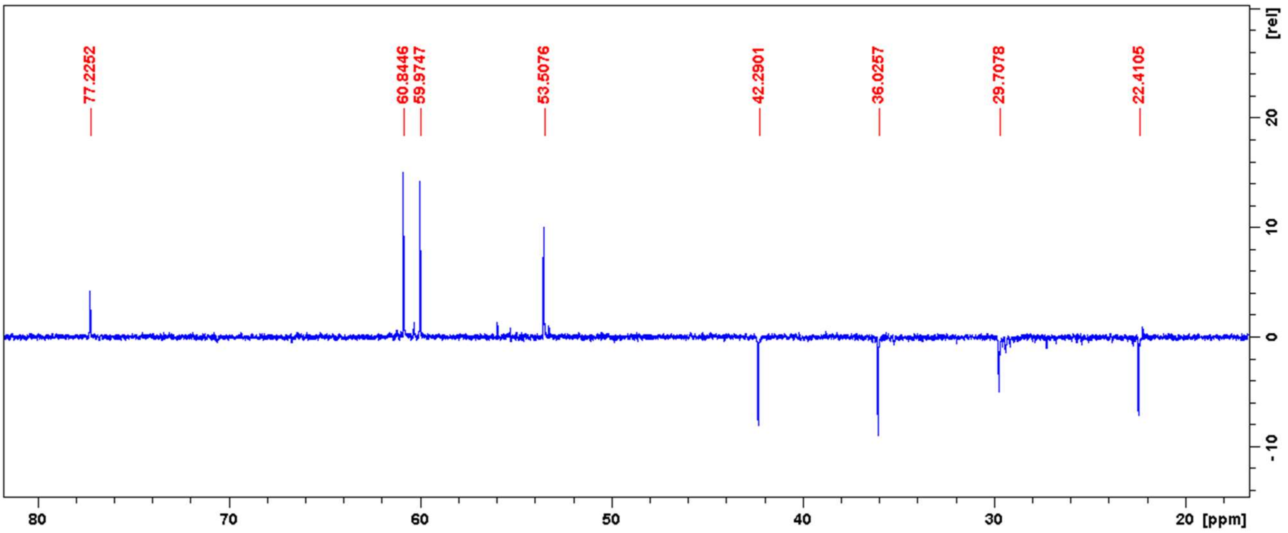

Figure S9. Expansion of the aliphatic carbon signals in the ^{13}C NMR DEPT 135 spectrum of **isopiline** (**1**) in $CDCl_3$ at 125 MHz.

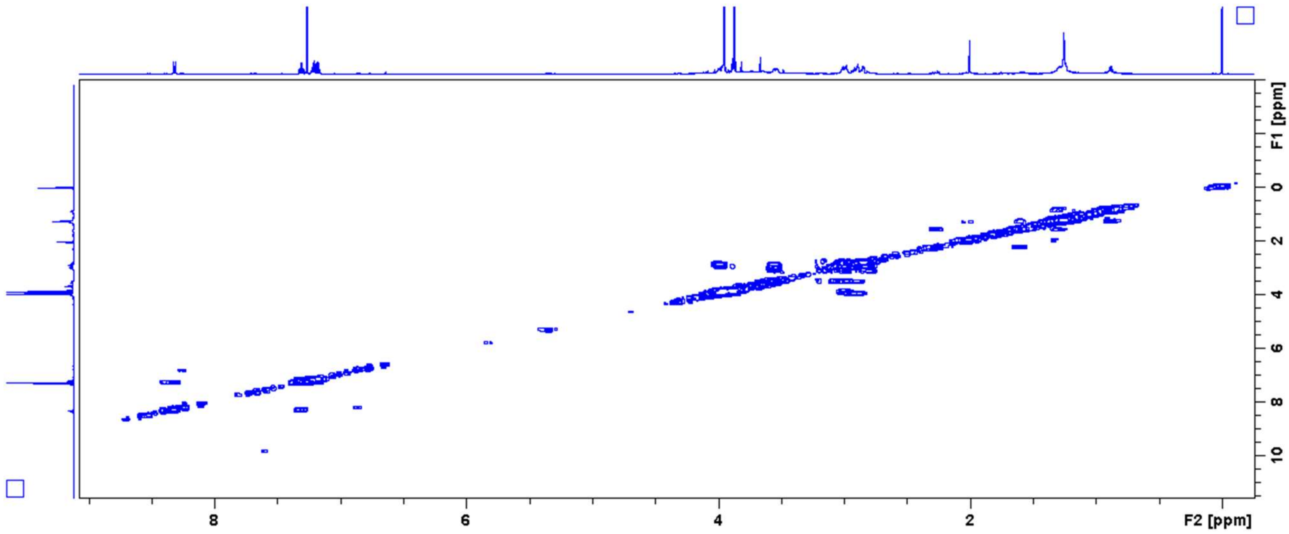

Figure S10. ^{1}H-^{1}H correlation map from the COSY NMR spectrum of **isopiline** (**1**) in $CDCl_3$ at 500 MHz.

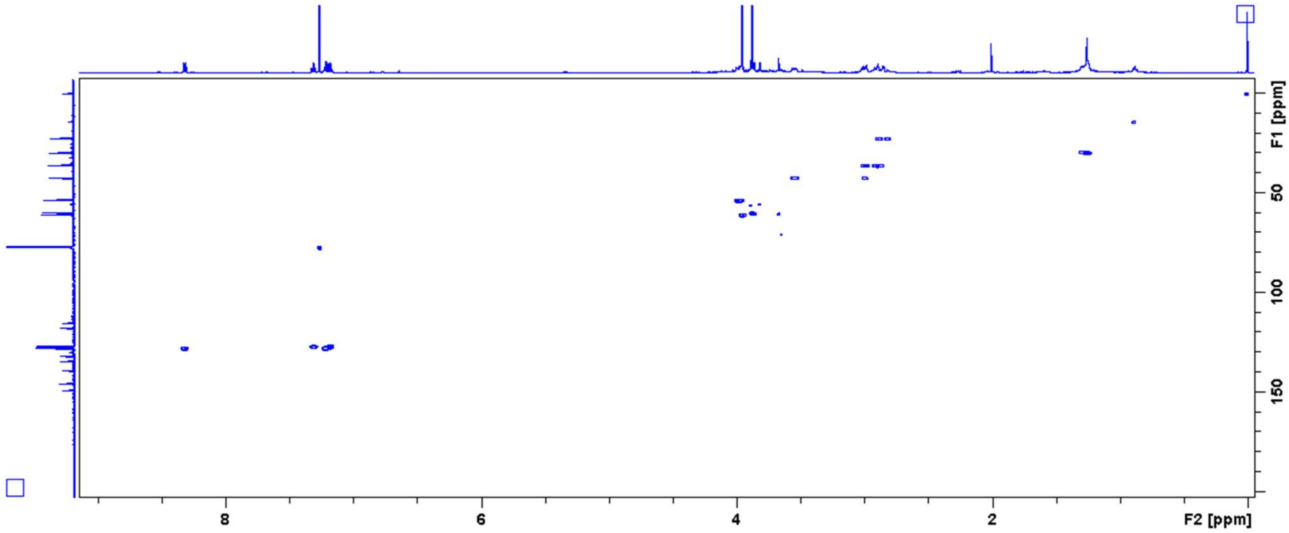

Figure S11. One-bond ^{1}H-^{13}C correlation map from the HSQC NMR spectrum of **isopiline** (**1**) in $CDCl_3$ at 500 (^{1}H) and 125 MHz (^{13}C).

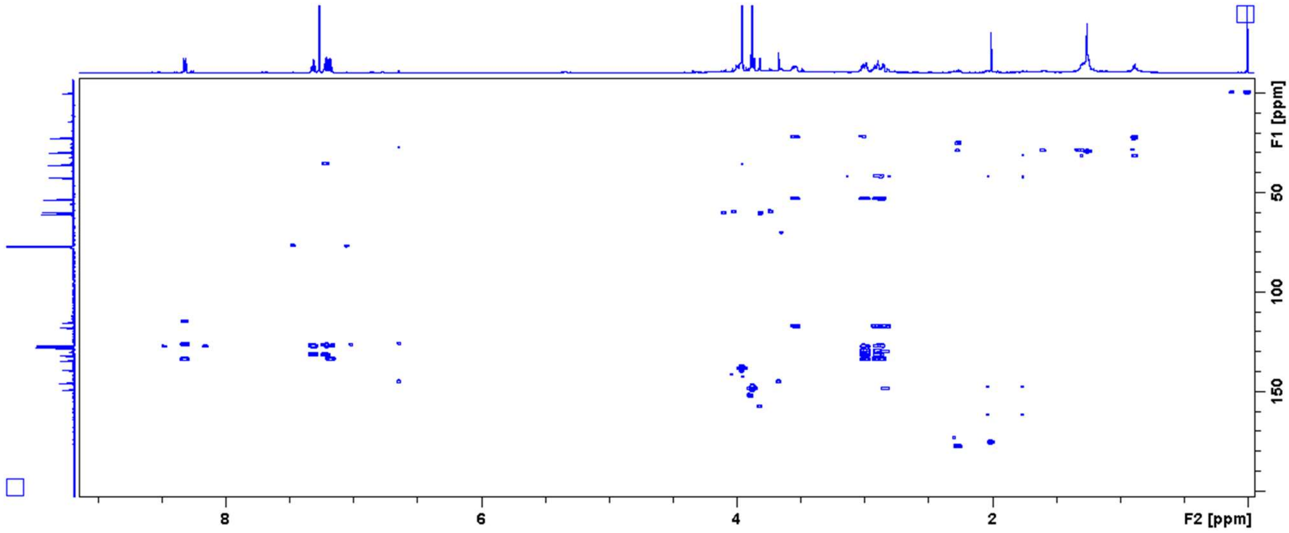

Figure S12. Long-range ^{1}H-^{13}C correlation map from the HMBC NMR spectrum of **isopiline** (**1**) in $CDCl_3$ at 500 (^{1}H) and 125 MHz (^{13}C).

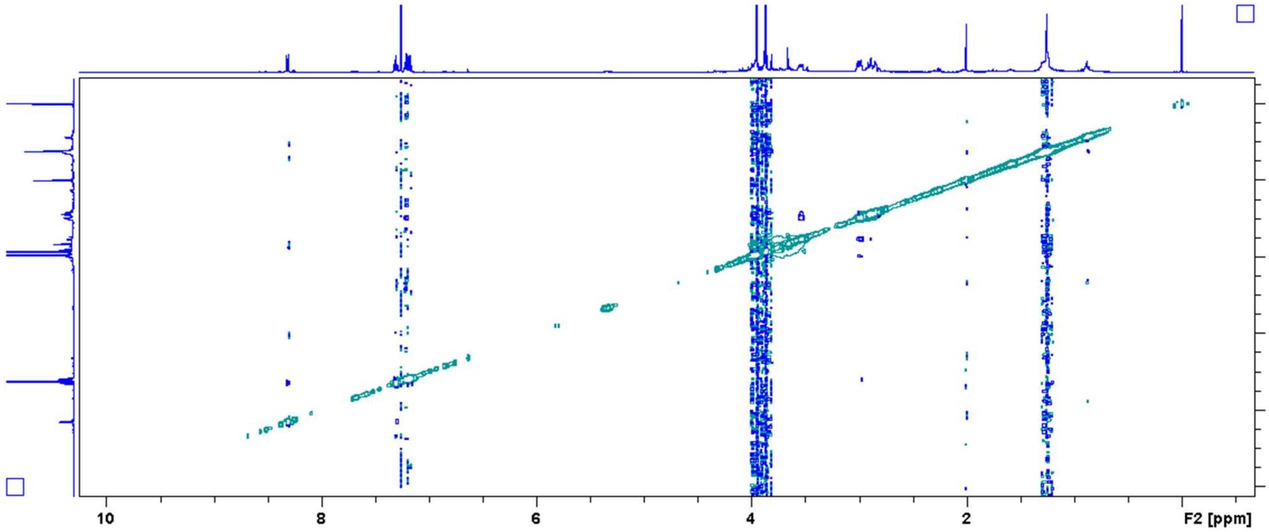

Figure S13. ^{1}H-^{1}H correlation map from NOESY NMR of **isopiline** (**1**) in $CDCl_3$ at 500 (^{1}H) and 125 MHz (^{13}C).

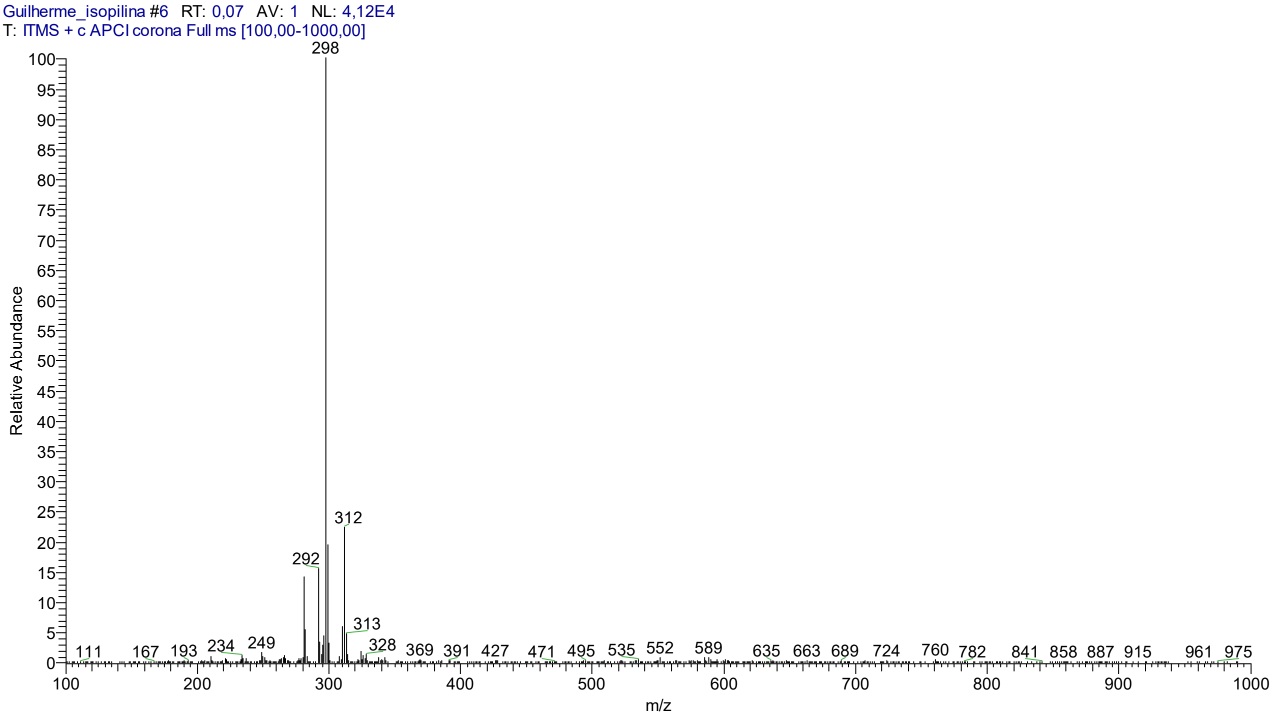

Figure S14. Full LR-APCI(+)-MS spectrum of **isopiline** (**1**) (*m/z* 298 $[M+H]^+$).

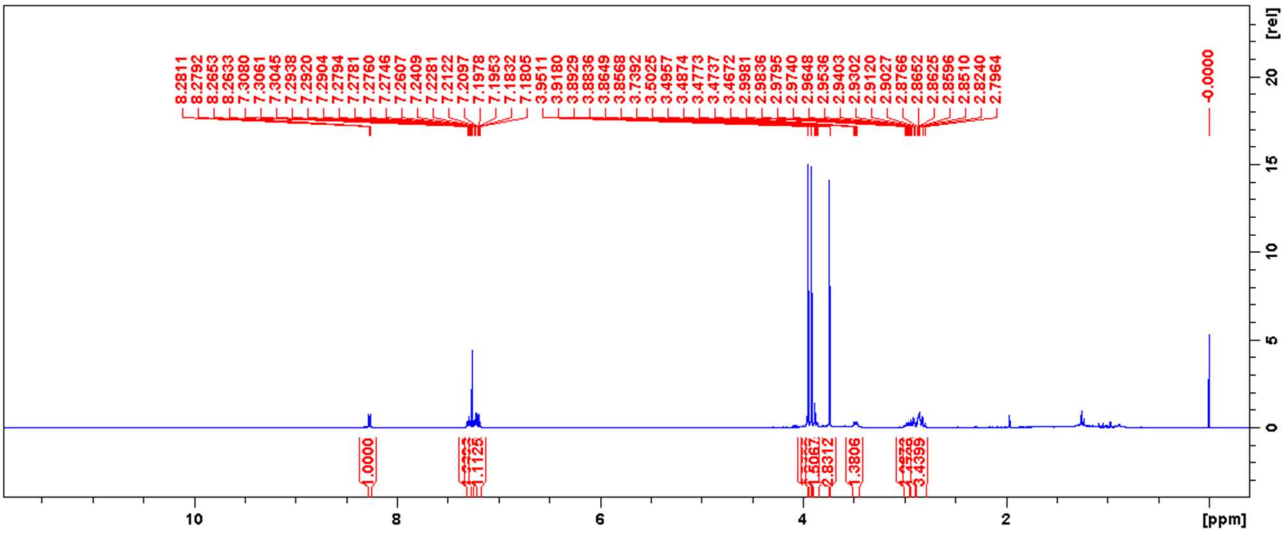

Figure S15. ^{1}H NMR spectrum of ***O*-methylisopiline** (**2**) in $CDCl_3$ at 500 MHz.

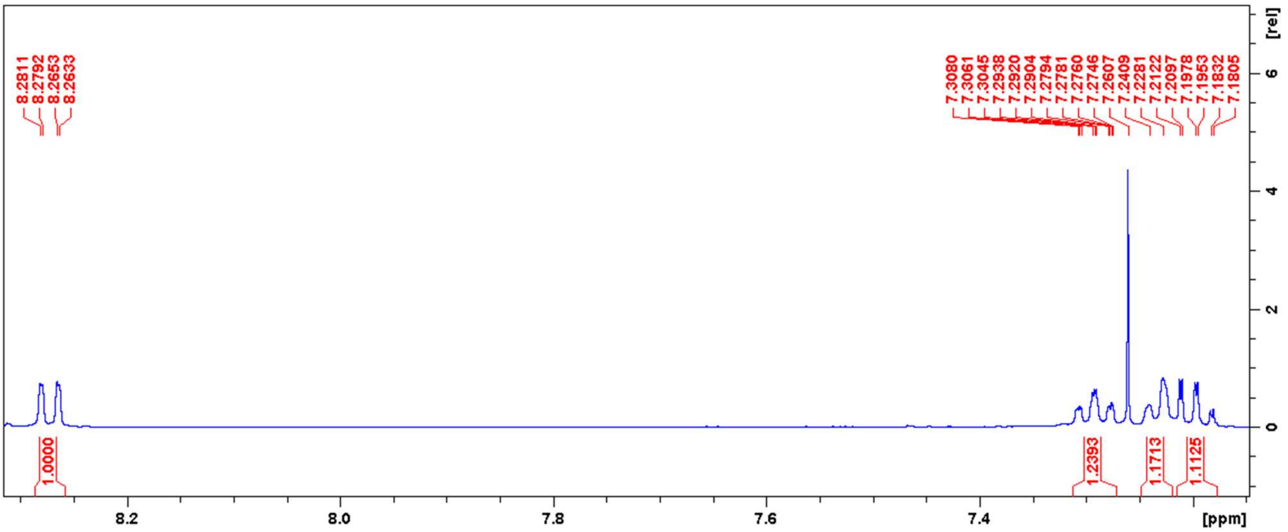

Figure S16. Expansion of aromatic hydrogen signals in the ^{1}H NMR spectrum of ***O*-methylisopiline** (**2**) in $CDCl_3$ at 500 MHz.

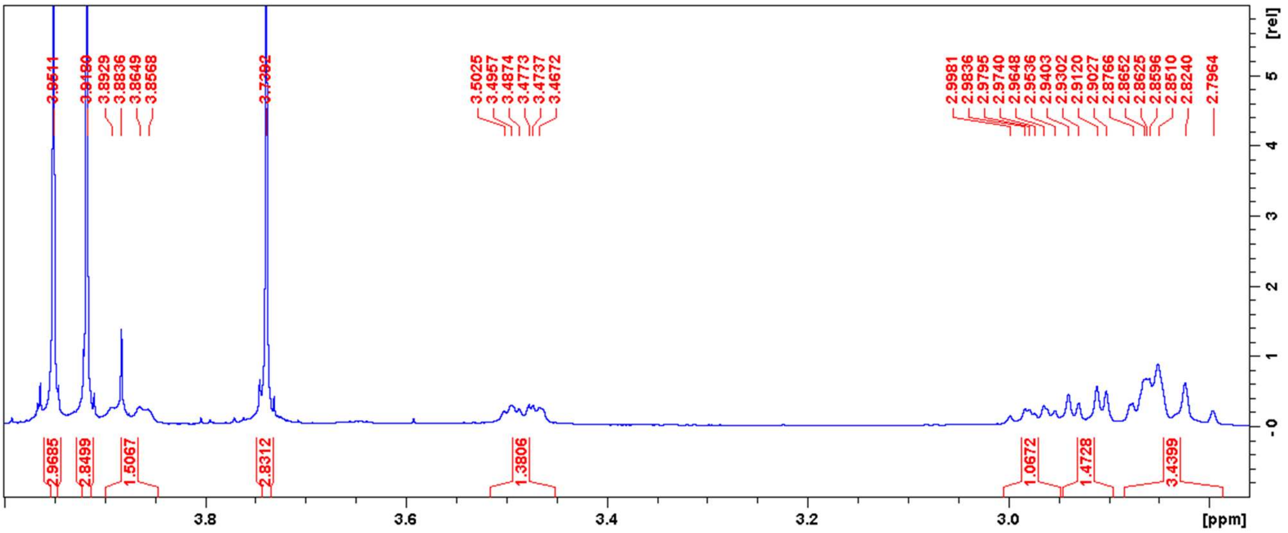

Figure S17. Expansion of the aliphatic hydrogen signals in the ^{1}H NMR spectrum of ***O*-methylisopiline** (**2**) in $CDCl_3$ at 500 MHz.

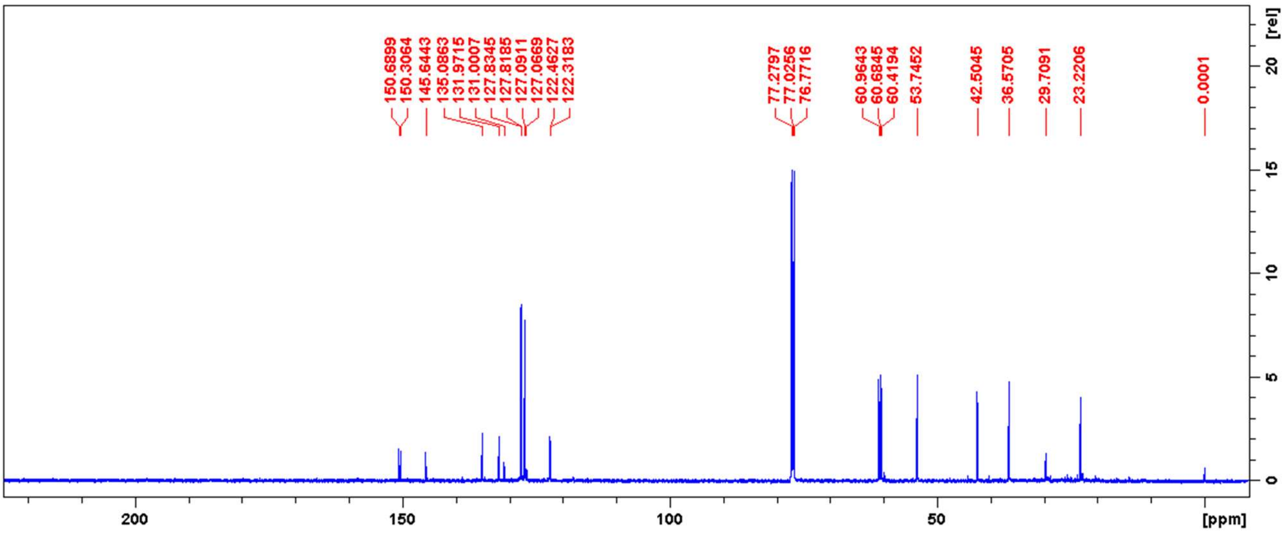

Figure S18. ^{13}C NMR spectrum of ***O*-methylisopiline** (**2**) in $CDCl_3$ at 125 MHz.

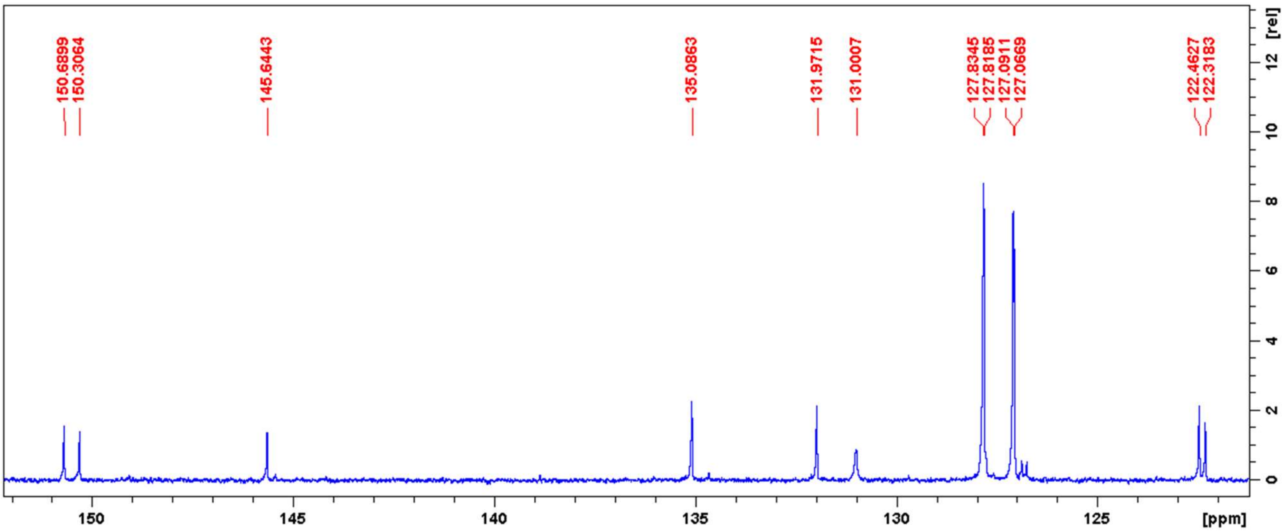

Figure S19. Expansion of aromatic carbon signals in the ^{13}C NMR spectrum of ***O*-methylisopiline** (**2**) in $CDCl_3$ at 125 MHz.

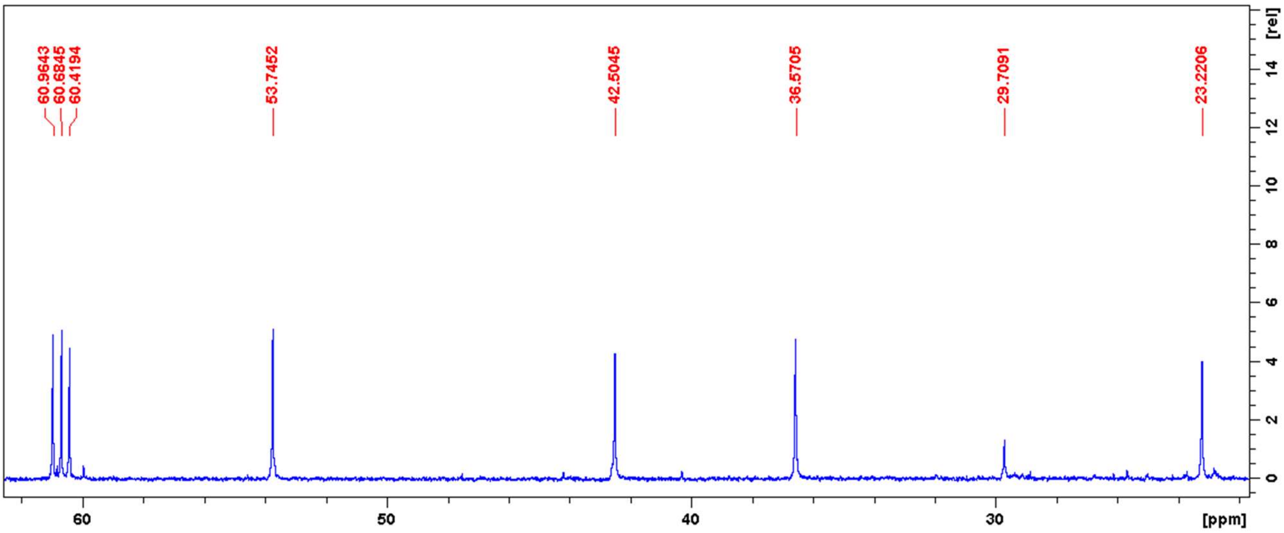

Figure S20. Expansion of the aliphatic carbon signals in the ^{13}C NMR spectrum of ***O*-methylisopiline** (**2**) in $CDCl_3$ at 125 MHz.

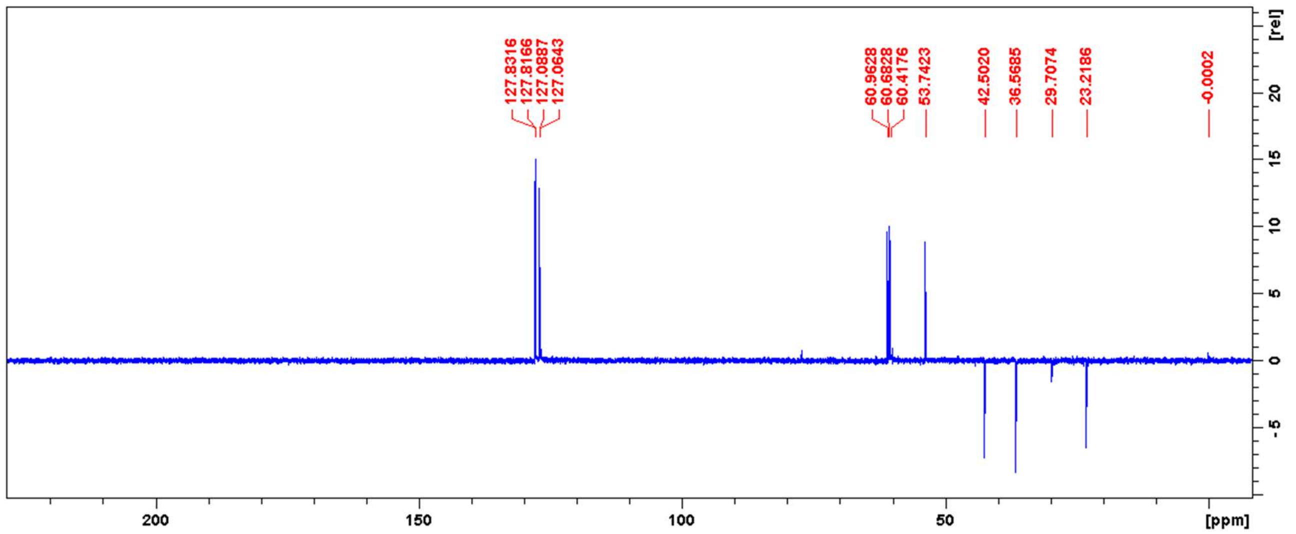

Figure S21. ^{13}C NMR DEPT 135 spectrum of ***O*-methylisopiline** (**2**) in $CDCl_3$ at 125 MHz.

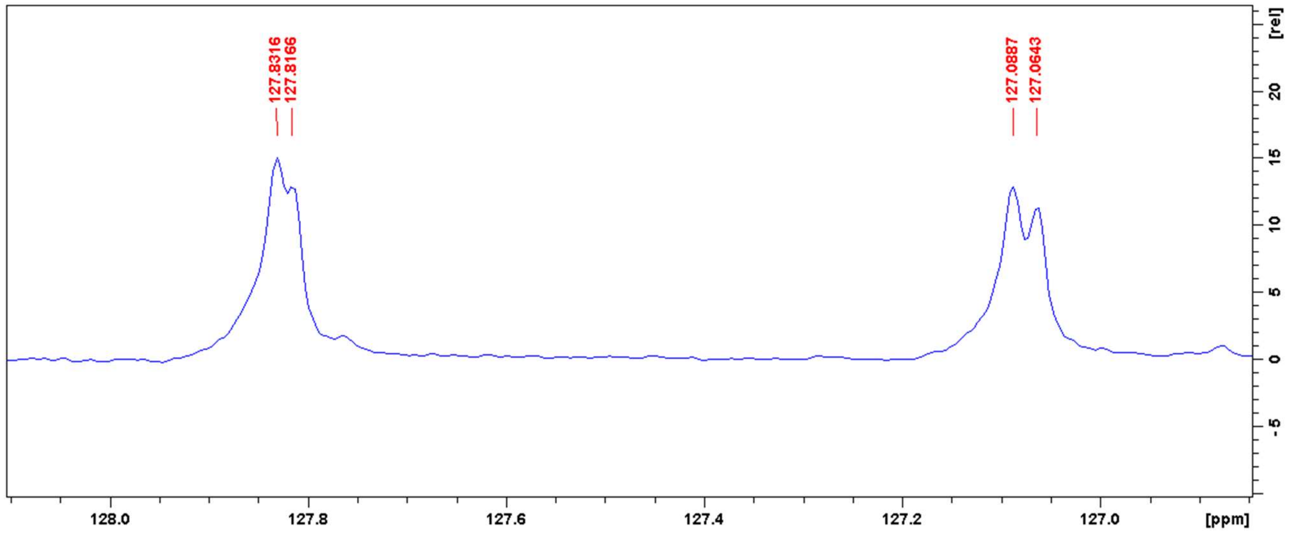

Figure S22. Expansion of aromatic carbon signals in the ^{13}C NMR DEPT 135 spectrum of ***O*-methylisopiline** (**2**) in $CDCl_3$ at 125 MHz.

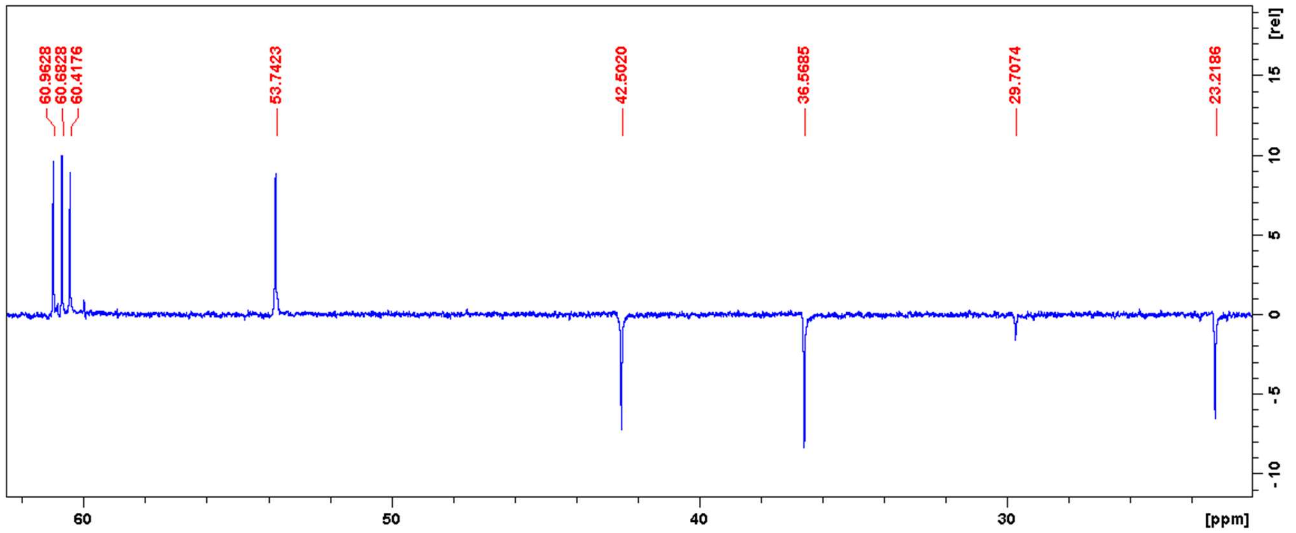

Figure S23. Expansion of the aliphatic carbon signals in the ^{13}C NMR DEPT 135 spectrum of ***O*-methylisopiline** (**2**) in $CDCl_3$ at 125 MHz.

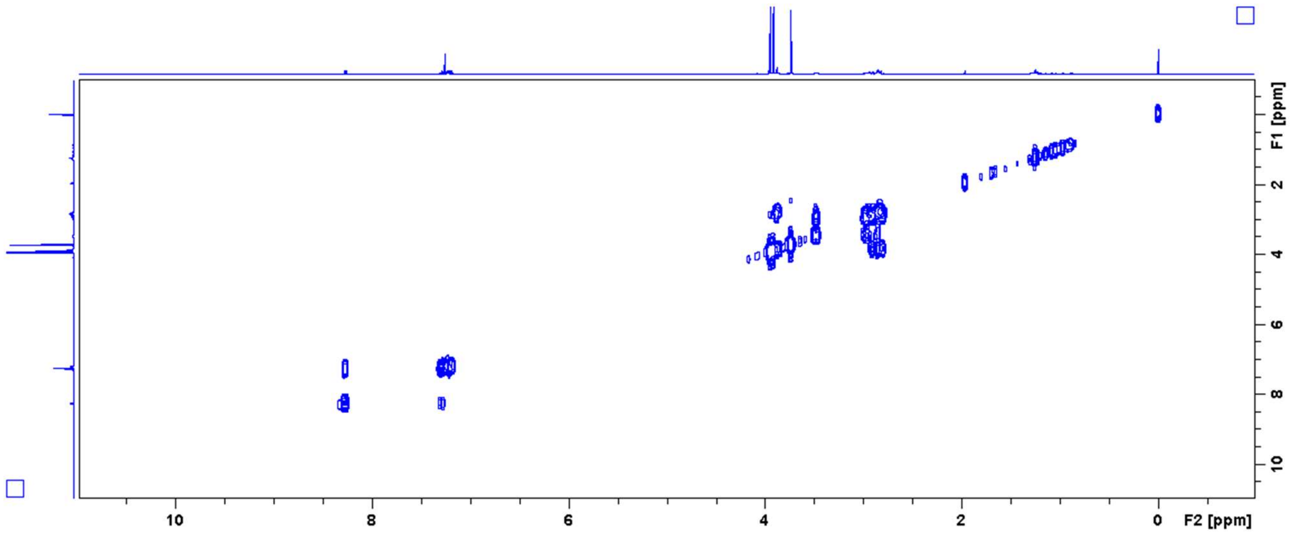

Figure S24. ^{1}H-^{1}H correlation map from the COSY NMR spectrum of ***O*-methylisopiline** (**2**) in $CDCl_3$ at 500 MHz.

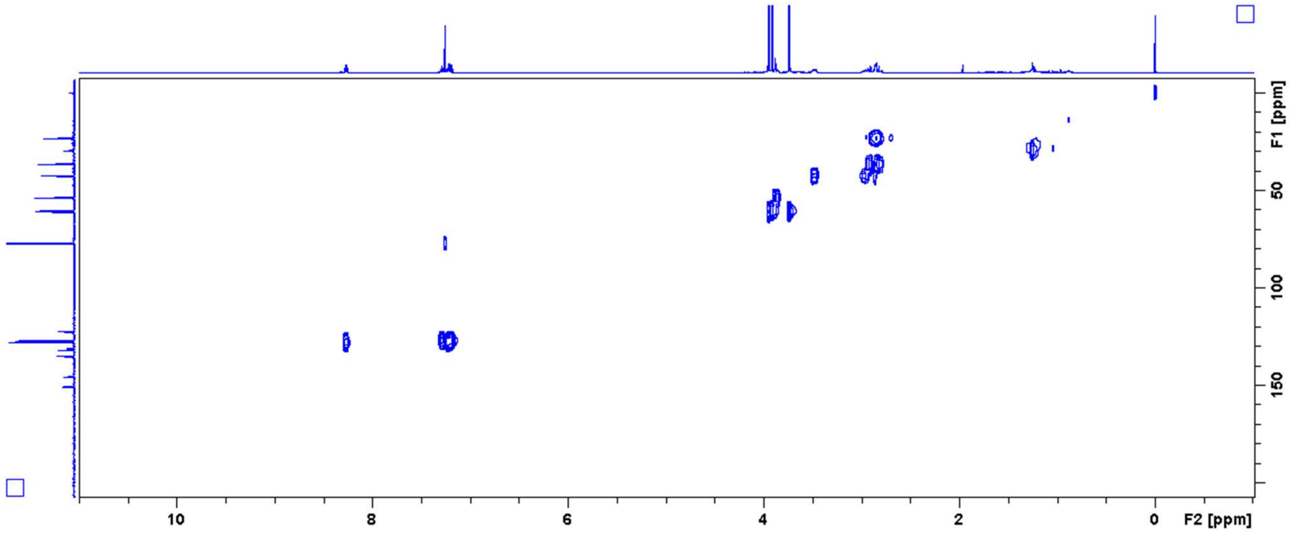

Figure S25. One-bond ^{1}H-^{13}C correlation map from the HSQC NMR spectrum of ***O*-methylisopiline** (**2**) in $CDCl_3$ at 500 (^{1}H) and 125 MHz (^{13}C).

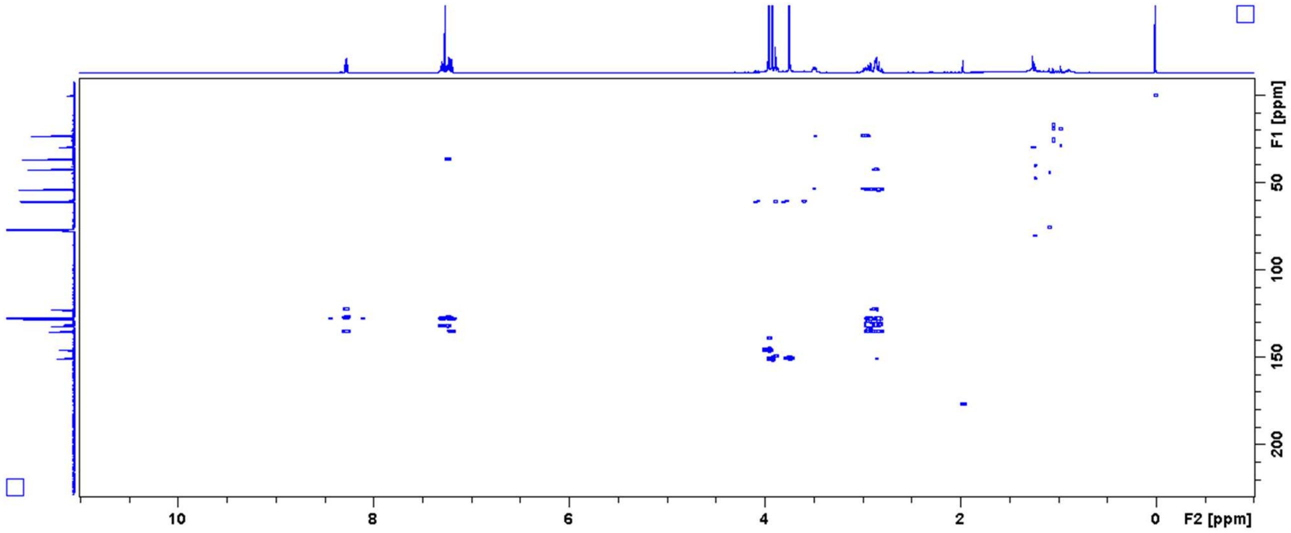

Figure S26. Long-range ^{1}H-^{13}C correlation map from the HMBC NMR spectrum of ***O*-methylisopiline** (**2**) in $CDCl_3$ at 500 (^{1}H) and 125 MHz (^{13}C).

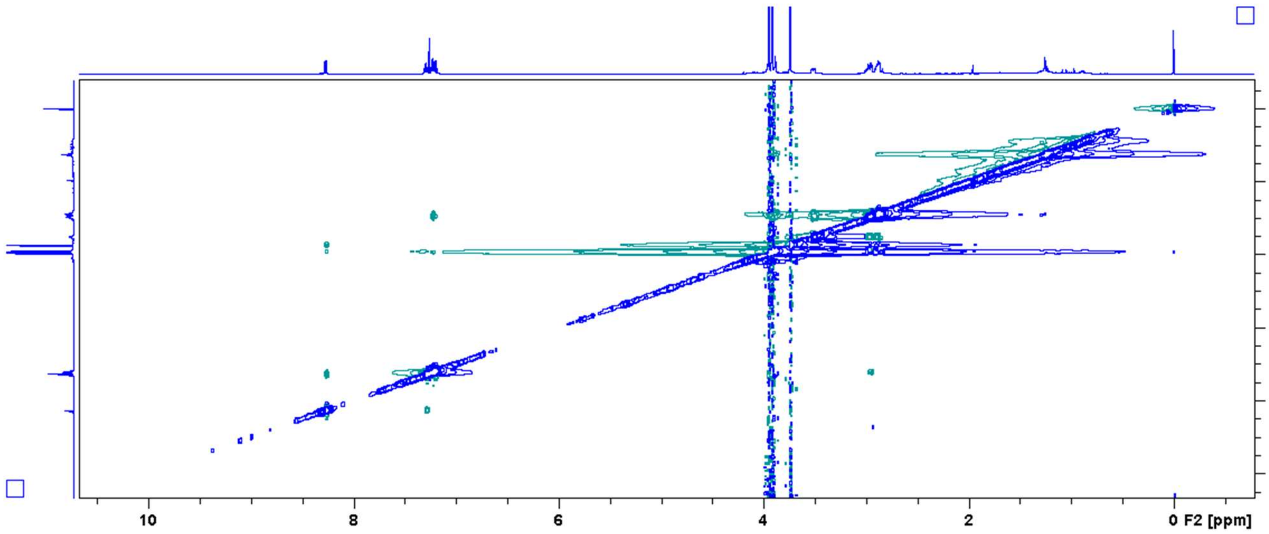

Figure S27. ^1H-^1H correlation map from NOESY NMR experiments of ***O*-methylisopiline** (**2**) in $CDCl_3$ at 500 (^1H) and 125 MHz (^{13}C).

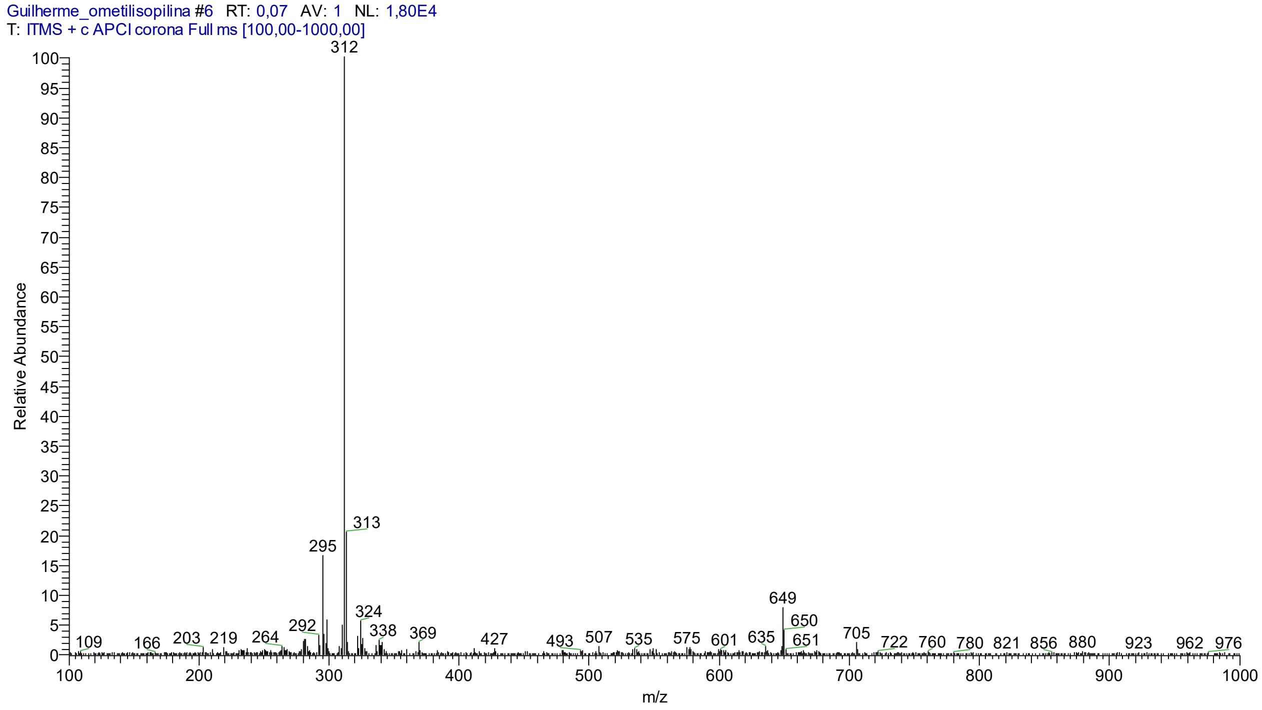

Figure S28. Full LR-APCI(+)-MS spectrum of ***O*-methylisopiline** (**2**) (*m/z* 312 [M+H]$^+$).

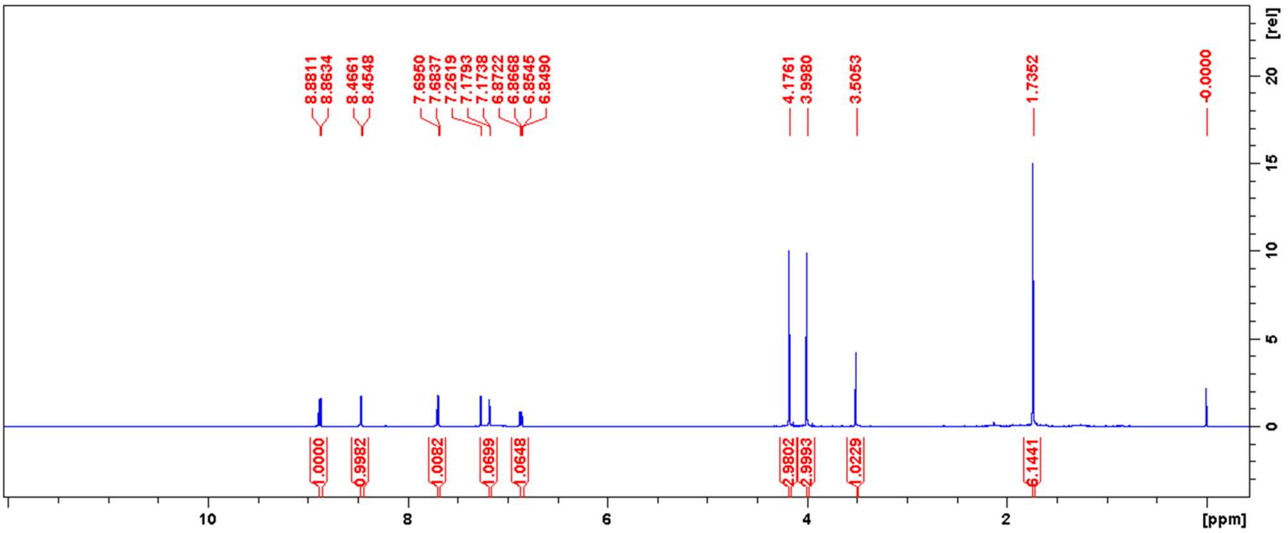

Figure S29. ^{1}H NMR spectrum of **melosmine** (**3**) in $CDCl_3$ at 500 MHz.

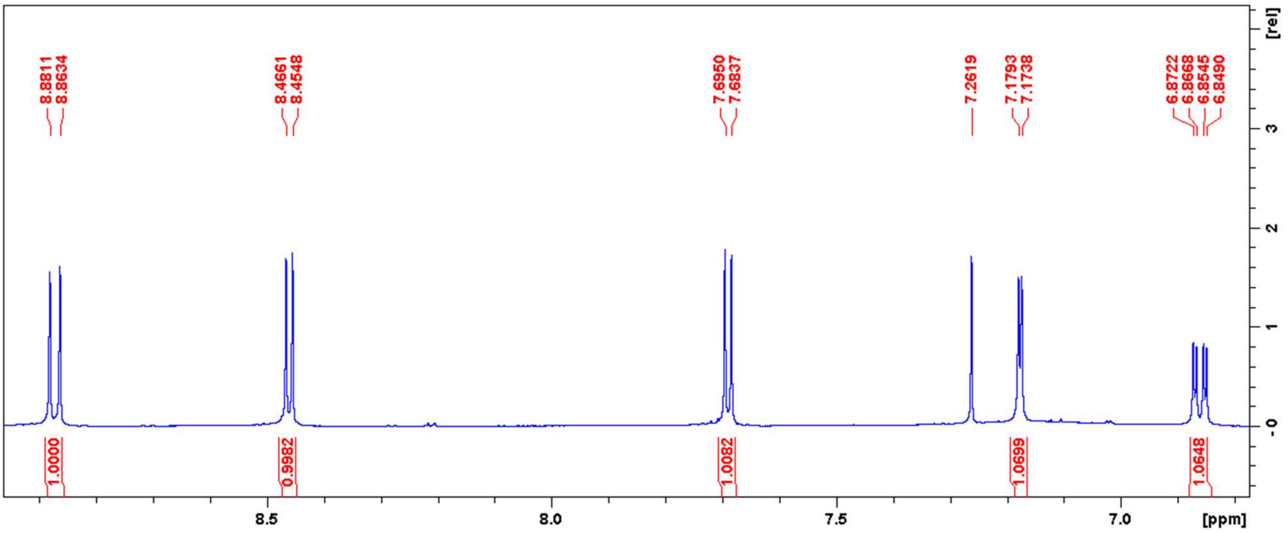

Figure S30. Expansion of aromatic hydrogen signals in the ^{1}H NMR spectrum of **melosmine** (**3**) in $CDCl_3$ at 500 MHz.

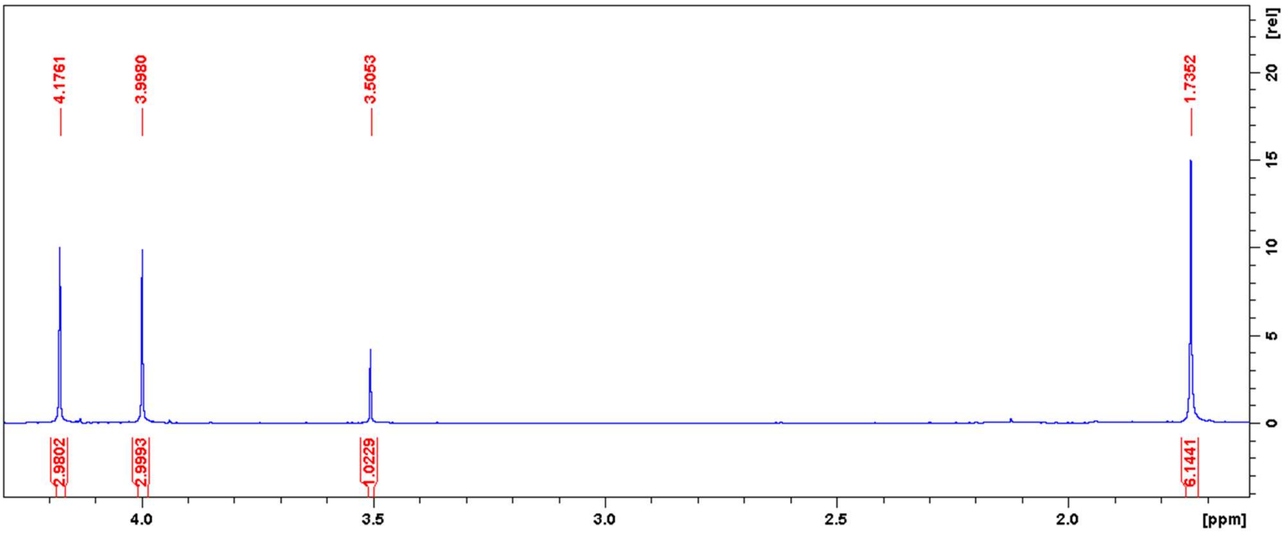

Figure S31. Expansion of the aliphatic hydrogen signals in the ^{1}H NMR spectrum of **melosmine** (**3**) in $CDCl_3$ at 500 MHz.

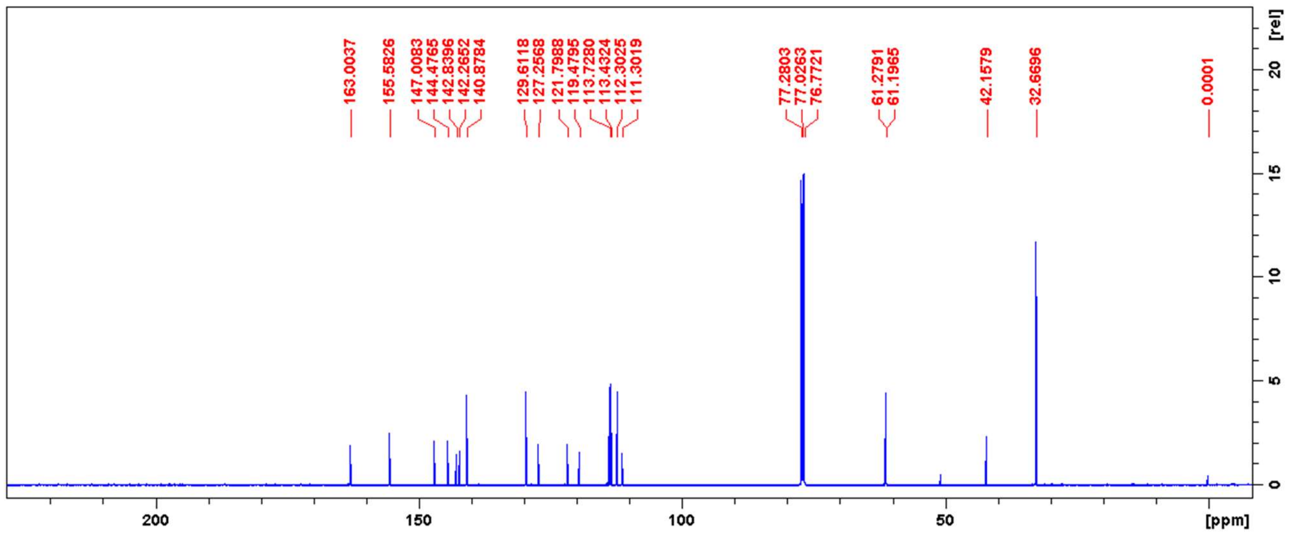

Figure S32. ^{13}C NMR spectrum of **melosmine** (**3**) in $CDCl_3$ at 125 MHz.

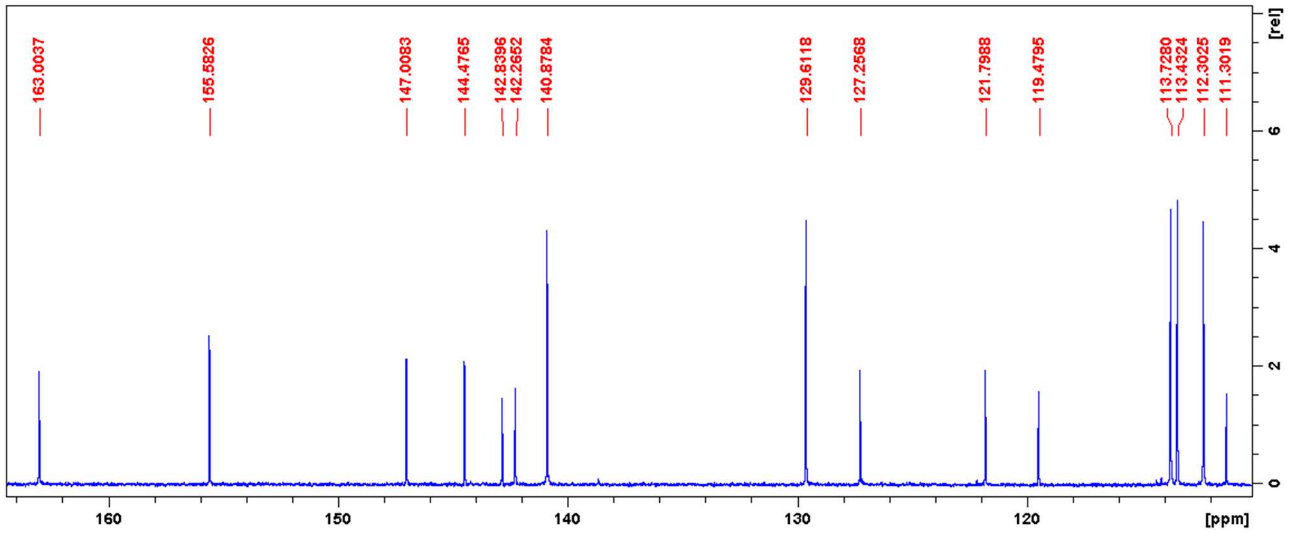

Figure S33. Expansion of aromatic carbon signals in the ^{13}C NMR spectrum of **melosmine** (**3**) in $CDCl_3$ at 125 MHz.

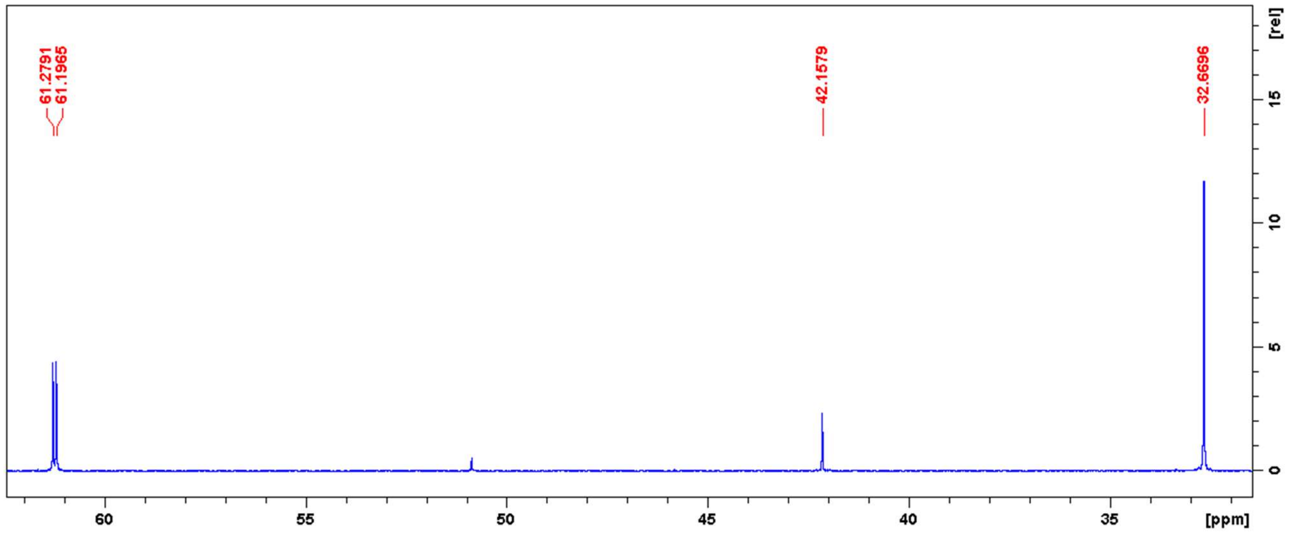

Figure S34. Expansion of the aliphatic carbon signals in the ^{13}C NMR spectrum of **melosmine** (**3**) in $CDCl_3$ at 125 MHz.

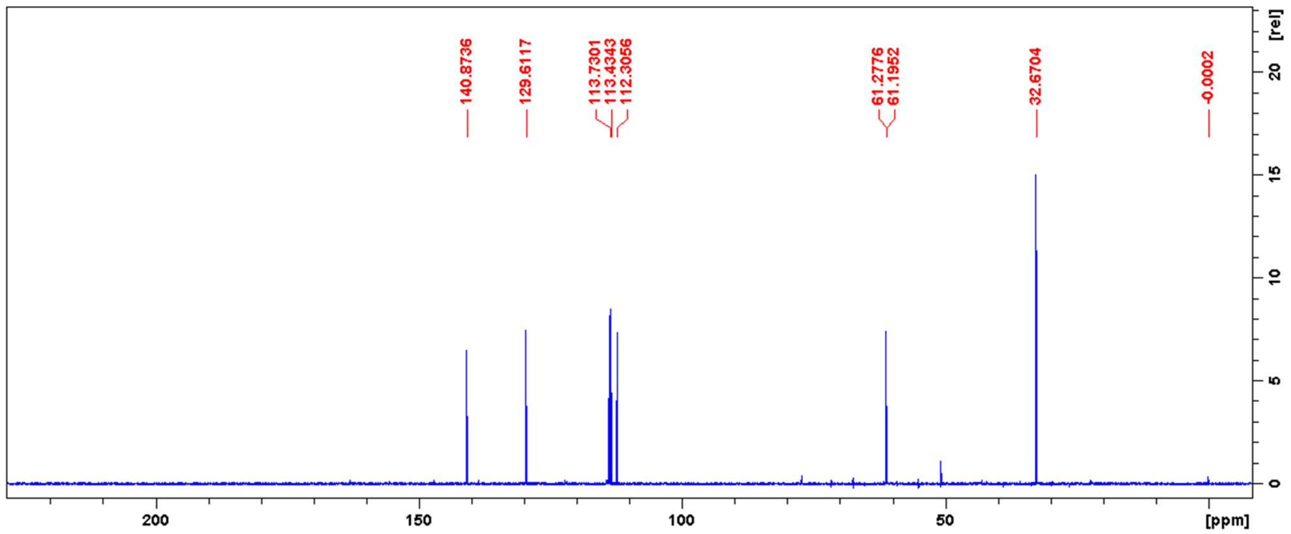

Figure S35. ^{13}C NMR DEPT 135 spectrum of **melosmine** (**3**) in $CDCl_3$ at 125 MHz.

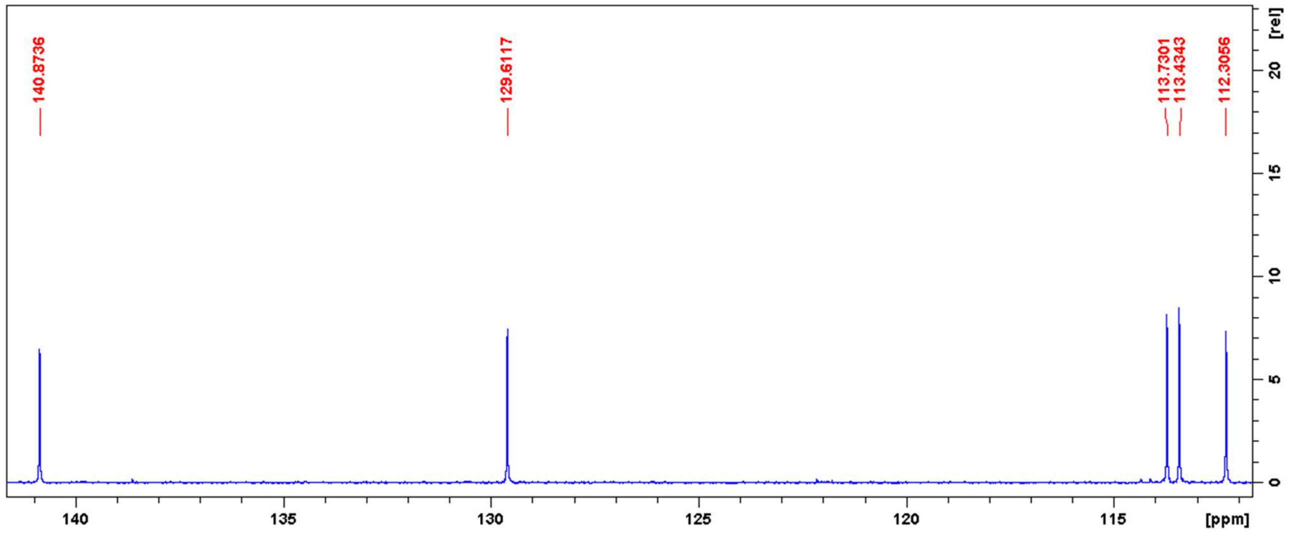

Figure S36. Expansion of aromatic carbon signals in the ^{13}C NMR DEPT 135 spectrum of **melosmine** (**3**) in $CDCl_3$ at 125 MHz.

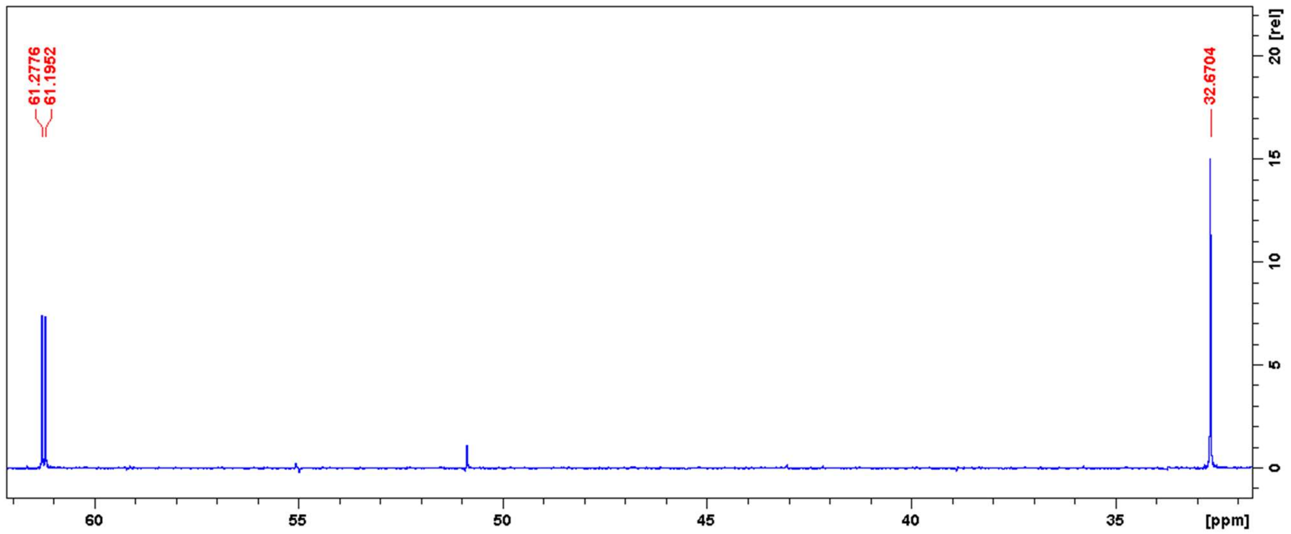

Figure S37. Expansion of the aliphatic carbon signals in the ^{13}C NMR DEPT 135 spectrum of **melosmine** (**3**) in $CDCl_3$ at 125 MHz.

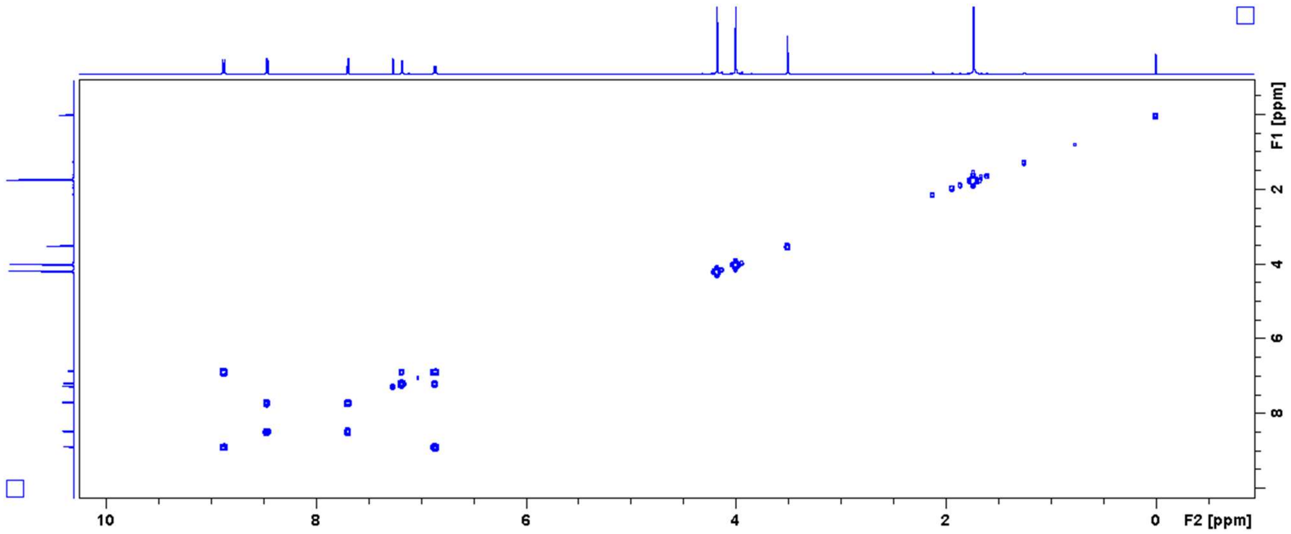

Figure S38. ^{1}H-^{1}H correlation map from the COSY NMR spectrum of **melosmine** (**3**) in $CDCl_3$ at 500 MHz.

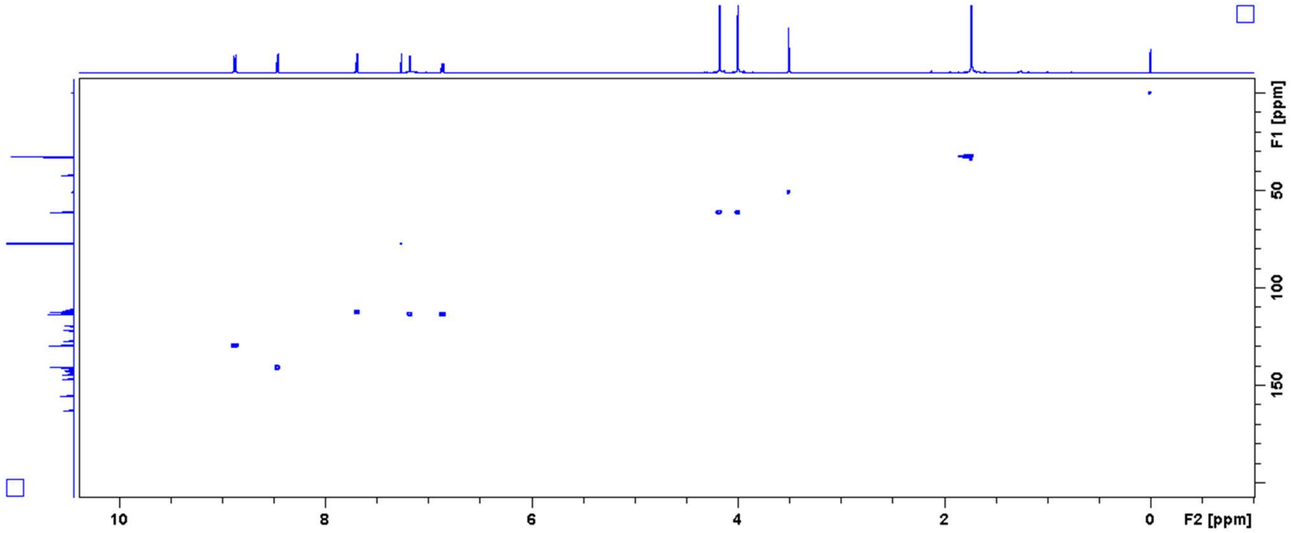

Figure S39. One-bond ^{1}H-^{13}C correlation map from the HSQC NMR spectrum of **melosmine** (**3**) in $CDCl_3$ at 500 (^{1}H) and 125 MHz (^{13}C).

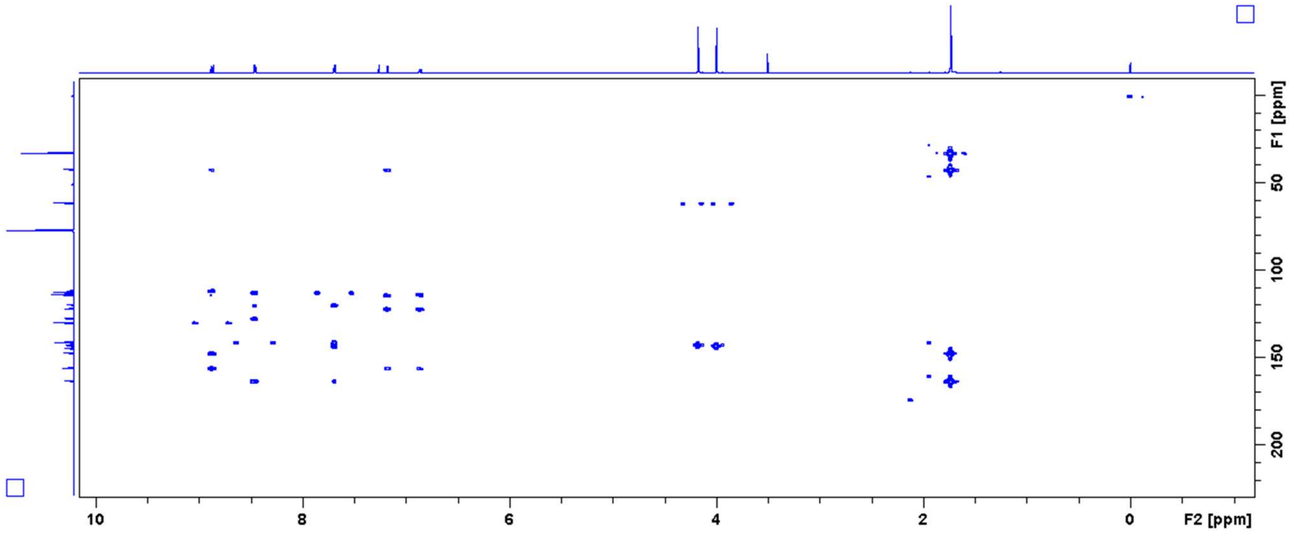

Figure S40. Long-range ^{1}H-^{13}C correlation map from the HMBC NMR spectrum of **melosmine** (**3**) in $CDCl_3$ at 500 (^{1}H) and 125 MHz (^{13}C).

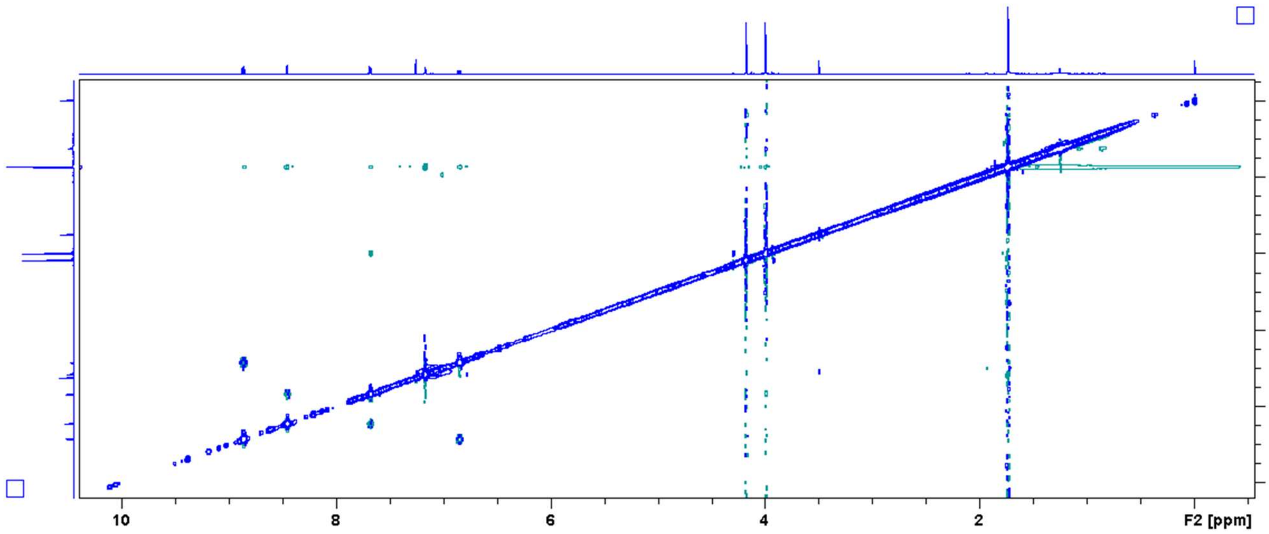

Figure S41. ^{1}H-^{1}H correlation map from NOESY NMR experiments of **melosmine** (**3**) in $CDCl_3$ at 500 (^{1}H) and 125 MHz (^{13}C).

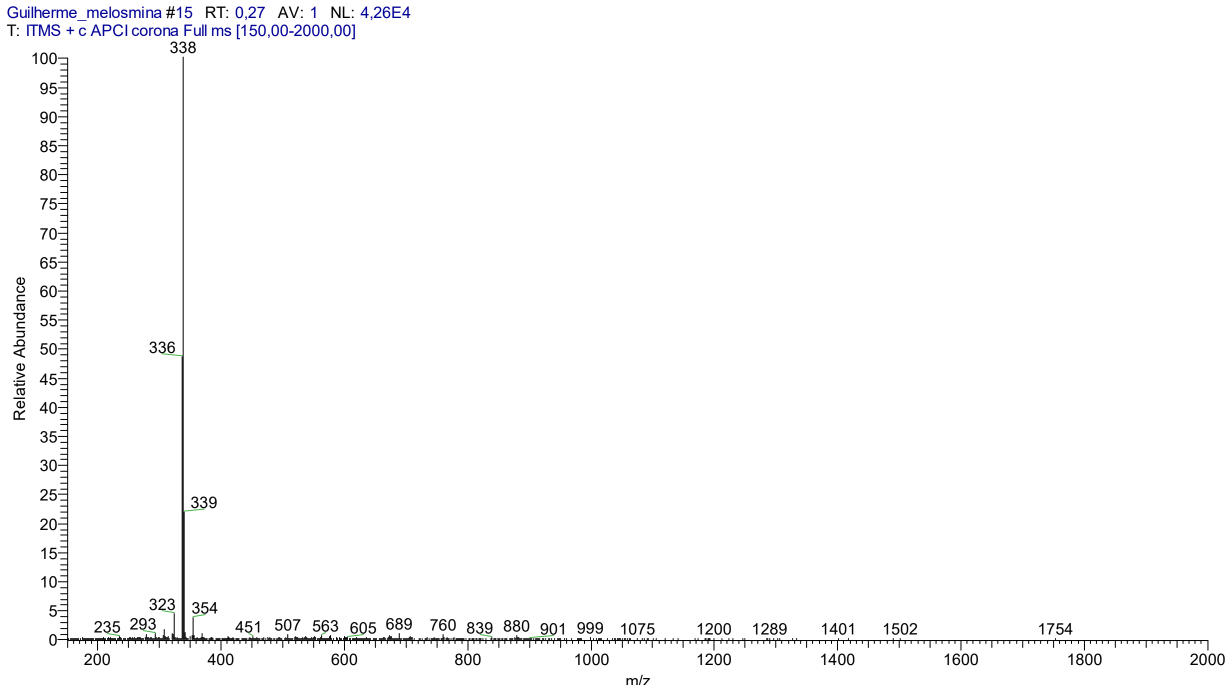

Figure S42. Full LR-APCI(+)-MS spectrum of **melosmine** (**3**) (*m/z* 338 $[M+H]^{+}$).

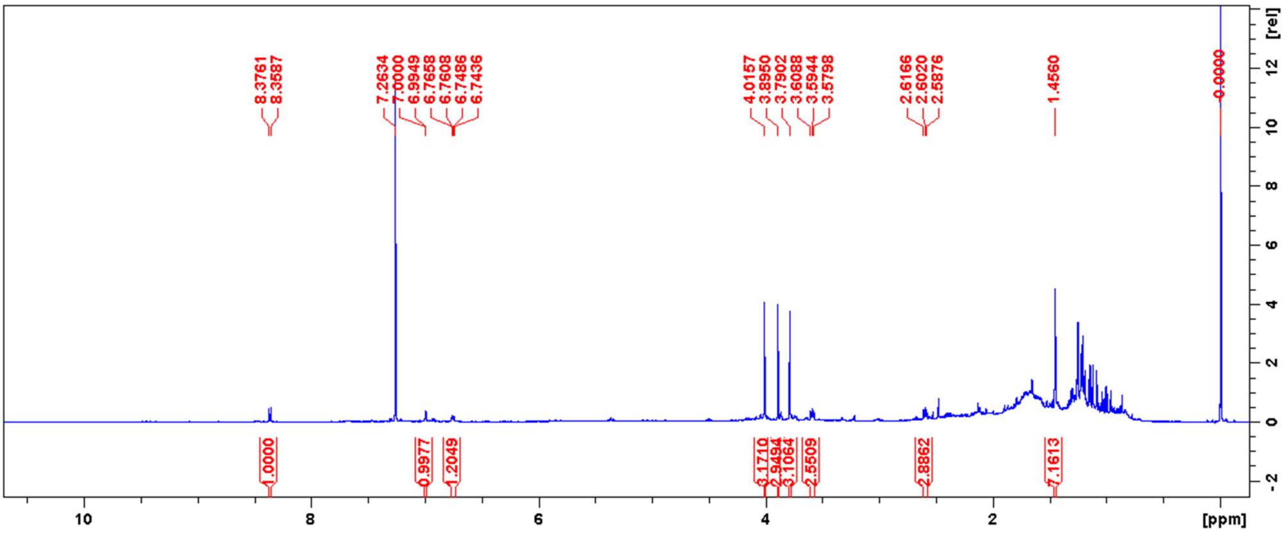

Figure S43. ^{1}H NMR spectrum of **9-hydroxyiguattescine** (**4**) in $CDCl_3$ at 500 MHz.

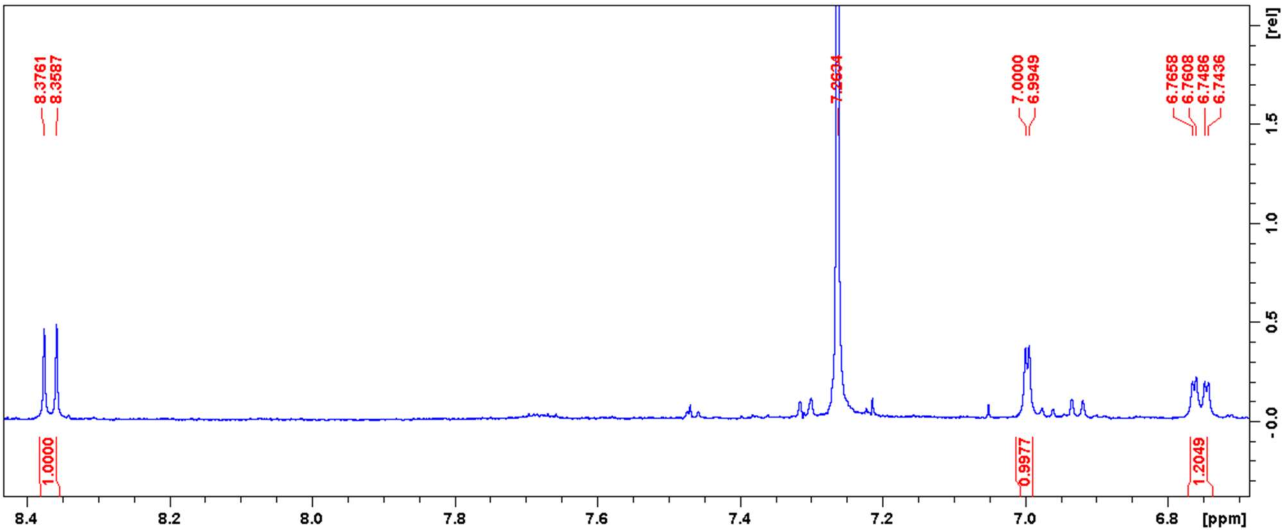

Figure S44. Expansion of aromatic hydrogen signals in the ^{1}H NMR spectrum of **9-hydroxyiguattescine** (**4**) in $CDCl_3$ at 500 MHz.

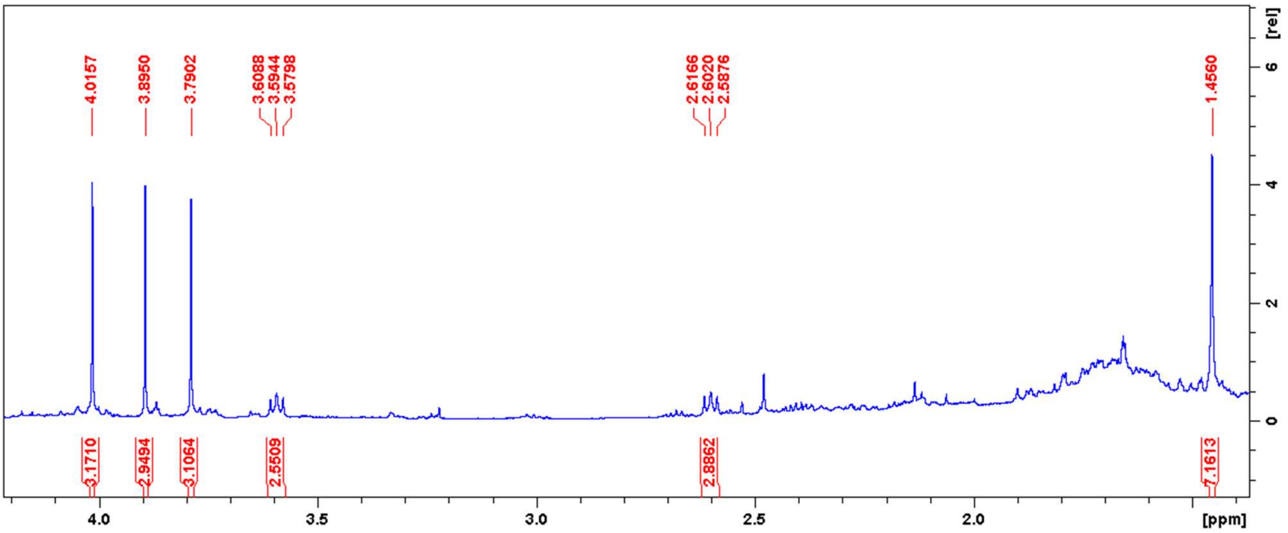

Figure S45. Expansion of the aliphatic hydrogen signals in the ^{1}H NMR spectrum of **9-hydroxyiguattescine** (**4**) in $CDCl_3$ at 500 MHz.

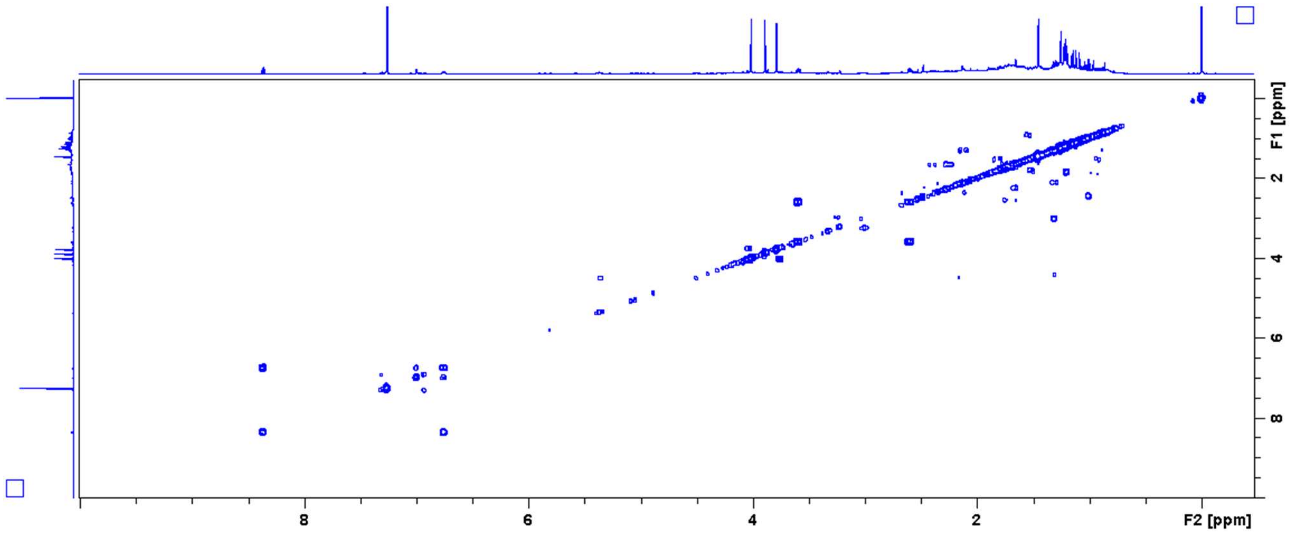

Figure S46. ^{1}H-^{1}H correlation map from the COSY NMR spectrum of **9-hydroxyiguattescine** (**4**) in $CDCl_3$ at 500 MHz.

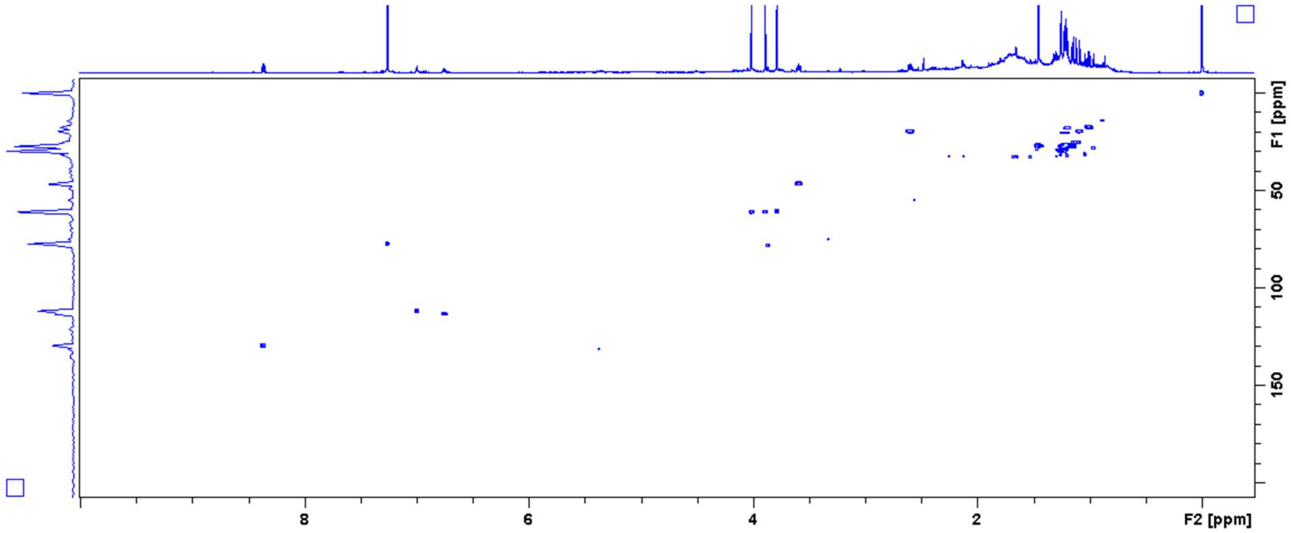

Figure S47. One-bond ^{1}H-^{13}C correlation map from the HSQC NMR spectrum of **9-hydroxyiguattescine** (**4**) in $CDCl_3$ at 500 (^{1}H) and 125 MHz (^{13}C).

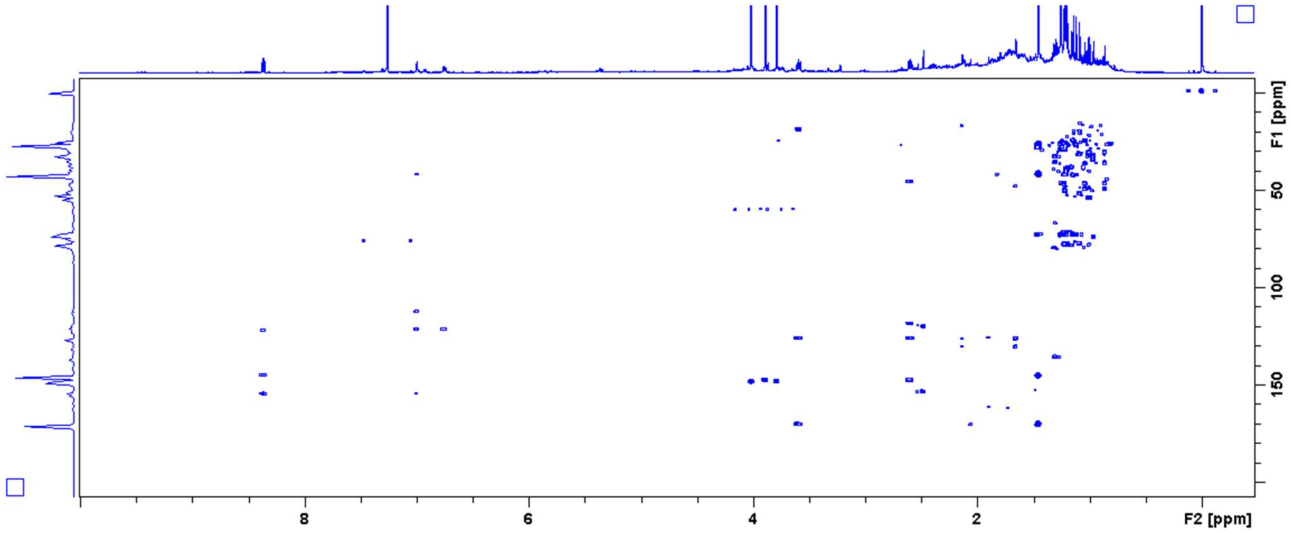

Figure S48. Long-range ^{1}H-^{13}C correlation map from the HMBC NMR spectrum of **9-hydroxyiguattescine** (**4**) in $CDCl_3$ at 500 (^{1}H) and 125 MHz (^{13}C).

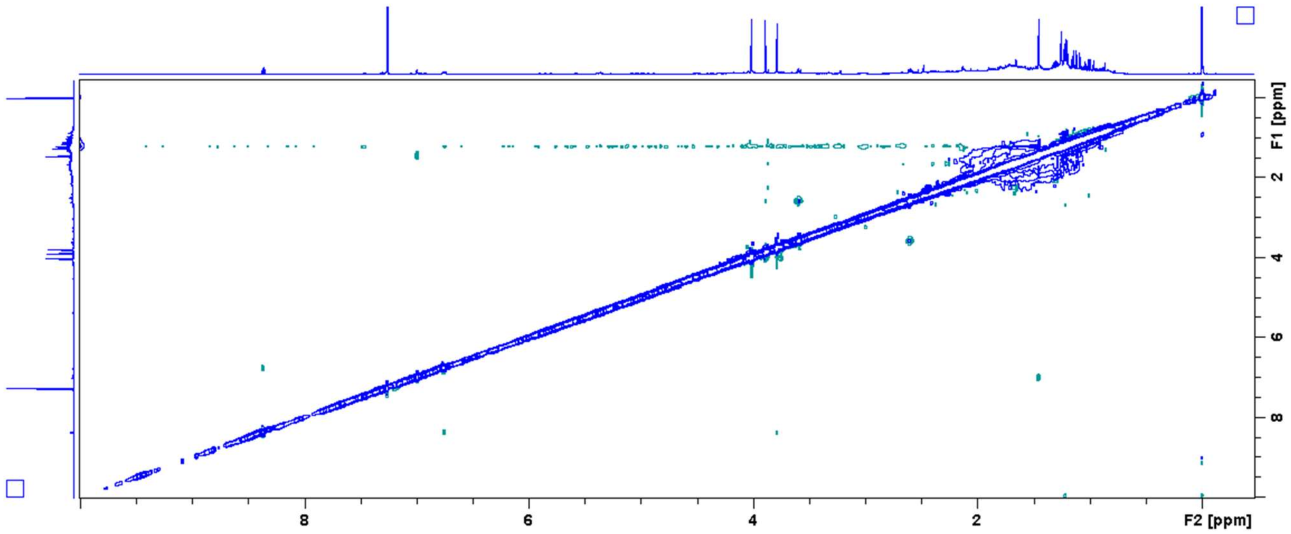

Figure S49. ^{1}H-^{1}H correlation map from NOESY NMR experiments of **9-hydroxyiguattescine** (**4**) in $CDCl_3$ at 500 (^{1}H) and 125 MHz (^{13}C).

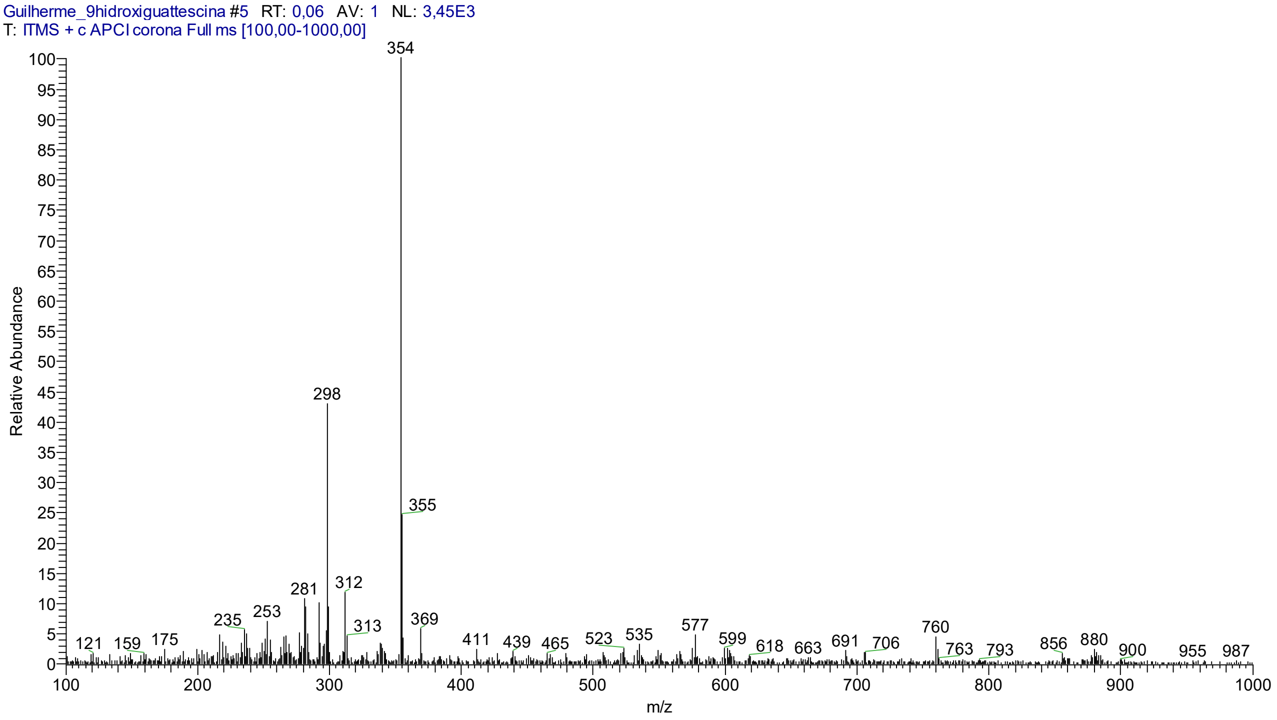

Figure S50. Full LR-APCI(+)-MS spectrum of **9-hydroxyiguattescine** (**4**) (*m/z* 354 $[M+H]^+$).

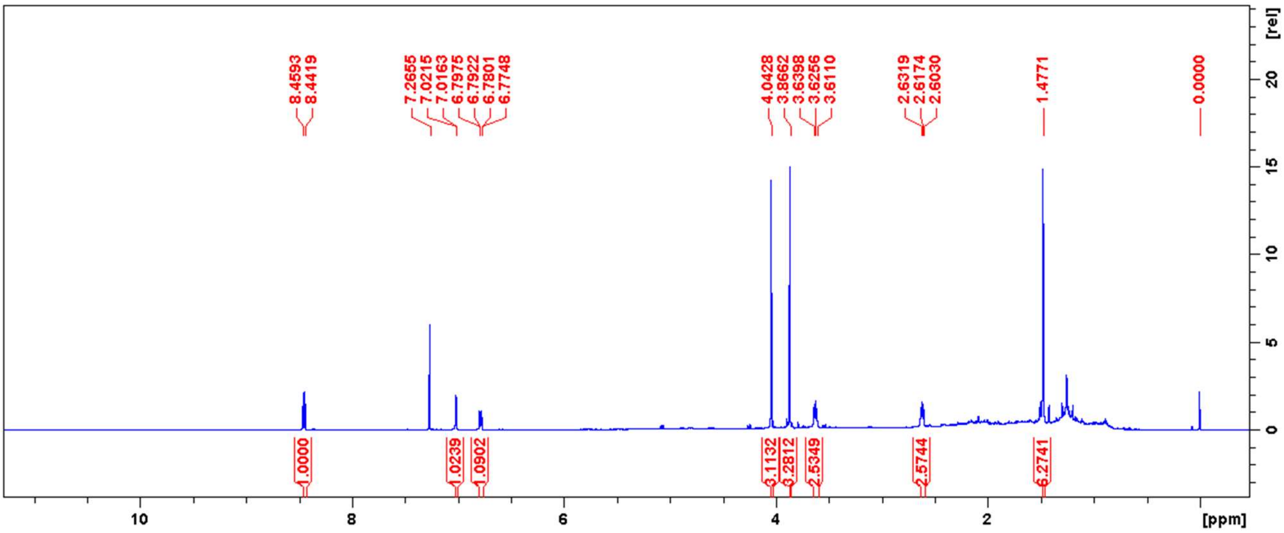

Figure S51. ^{1}H NMR spectrum of **dihydromelosmine** (**5**) in $CDCl_3$ at 500 MHz.

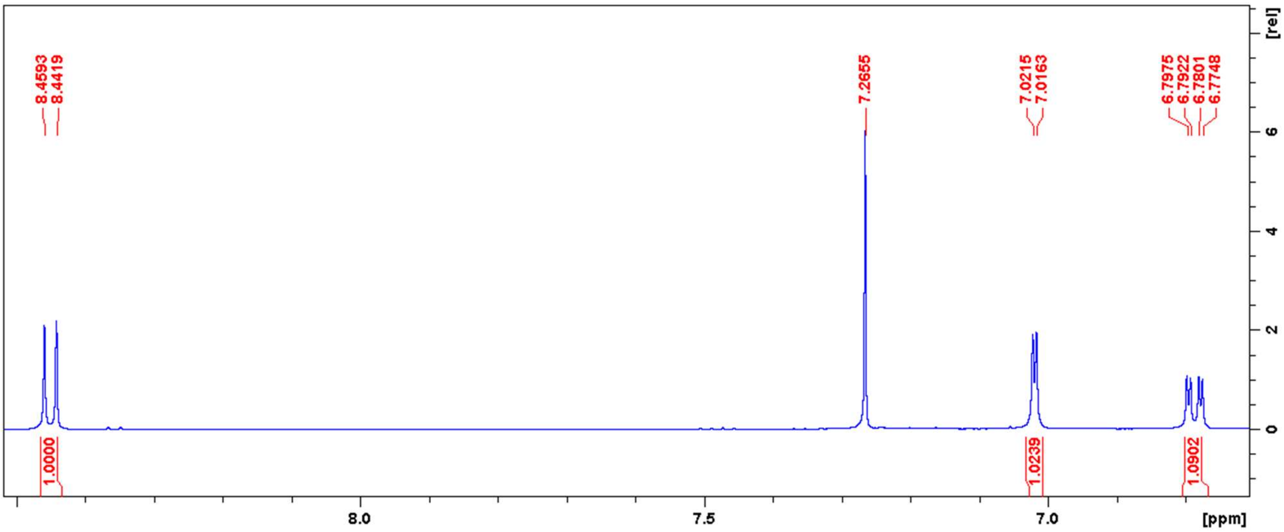

Figure S52. Expansion of aromatic hydrogen signals in the ^{1}H NMR spectrum of **dihydromelosmine** (**5**) in $CDCl_3$ at 500 MHz.

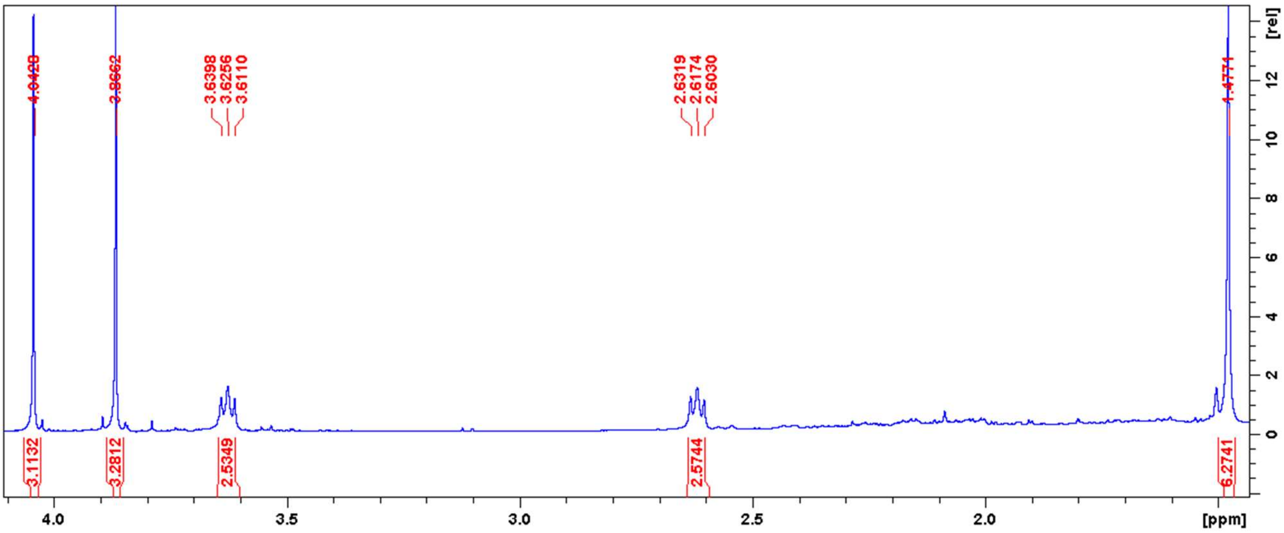

Figure S53. Expansion of the aliphatic hydrogen signals in the ^{1}H NMR spectrum of **dihydromelosmine** (**5**) in $CDCl_3$ at 500 MHz.

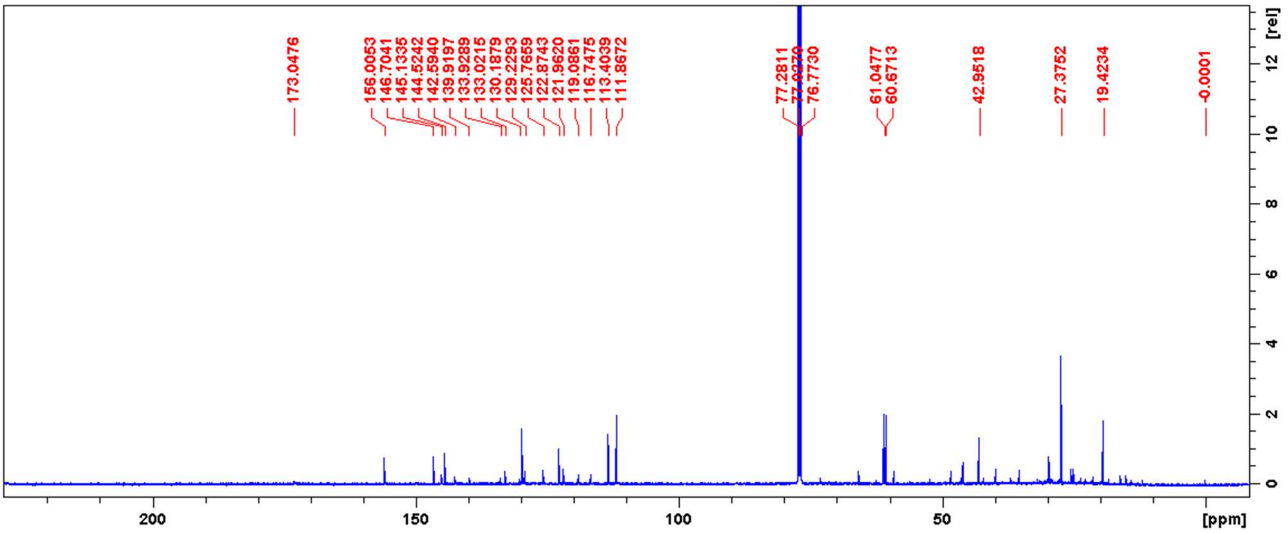

Figure S54. ^{13}C NMR spectrum of **dihydromelosmine** (5) in $CDCl_3$ at 125 MHz.

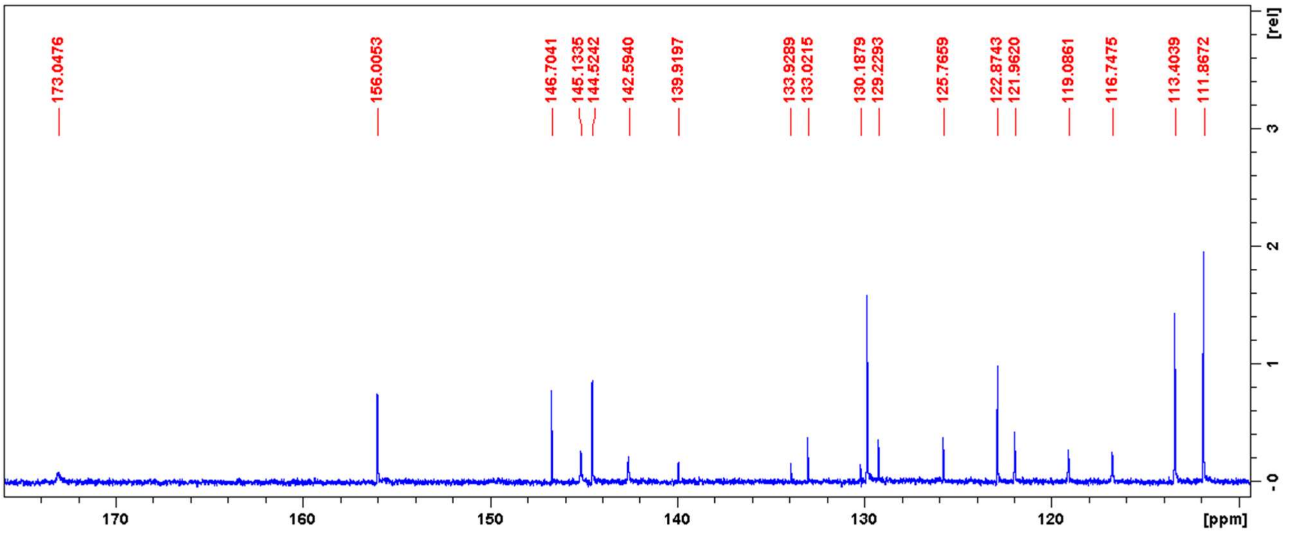

Figure S55. Expansion of aromatic carbon signals in the ^{13}C NMR spectrum of **dihydromelosmine** (**3**) in $CDCl_3$ at 125 MHz.

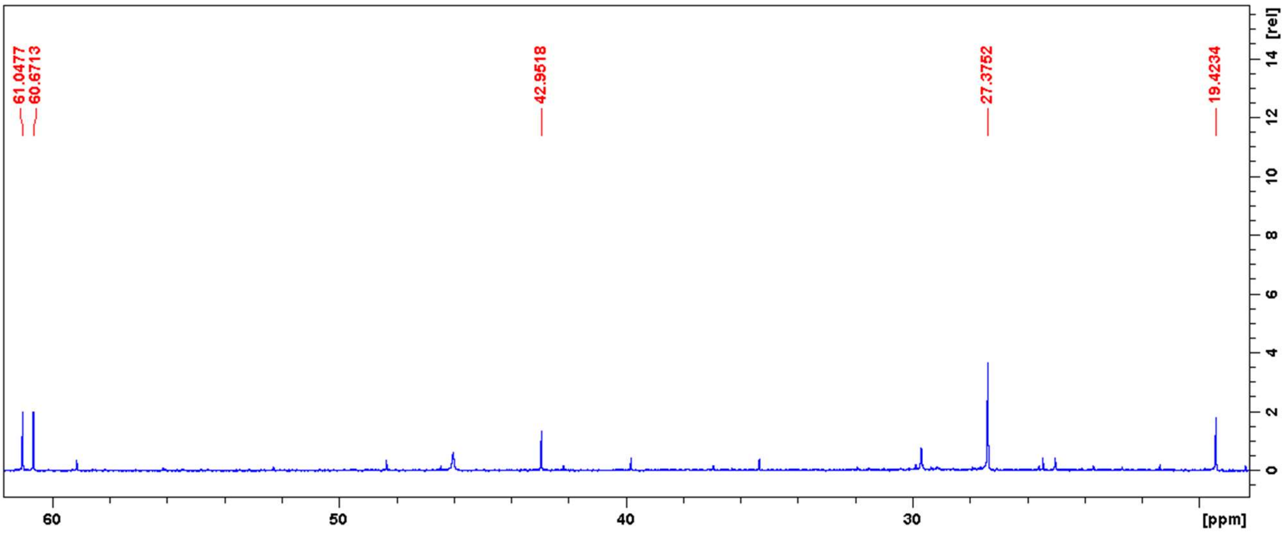

Figure S56. Expansion of the aliphatic carbon signals in the ^{13}C NMR spectrum of **dihydromelosmine** (**5**) in $CDCl_3$ at 125 MHz.

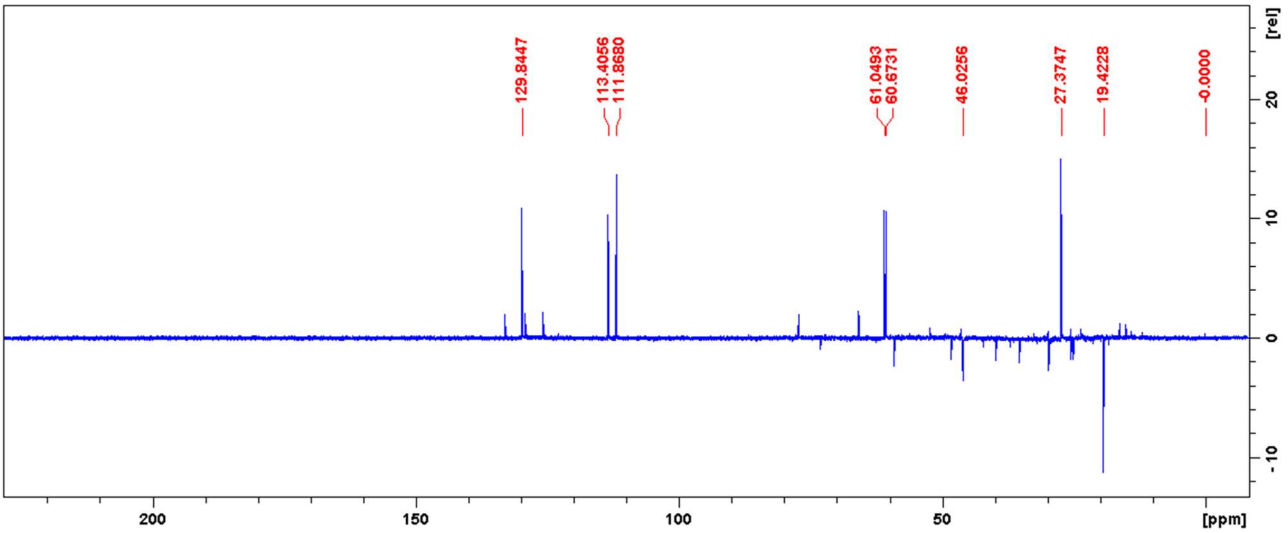

Figure S57. ^{13}C NMR DEPT 135 spectrum of **dihydromelosmine** (**5**) in $CDCl_3$ at 125 MHz.

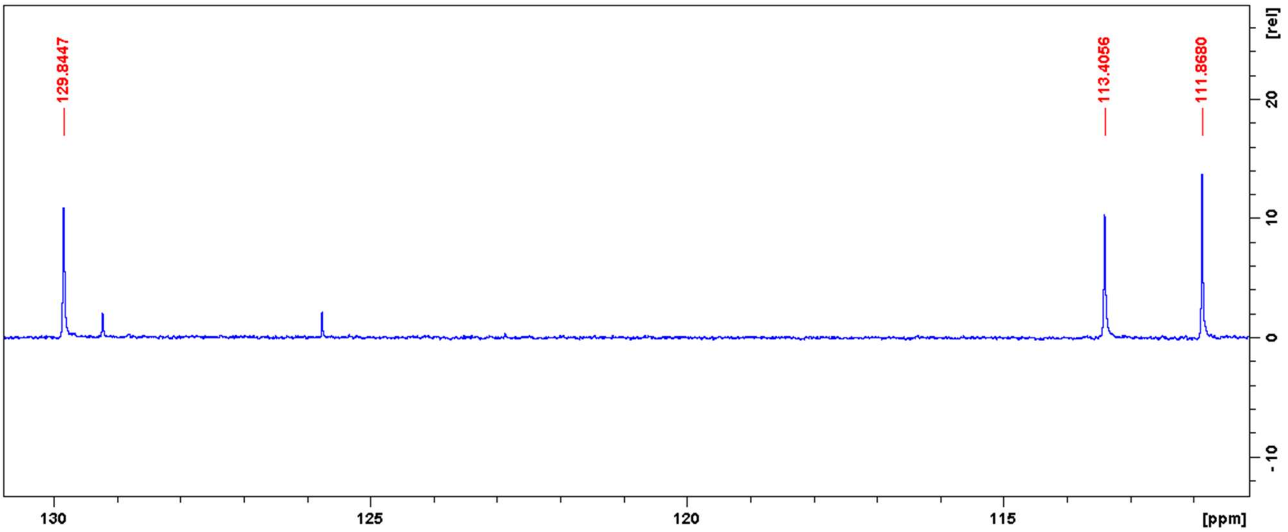

Figure S58. Expansion of aromatic carbon signals in the ^{13}C NMR DEPT 135 spectrum of **dihydromelosmine** (**5**) in $CDCl_3$ at 125 MHz.

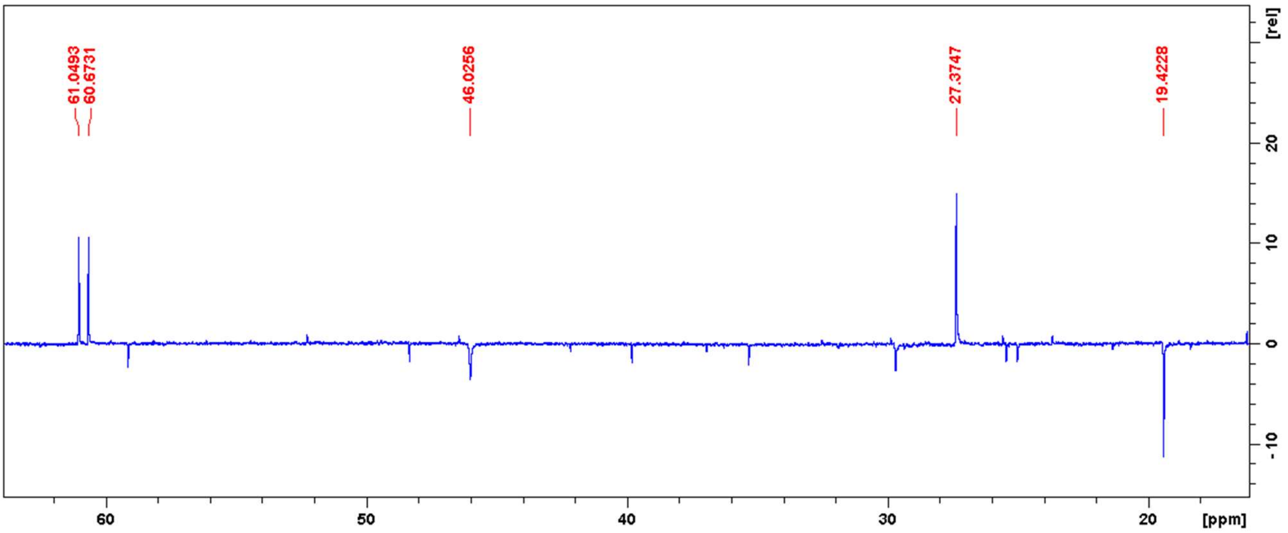

Figure S59. Expansion of the aliphatic carbon signals in the ^{13}C NMR DEPT 135 spectrum of **dihydromelosmine** (**5**) in $CDCl_3$ at 125 MHz.

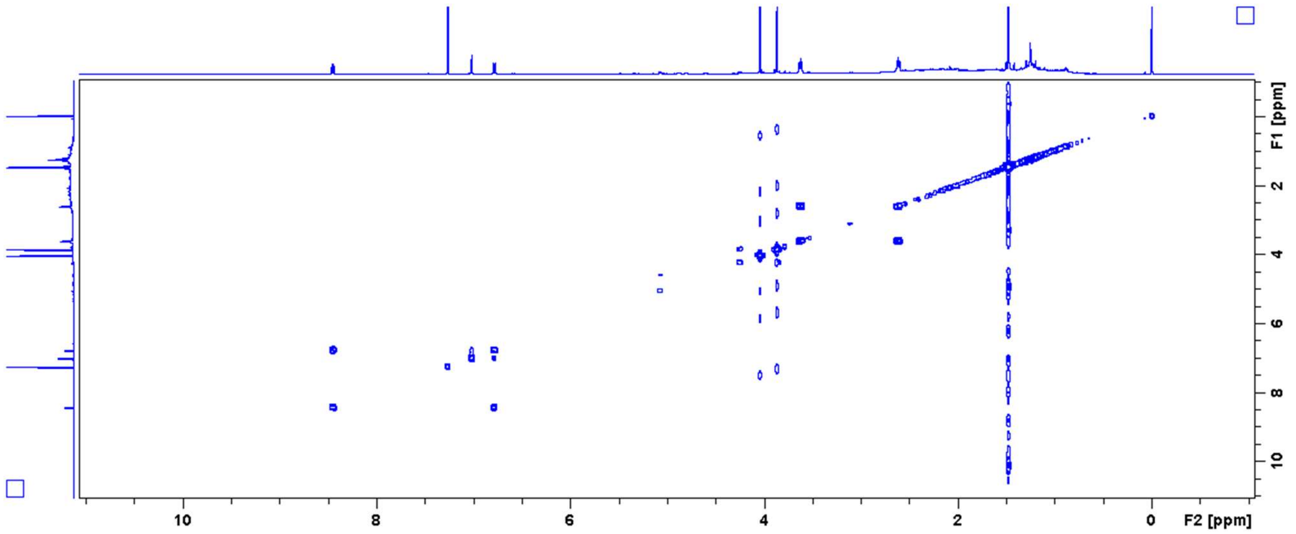

Figure S60. ^{1}H-^{1}H correlation map from the COSY NMR spectrum of **dihydromelosmine** (**5**) in $CDCl_3$ at 500 MHz.

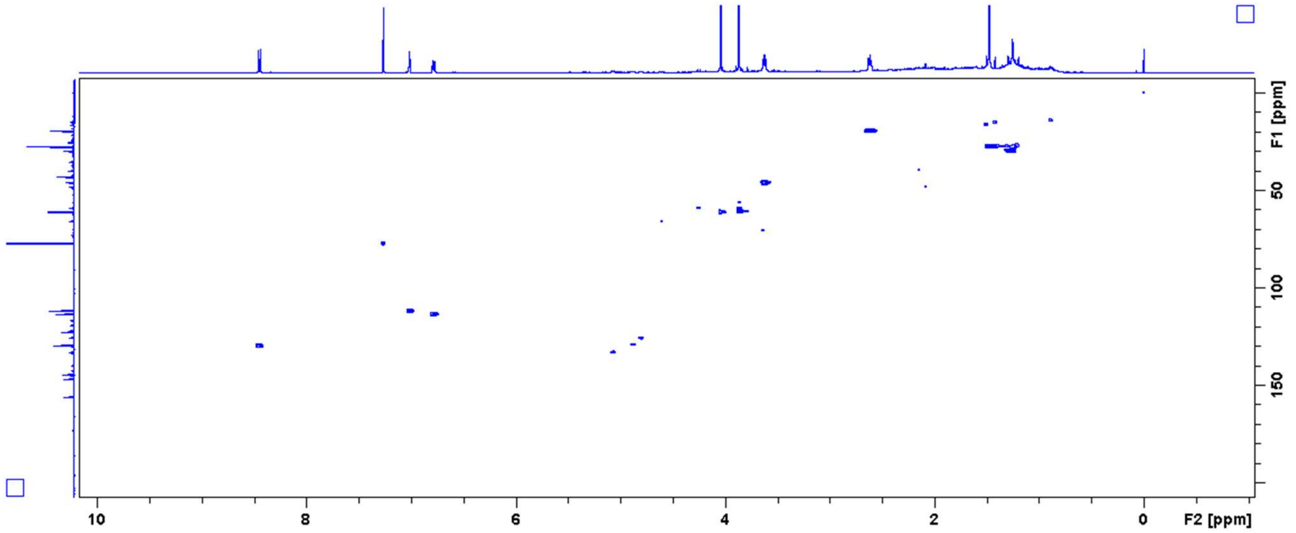

Figure S61. One-bond ^{1}H-^{13}C correlation map from the HSQC NMR spectrum of **dihydromelosmine** (**5**) in $CDCl_3$ at 500 (^{1}H) and 125 MHz (^{13}C).

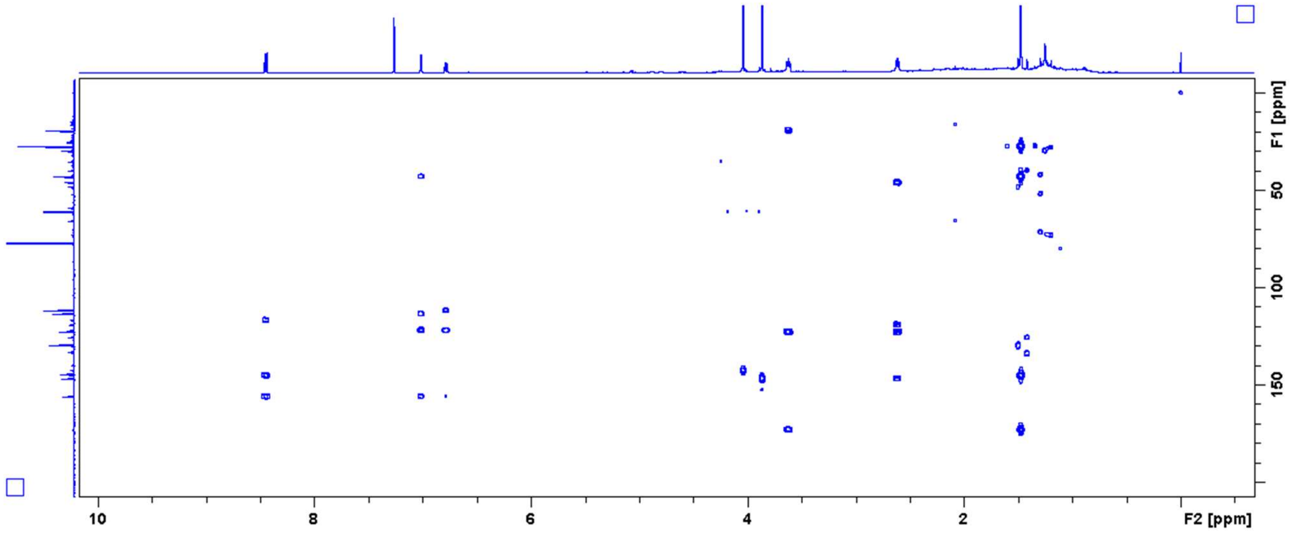

Figure S62. Long-range ^{1}H-^{13}C correlation map from the HMBC NMR spectrum of **dihydromelosmine** (**5**) in $CDCl_3$ at 500 (^{1}H) and 125 MHz (^{13}C).

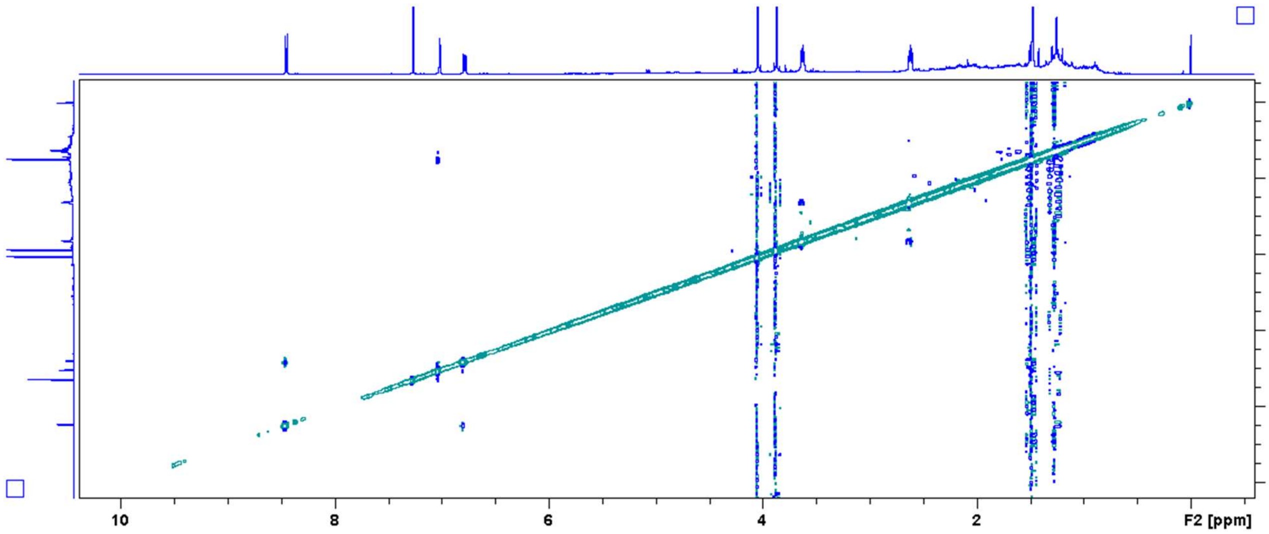

Figure S63. ^{1}H-^{1}H correlation map from NOESY NMR of **dihydromelosmine** (**5**) in $CDCl_3$ at 500 (^{1}H) and 125 MHz (^{13}C).

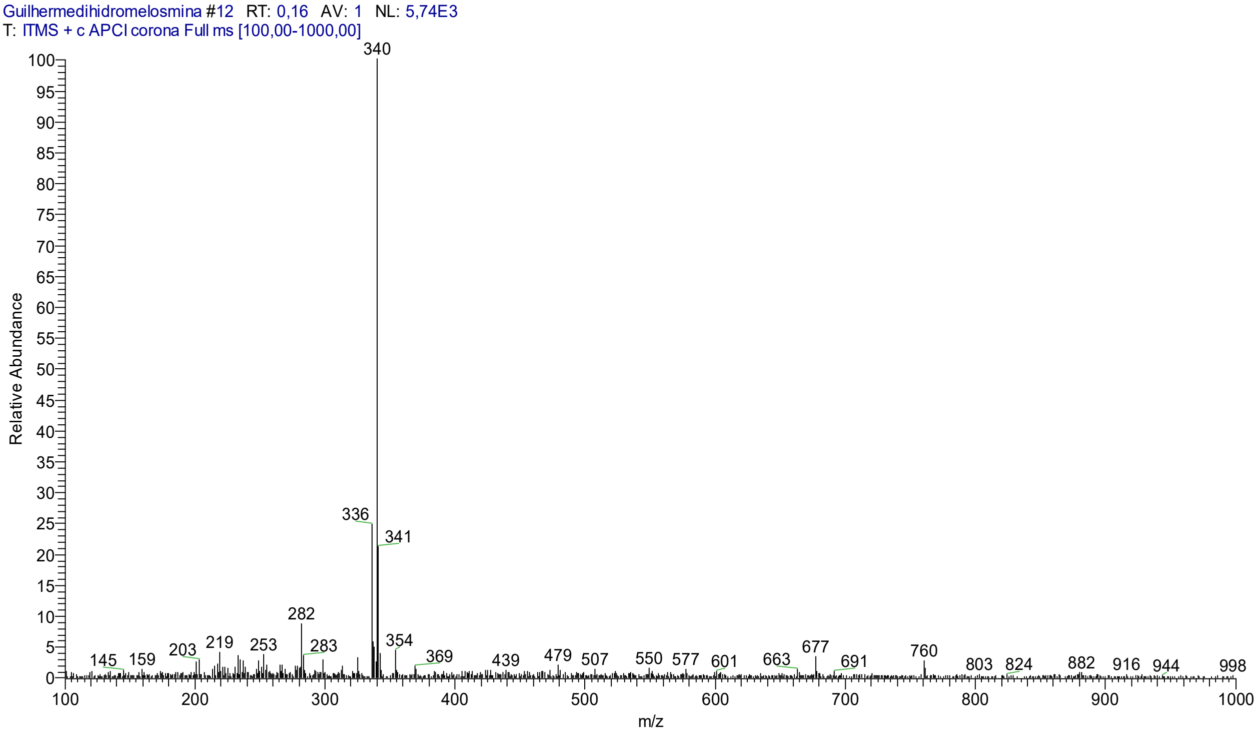

Figure S64. Full LR-APCI(+)-MS spectrum of **dihydromelosmine** (**5**) (*m/z* 340 $[M+H]^{+}$).

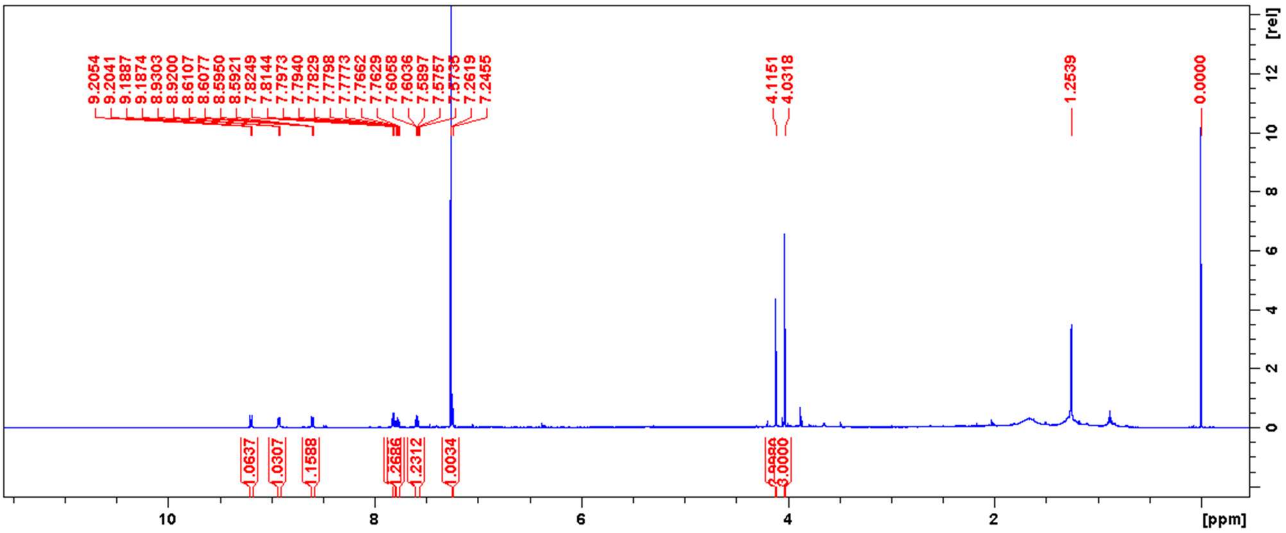

Figure S65. ^{1}H NMR spectrum of **lysicamine** (**6**) in $CDCl_3$ at 500 MHz.

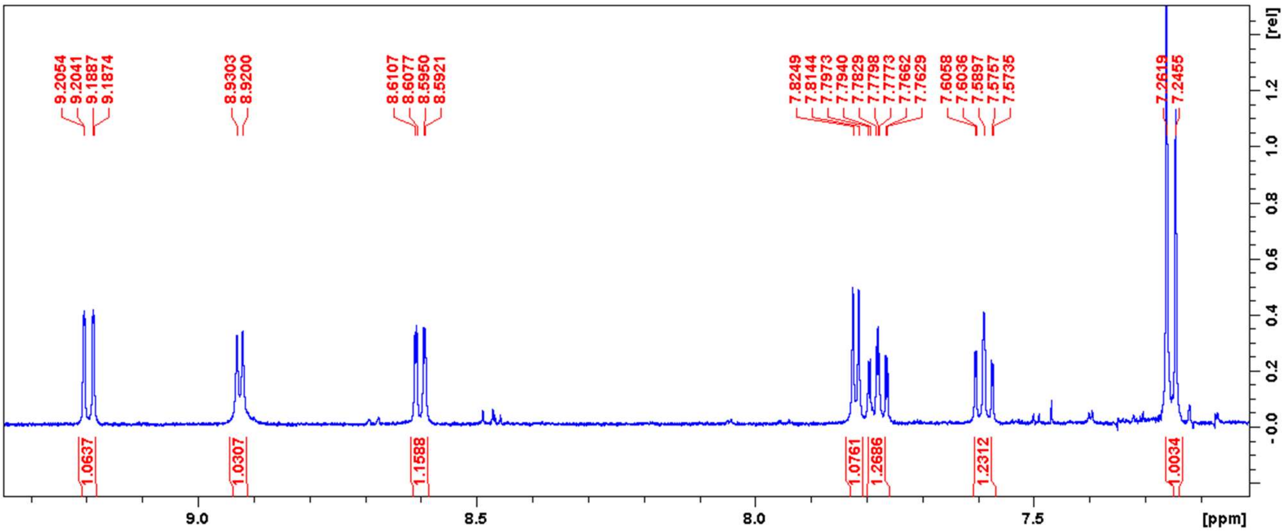

Figure S66. Expansion of aromatic hydrogen signals in the ^{1}H NMR spectrum of **lysicamine** (**6**) in $CDCl_3$ at 500 MHz.

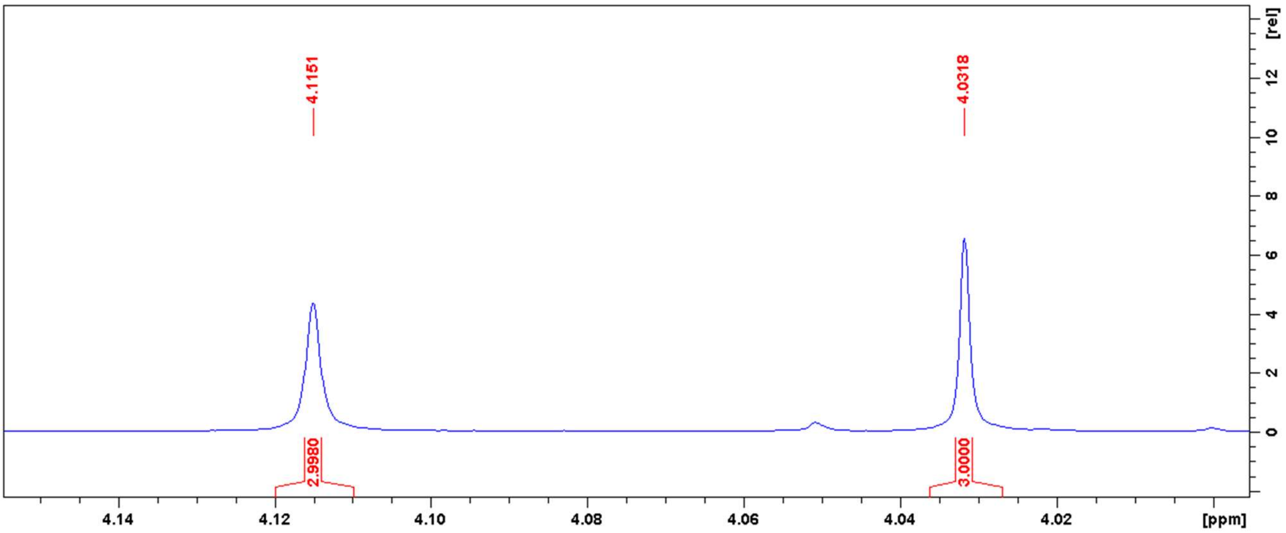

Figure S67. Expansion of the aliphatic hydrogen signals in the ^{1}H NMR spectrum of **lysicamine** (**6**) in $CDCl_3$ at 500 MHz.

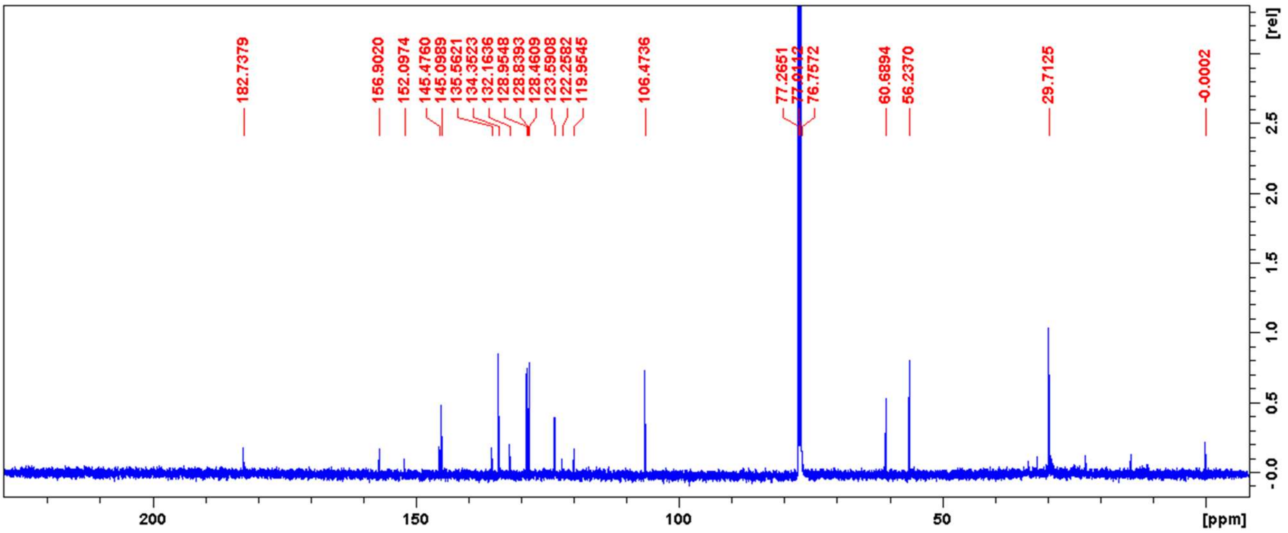

Figure S68. ^{13}C NMR spectrum of **lysicamine** (**6**) in $CDCl_3$ at 125 MHz.

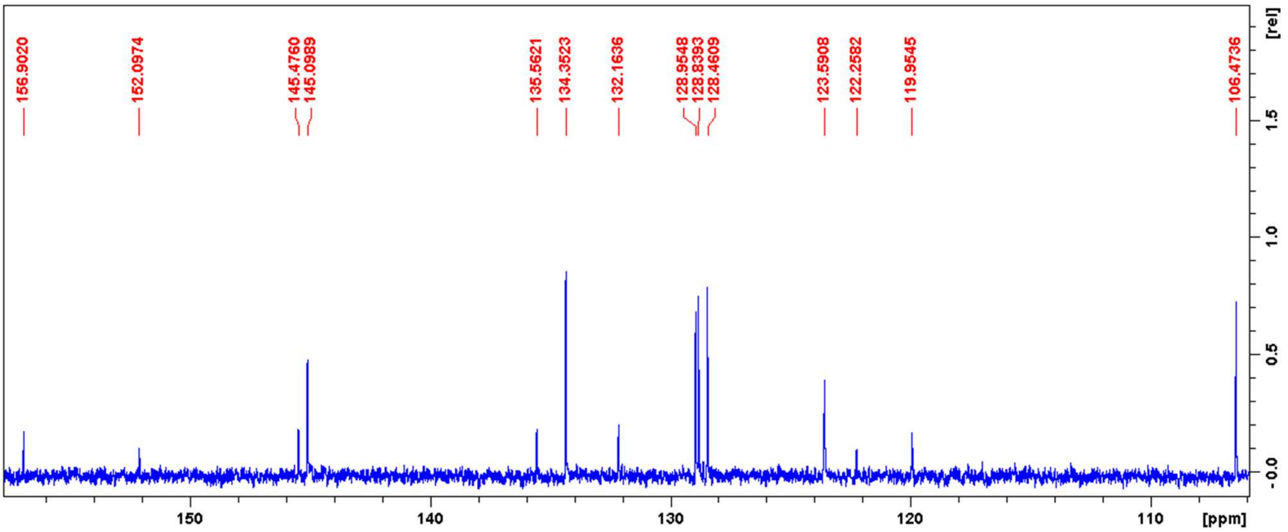

Figure S69. Expansion of aromatic carbon signals in the ^{13}C NMR spectrum of **lysicamine** (**6**) in $CDCl_3$ at 125 MHz.

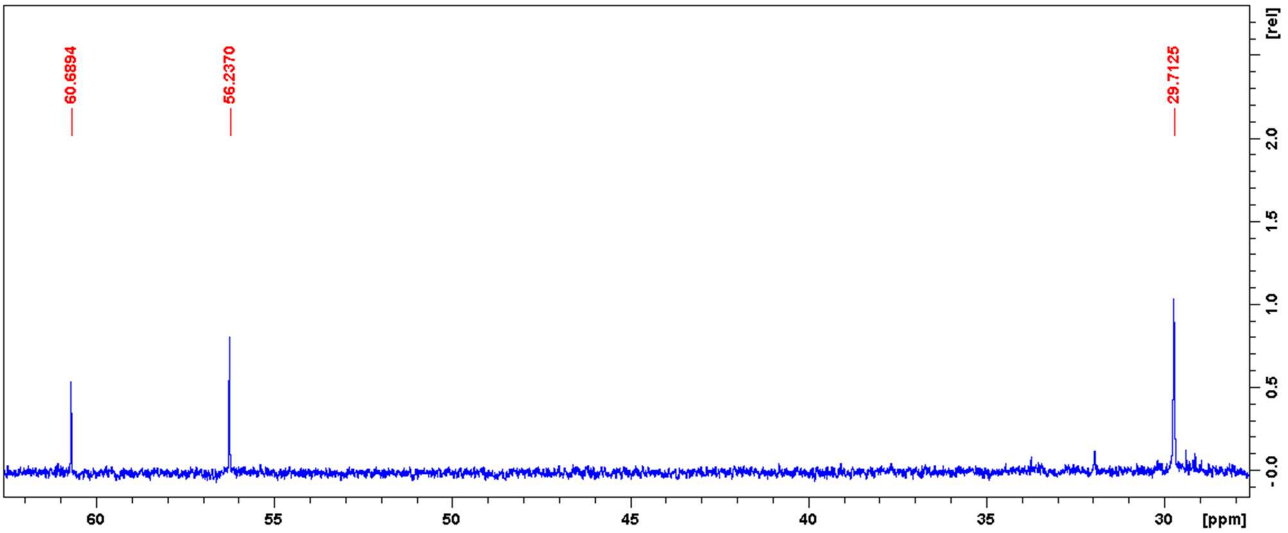

Figure S70. Expansion of the aliphatic carbon signals in the ^{13}C NMR spectrum of **lysicamine** (**6**) in $CDCl_3$ at 125 MHz.

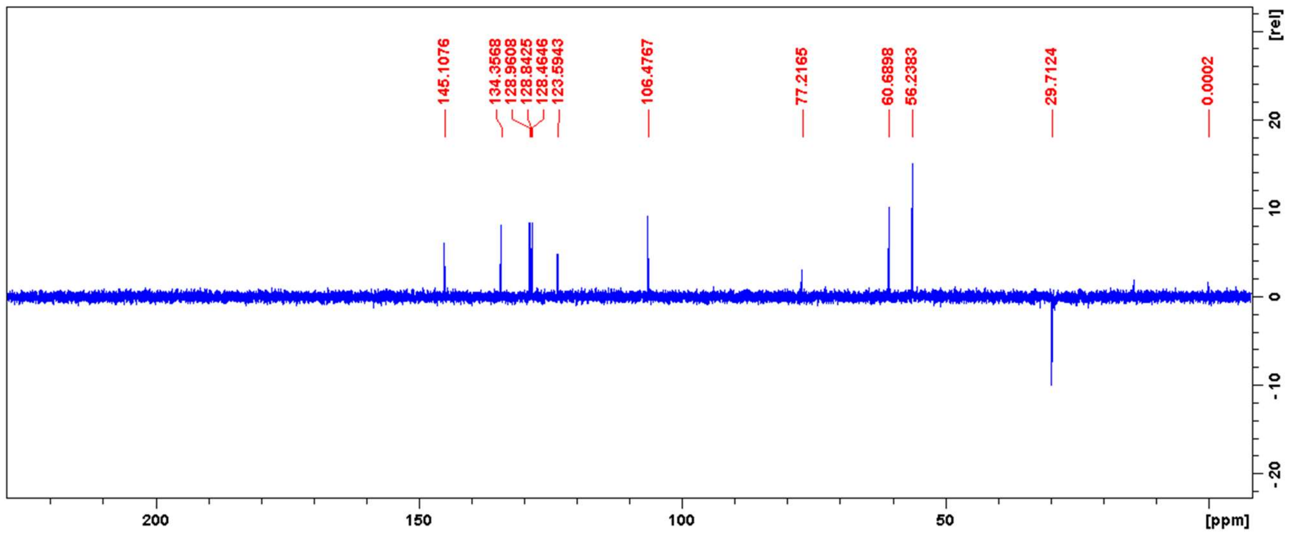

Figure S71. ^{13}C NMR DEPT 135 spectrum of **lysicamine** (**6**) in $CDCl_3$ at 125 MHz.

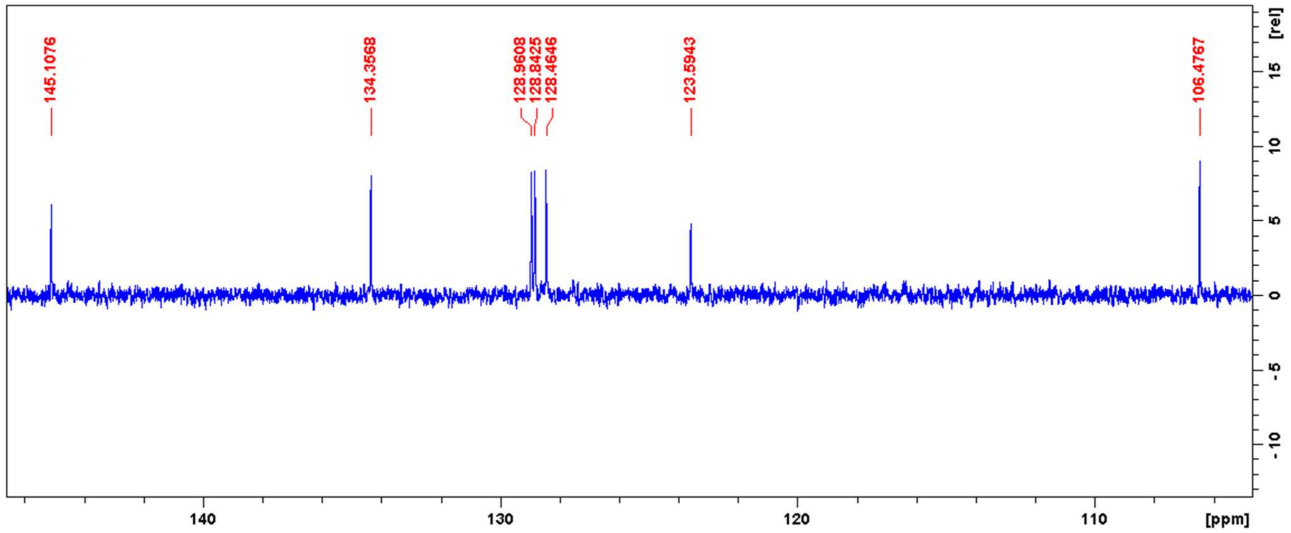

Figure S72. Expansion of aromatic carbon signals in the ^{13}C NMR DEPT 135 spectrum of **lysicamine** (**6**) in $CDCl_3$ at 125 MHz.

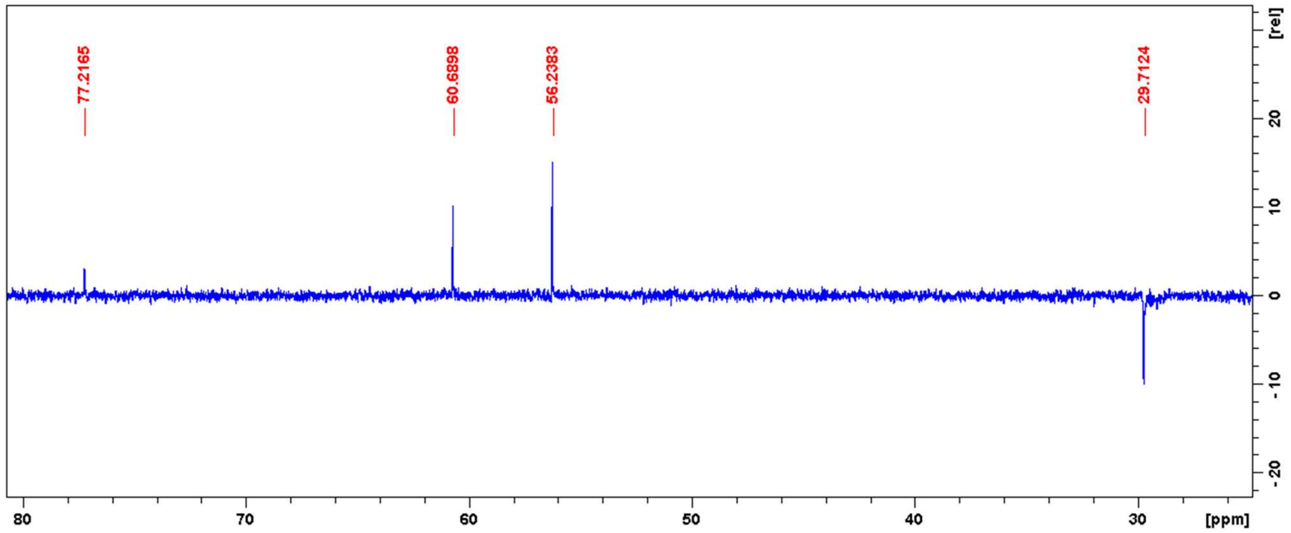

Figure S73. Expansion of the aliphatic carbon signals in the ^{13}C NMR DEPT 135 spectrum of **lysicamine** (**6**) in $CDCl_3$ at 125 MHz.

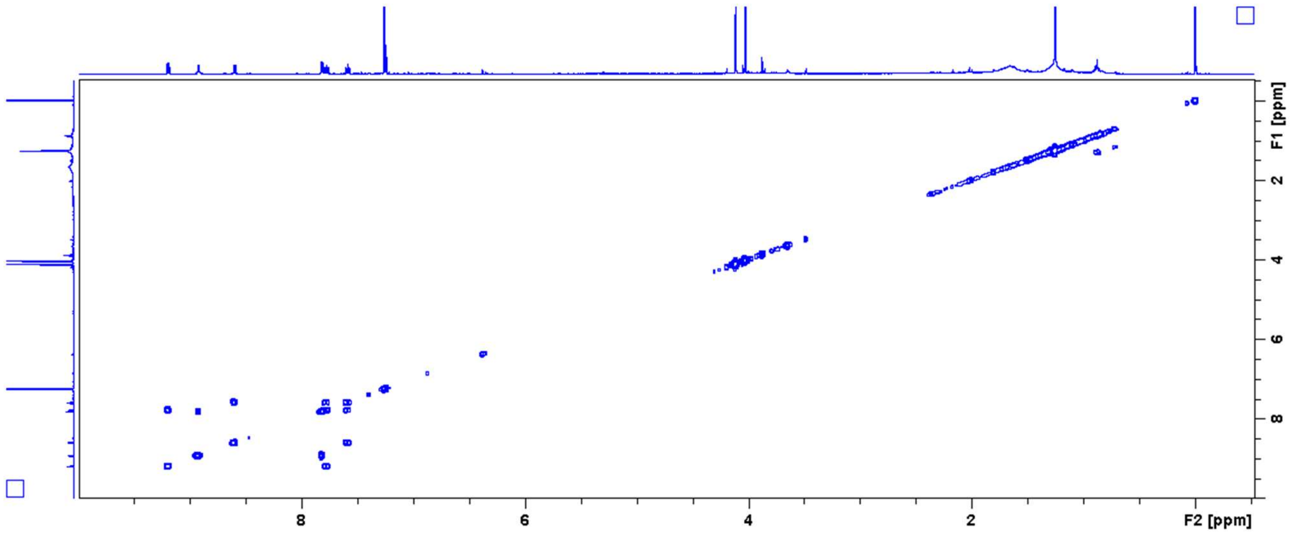

Figure S74. ^1H-^1H correlation map from the COSY NMR spectrum of **lysicamine** (**6**) in $CDCl_3$ at 500 MHz.

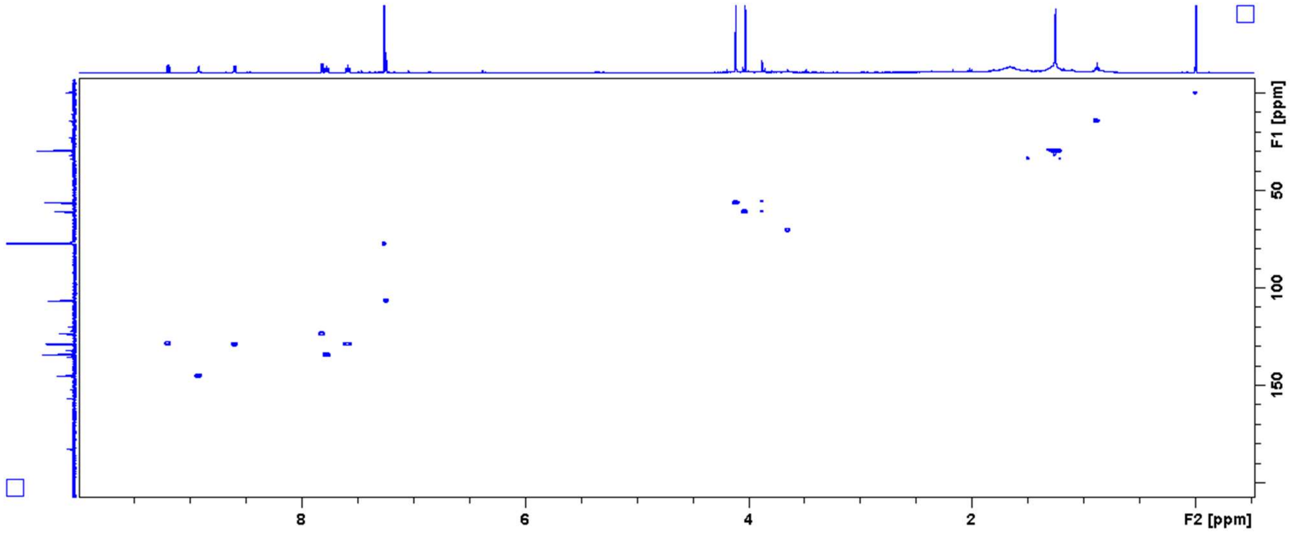

Figure S75. One-bond ^1H-^{13}C correlation map from the HSQC NMR spectrum of **lysicamine** (**6**) in $CDCl_3$ at 500 (^1H) and 125 MHz (^{13}C).

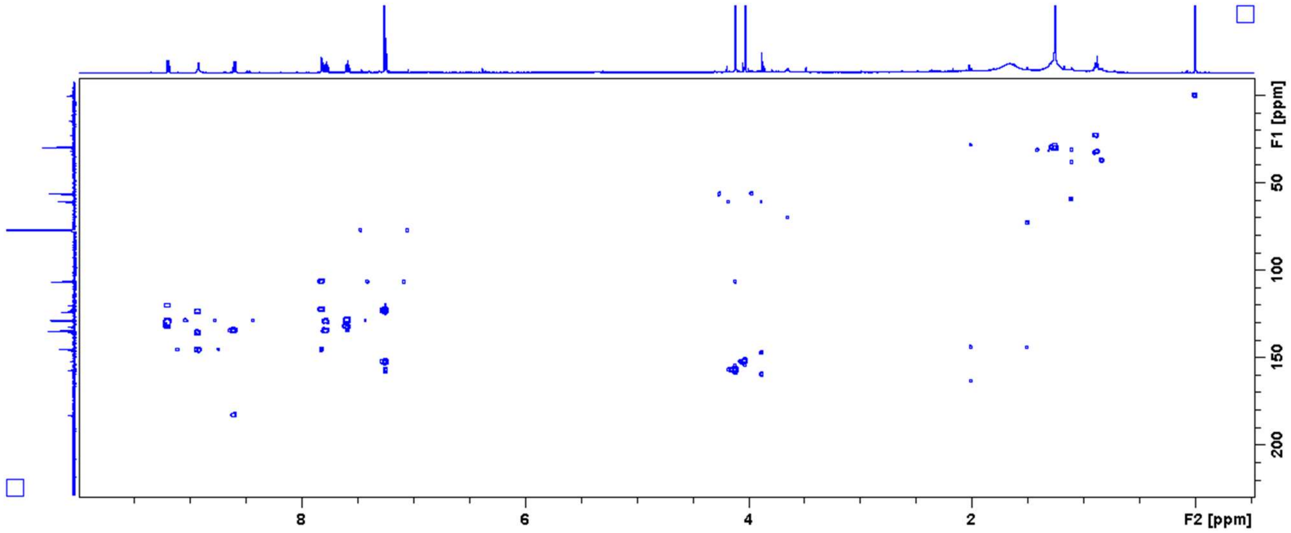

Figure S76. Long-range ^1H-^{13}C correlation map from the HMBC NMR spectrum of **lysicamine** (**6**) in $CDCl_3$ at 500 (^1H) and 125 MHz (^{13}C).

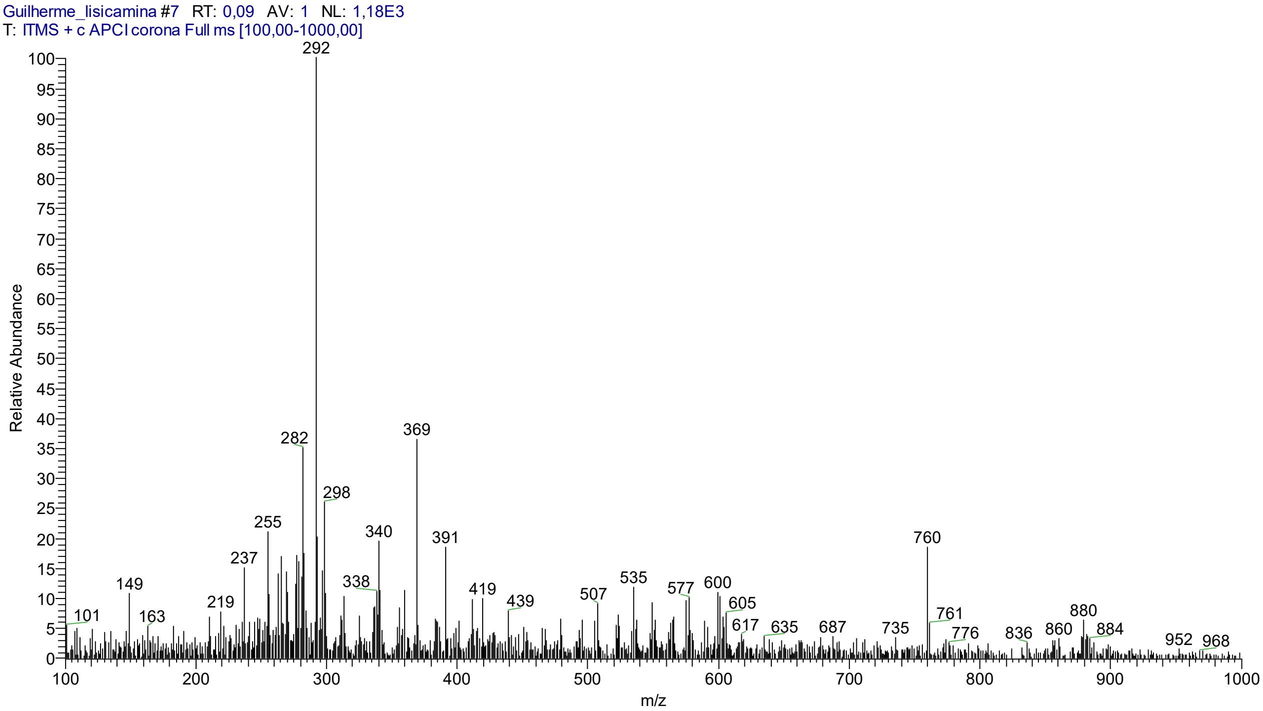

Figure S77. Full LR-APCI(+)-MS spectrum of **lysicamine** (**6**) (*m/z* 340 $[M+H]^+$).

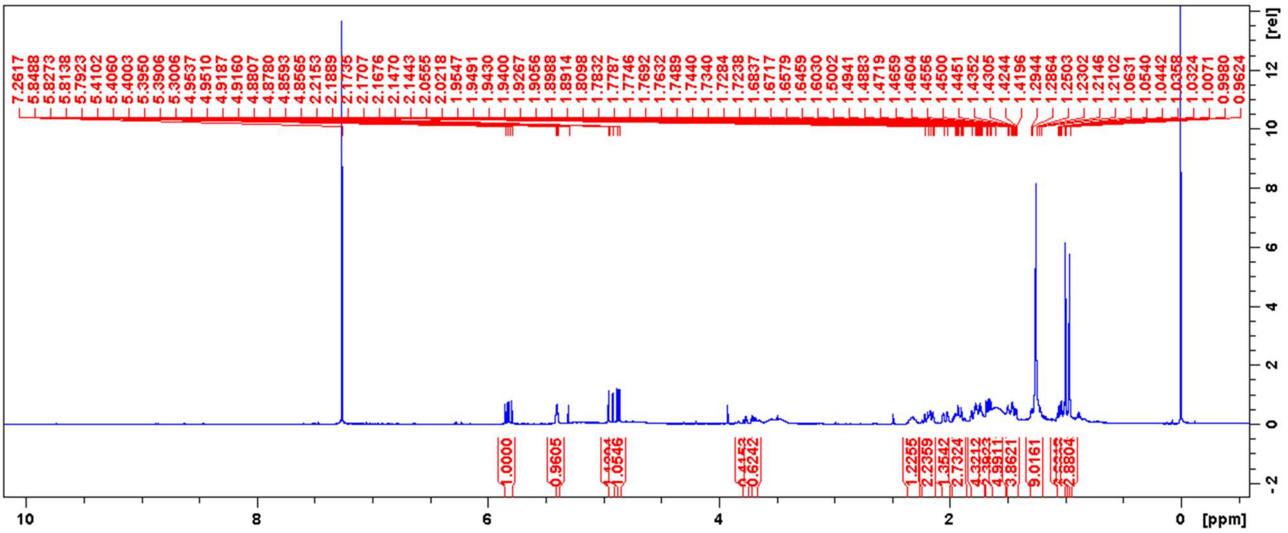

Figure S78. ^{1}H NMR spectrum of **acanthoic acid** (7) in $CDCl_3$ at 500 MHz.

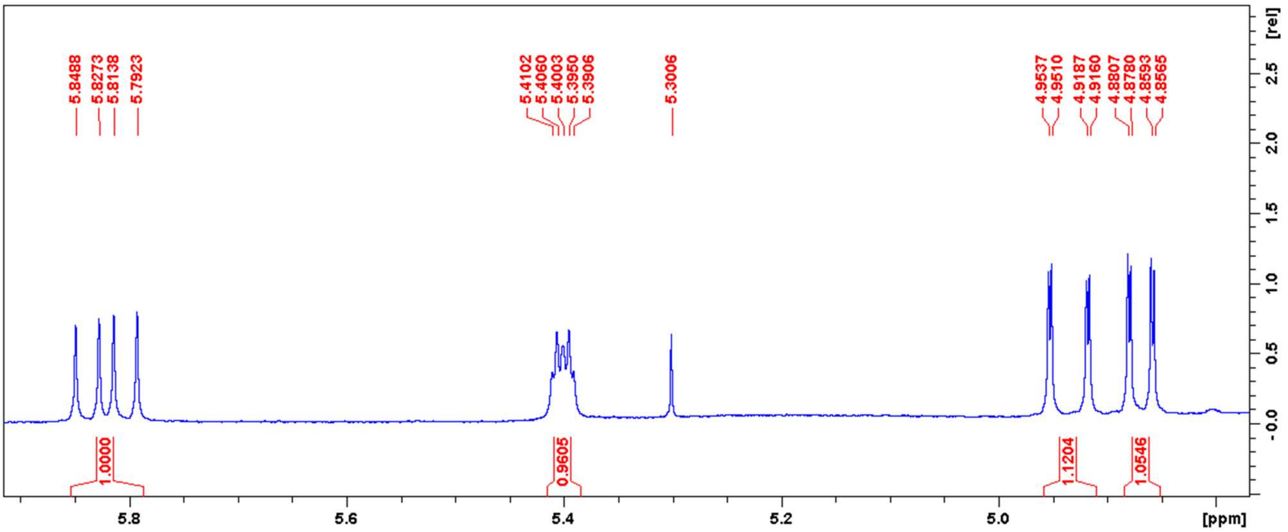

Figure S79. Expansion of the vinyl and olefinic hydrogen signals in the ^{1}H NMR spectrum of **acanthoic acid** (7) in $CDCl_3$ at 500 MHz.

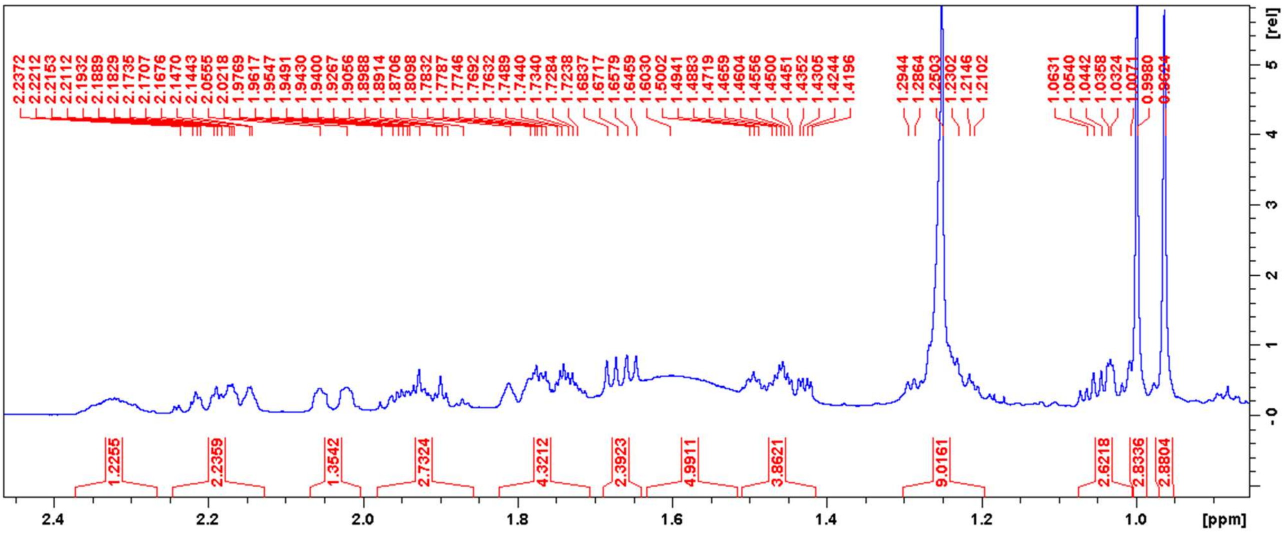

Figure S80. Expansion of the aliphatic hydrogen signals in the ^{1}H NMR spectrum of **acanthoic acid** (7) in $CDCl_3$ at 500 MHz.

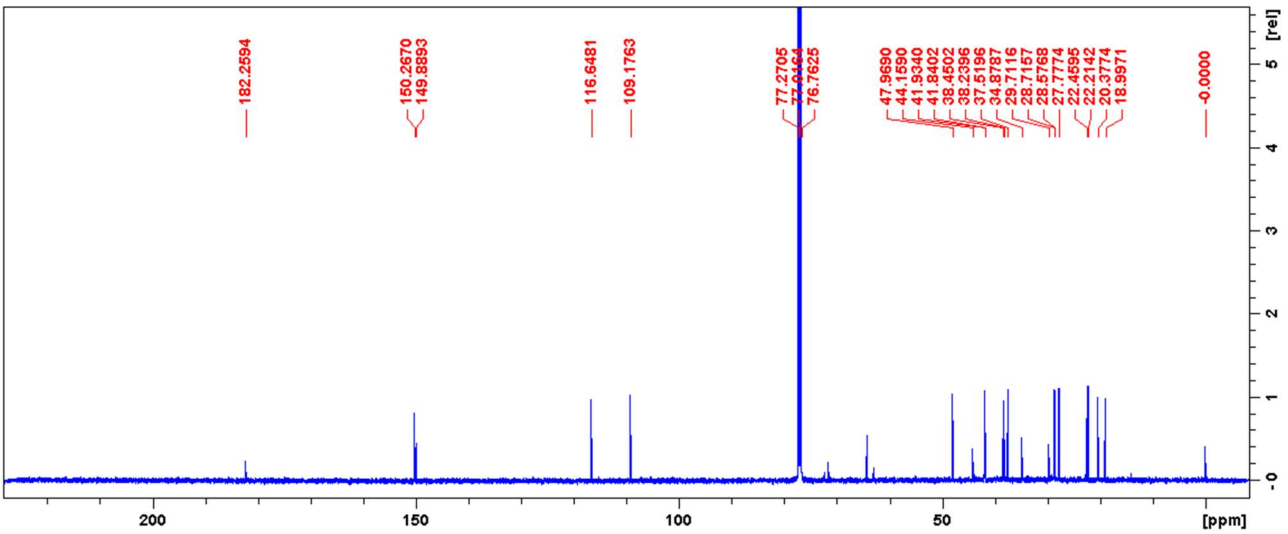

Figure S81. ^{13}C NMR spectrum of **acanthoic acid** (**7**) in $CDCl_3$ at 125 MHz.

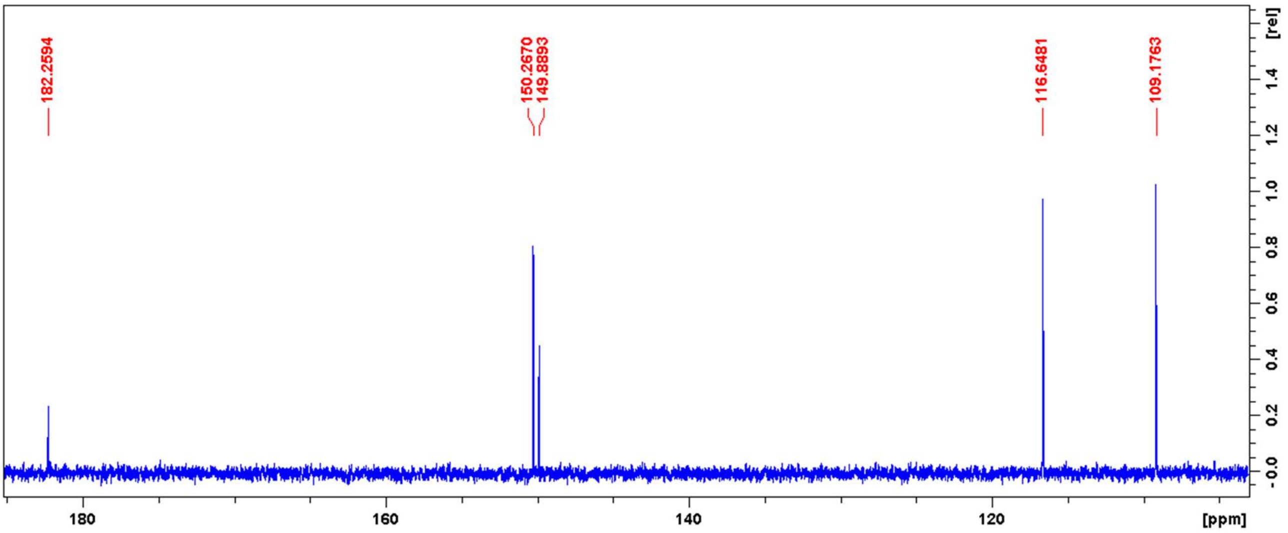

Figure S82. Expansion of the *sp*^{2} carbon signals in the ^{13}C NMR spectrum of **acanthoic acid** (**7**) in $CDCl_3$ at 125 MHz.

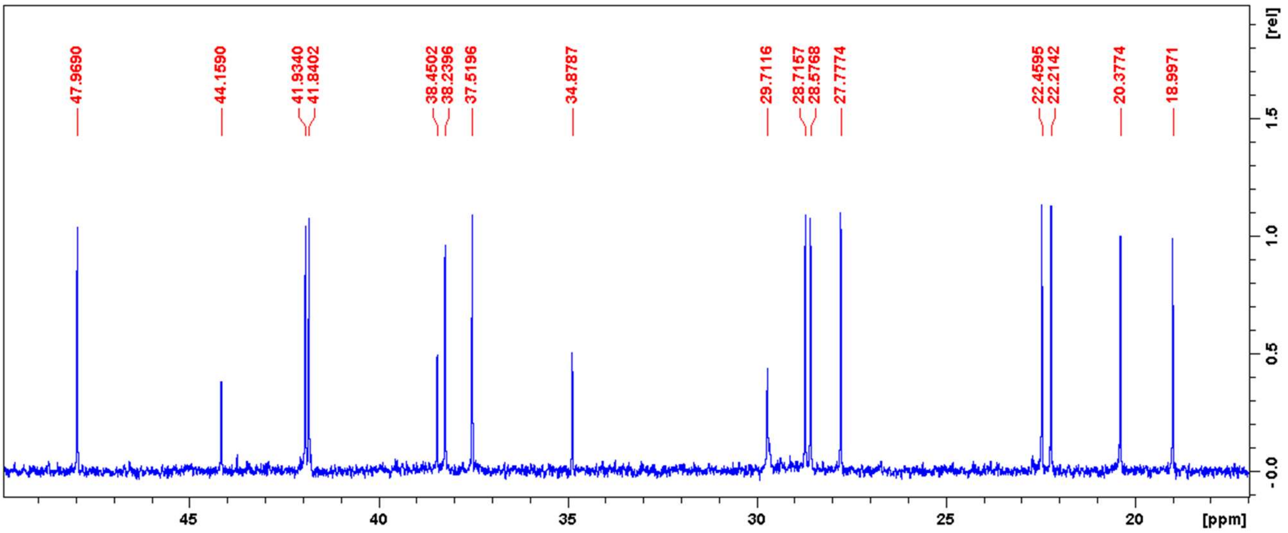

Figure S83. Expansion of the aliphatic carbon signals in the ^{13}C NMR spectrum of **acanthoic acid** (**7**) in $CDCl_3$ at 125 MHz.

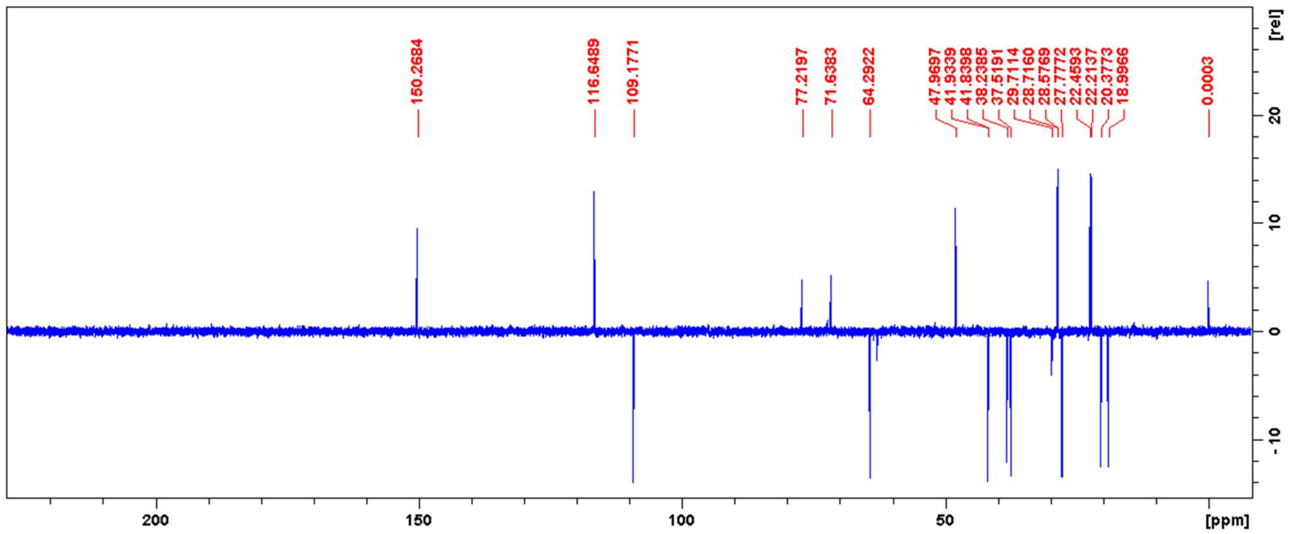

Figure S84. ^{13}C NMR DEPT 135 spectrum of **acanthoic acid** (**7**) in $CDCl_3$ at 125 MHz.

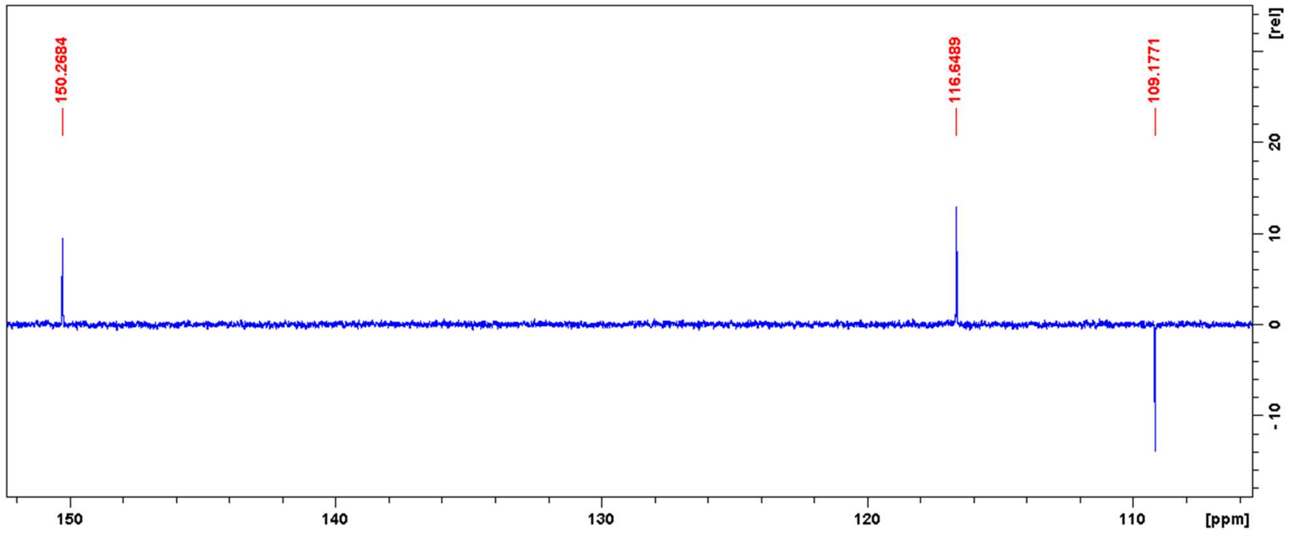

Figure S85. Expansion of the *sp*^{2} carbon signals in the ^{13}C NMR DEPT 135 spectrum of **acanthoic acid** (**7**) in $CDCl_3$ at 125 MHz.

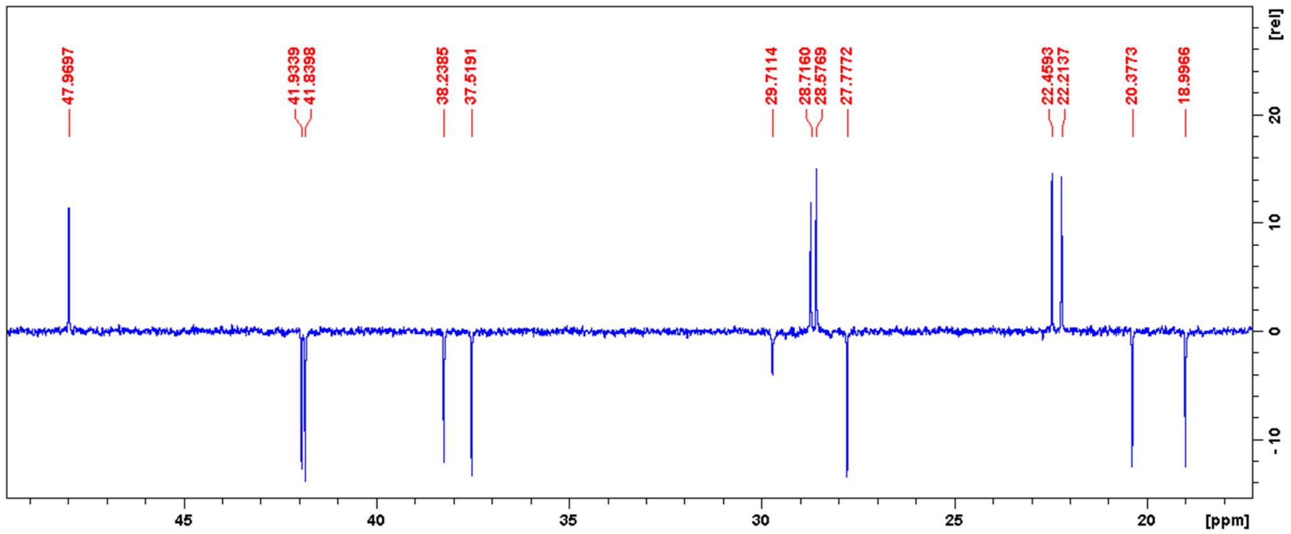

Figure S86. Expansion of the aliphatic carbon signals in the ^{13}C NMR DEPT 135 spectrum of **acanthoic acid** (**7**) in $CDCl_3$ at 125 MHz.

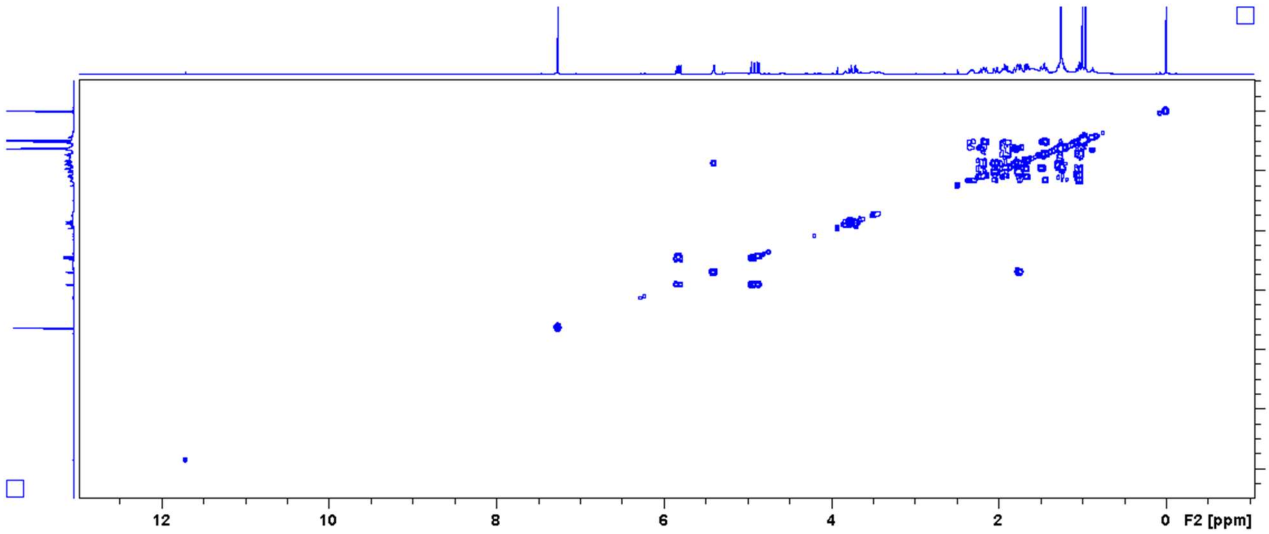

Figure S87. ^1H-^1H correlation map from the COSY NMR spectrum of **acanthoic acid** (**7**) in $CDCl_3$ at 500 MHz.

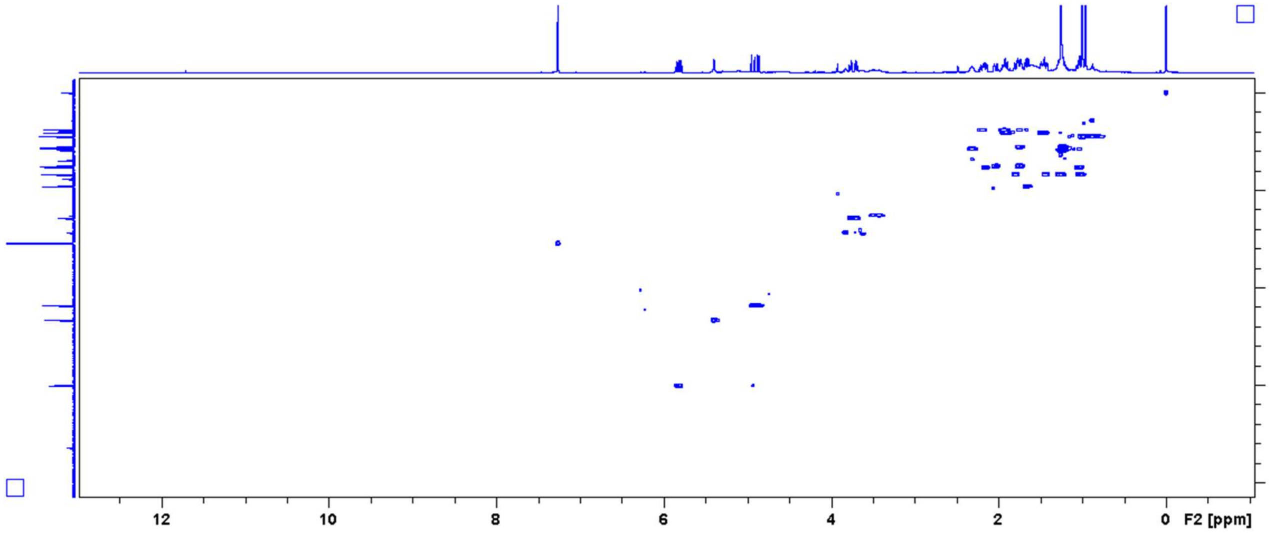

Figure S88. One-bond ^1H-^{13}C correlation map from the HSQC NMR spectrum of **acanthoic acid** (**7**) in $CDCl_3$ at 500 (^1H) and 125 MHz (^{13}C).

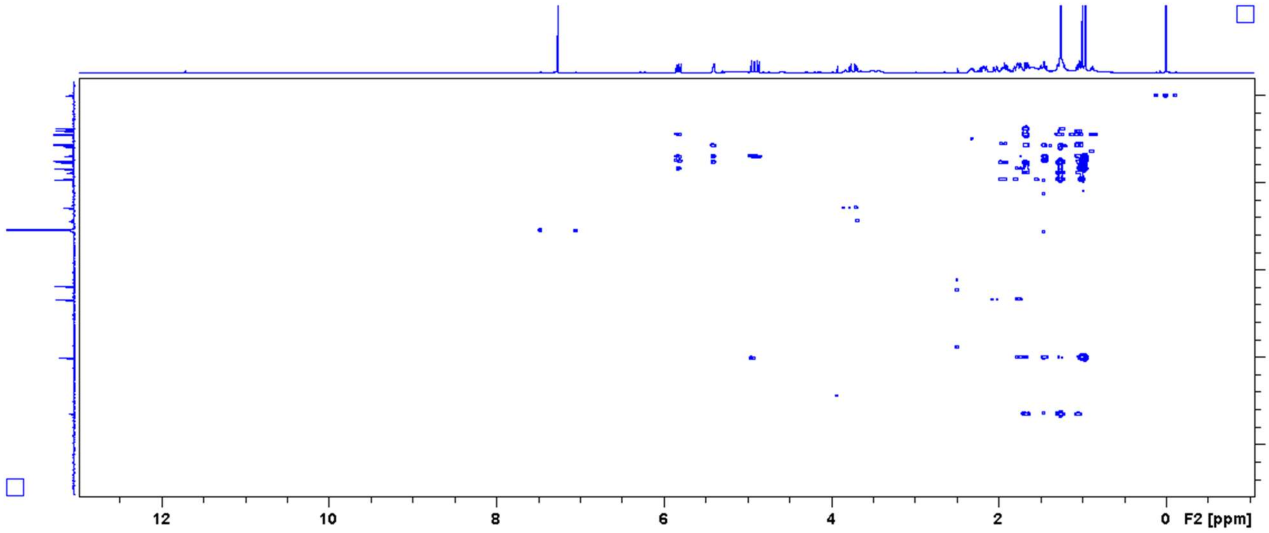

Figure S89. Long-range ^1H-^{13}C correlation map from the HMBC NMR spectrum of **acanthoic acid** (**7**) in $CDCl_3$ at 500 (^1H) and 125 MHz (^{13}C).

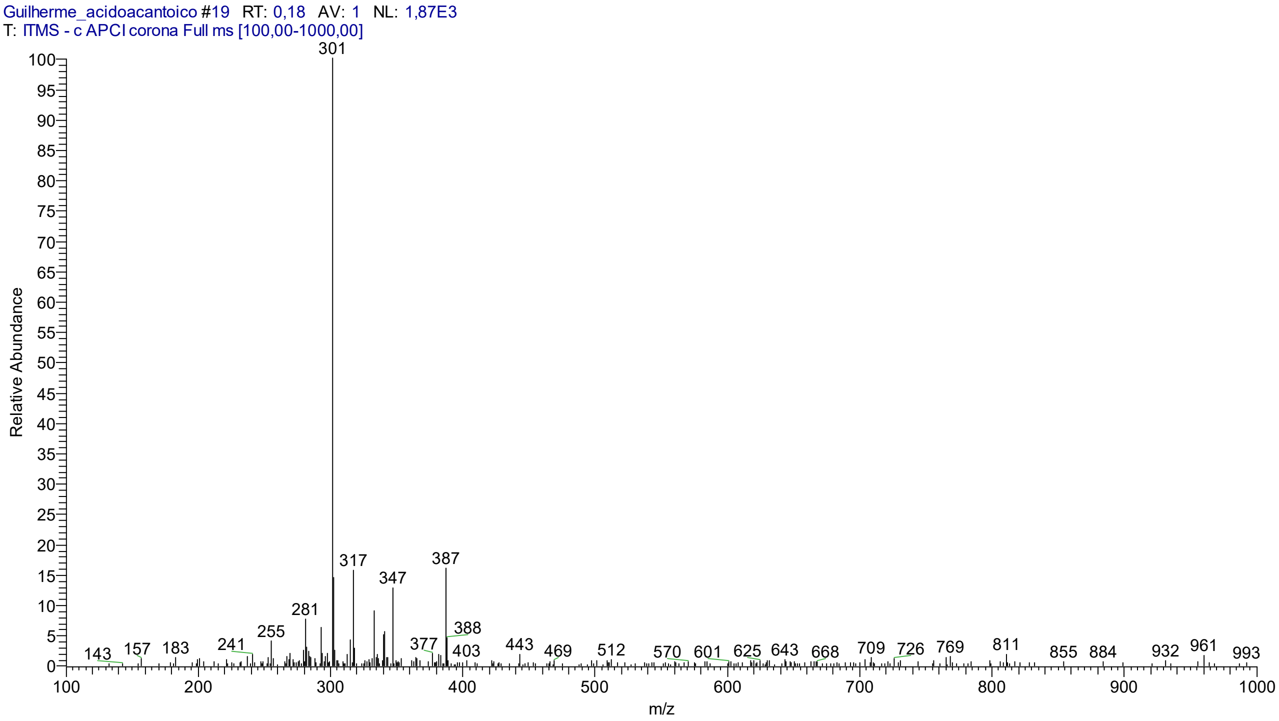

Figure S90. Full LR-APCI(−)-MS spectrum of **acanthoic acid** (**7**) (*m/z* 301 [M–H]⁻).

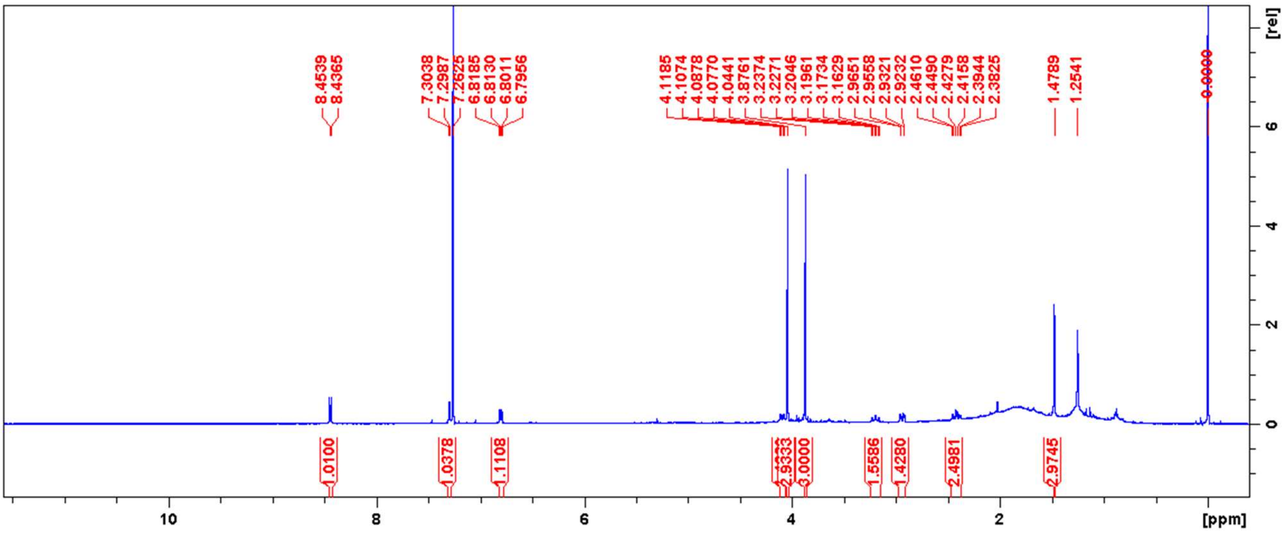

Figure S91. ^{1}H NMR spectrum of **guattouregidine** (**8**) in $CDCl_3$ at 500 MHz.

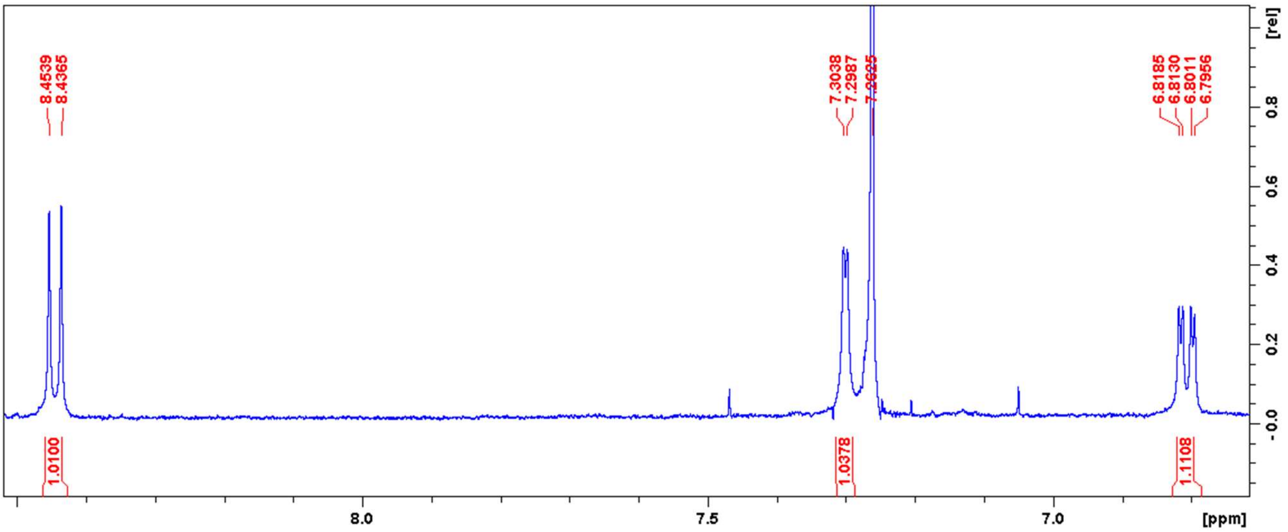

Figure S92. Expansion of aromatic hydrogen signals in the ^{1}H NMR spectrum of **guattouregidine** (**8**) in $CDCl_3$ at 500 MHz.

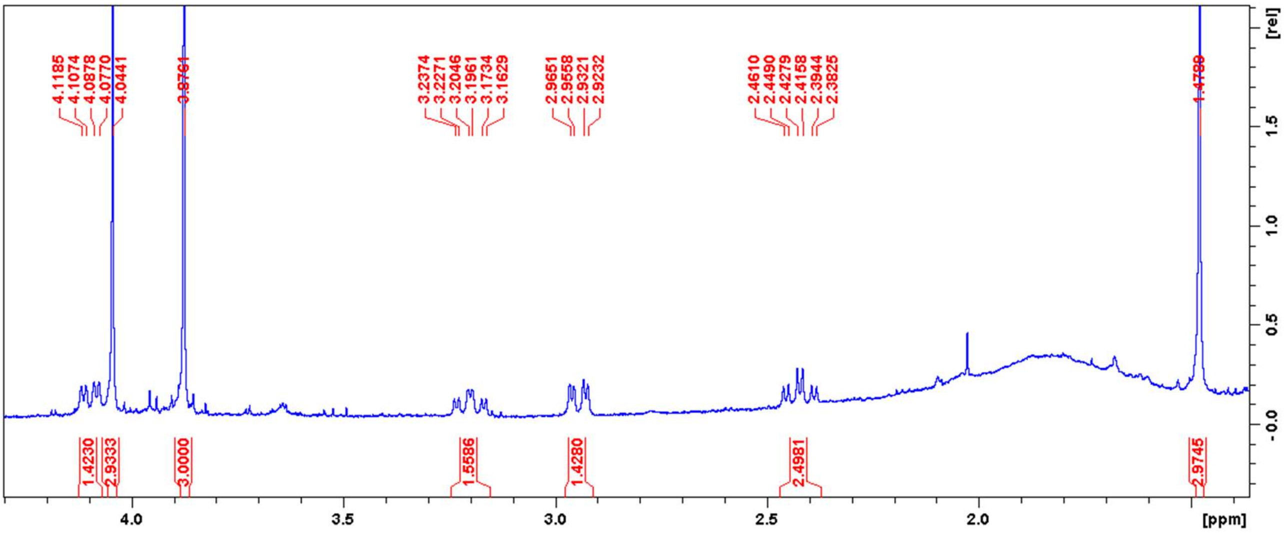

Figure S93. Expansion of the aliphatic hydrogen signals in the ^{1}H NMR spectrum of **guattouregidine** (**8**) in $CDCl_3$ at 500 MHz.

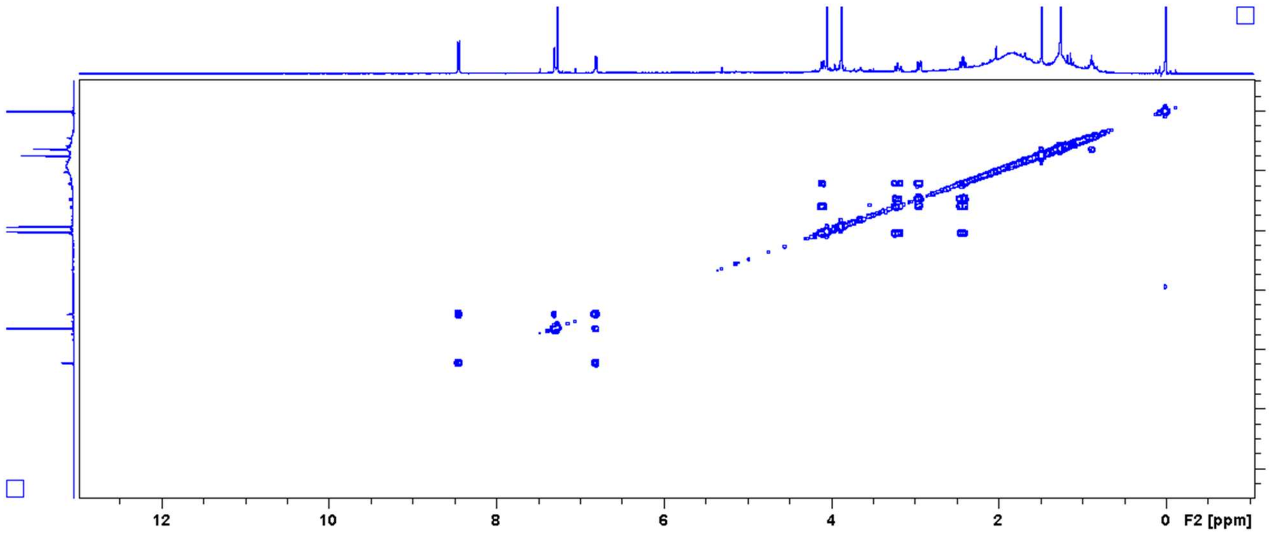

Figure S94. ^{1}H-^{1}H correlation map from the COSY NMR spectrum of **guattouregidine** (**8**) in $CDCl_3$ at 500 MHz.

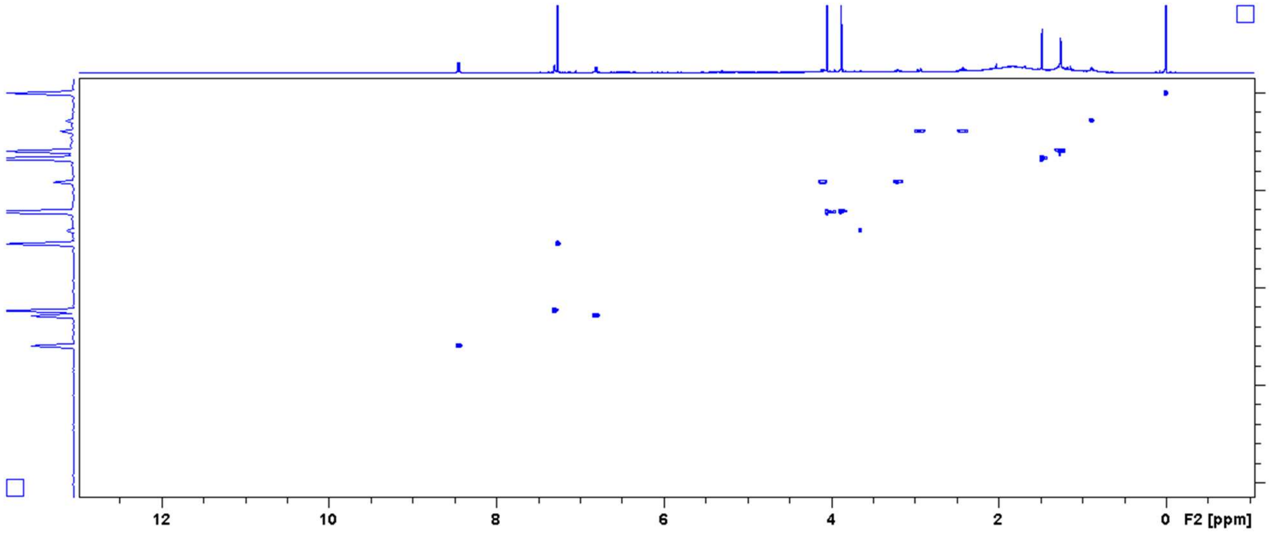

Figure S95. One-bond ^{1}H-^{13}C correlation map from the HSQC NMR spectrum of **guattouregidine** (**8**) in $CDCl_3$ at 500 (^{1}H) and 125 MHz (^{13}C).

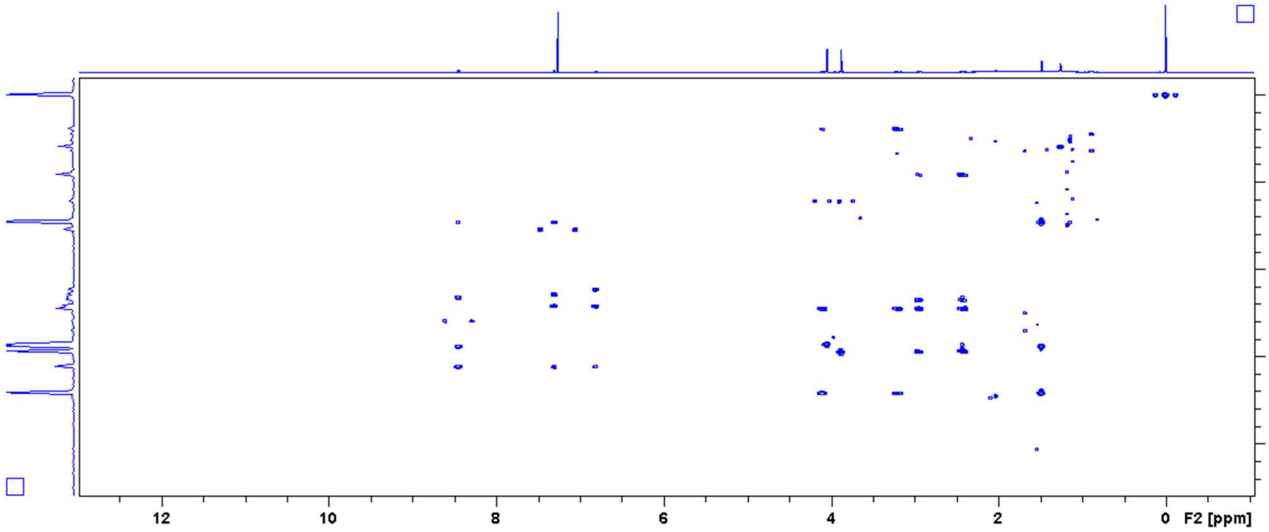

Figure S96. Long-range ^{1}H-^{13}C correlation map from the HMBC NMR spectrum of **guattouregidine** (**8**) in $CDCl_3$ at 500 (^{1}H) and 125 MHz (^{13}C).

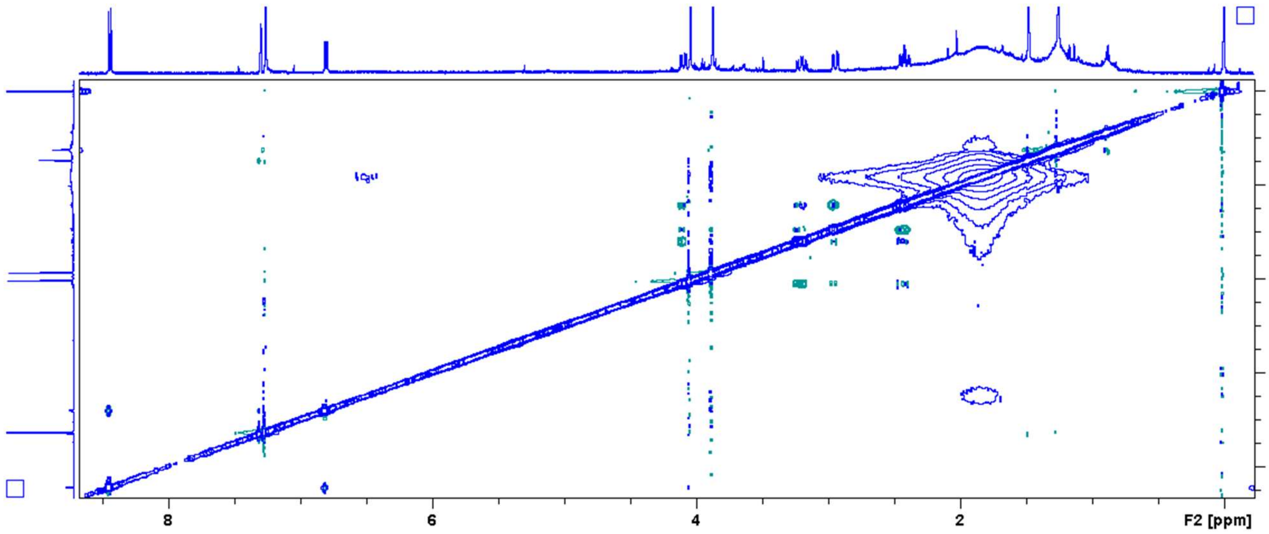

Figure S97. ^{1}H-^{1}H correlation map from NOESY NMR experiments of **guattouregidine** (**8**) in $CDCl_3$ at 500 (^{1}H) and 125 MHz (^{13}C).

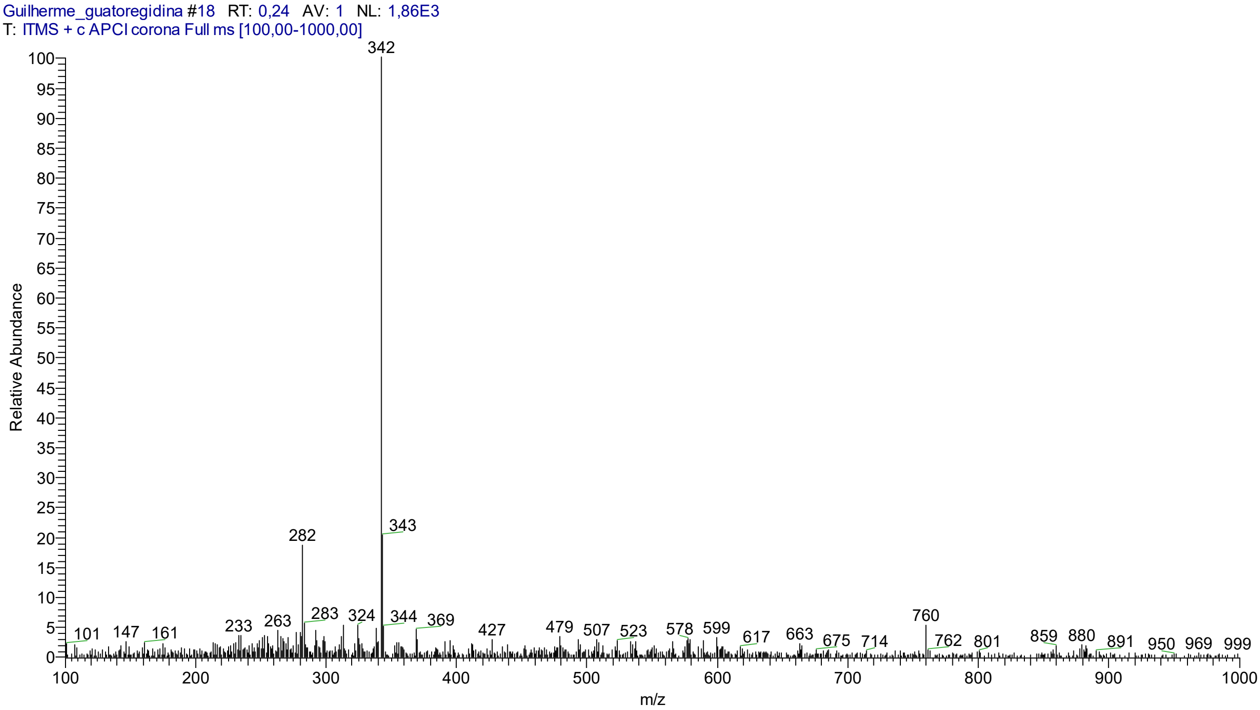

Figure S98. Full LR-APCI(+)-MS spectrum of **guattouregidine** (**8**) (m/z 342 $[M+H]^+$).

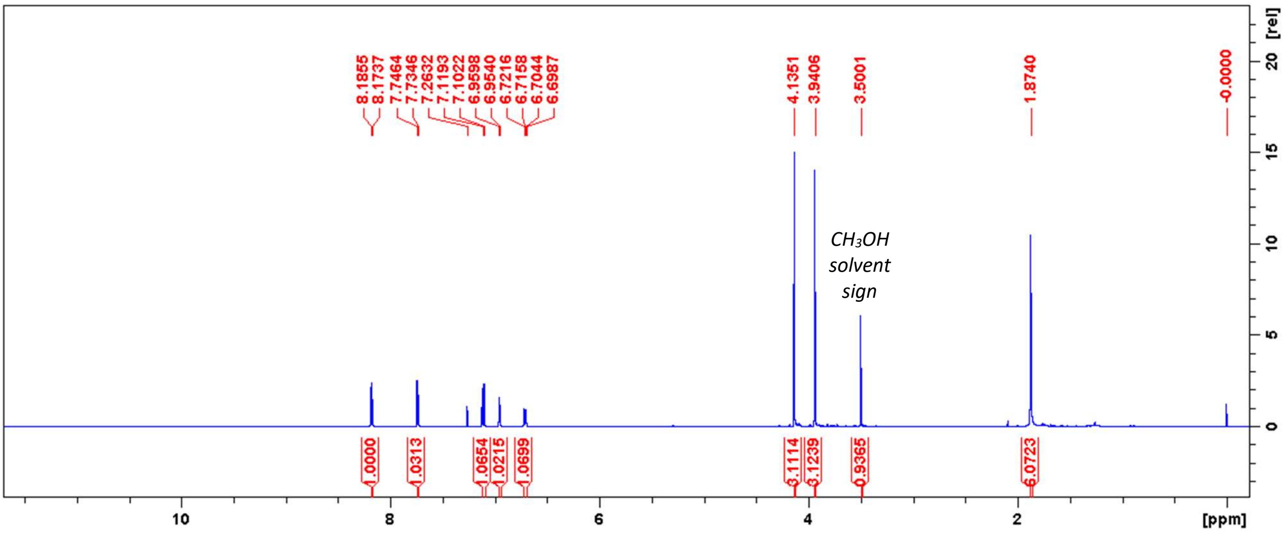

Figure S99. ^{1}H NMR spectrum of **gouregine** (**9**) in $CDCl_3$ at 500 MHz.

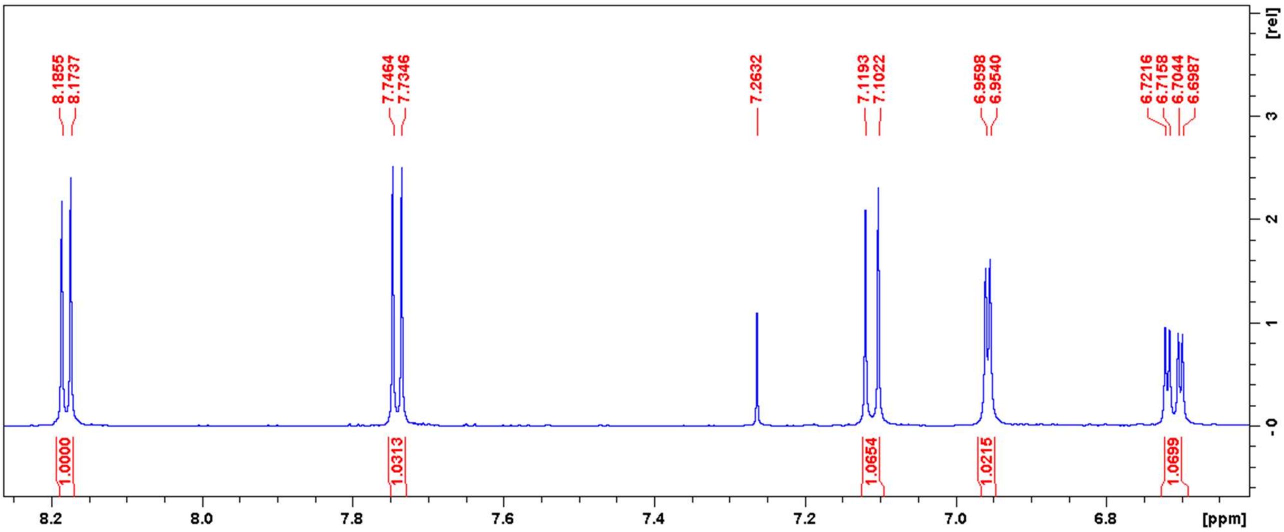

Figure S100. Expansion of aromatic hydrogen signals in the ^{1}H NMR spectrum of **gouregine** (**9**) in $CDCl_3$ at 500 MHz.

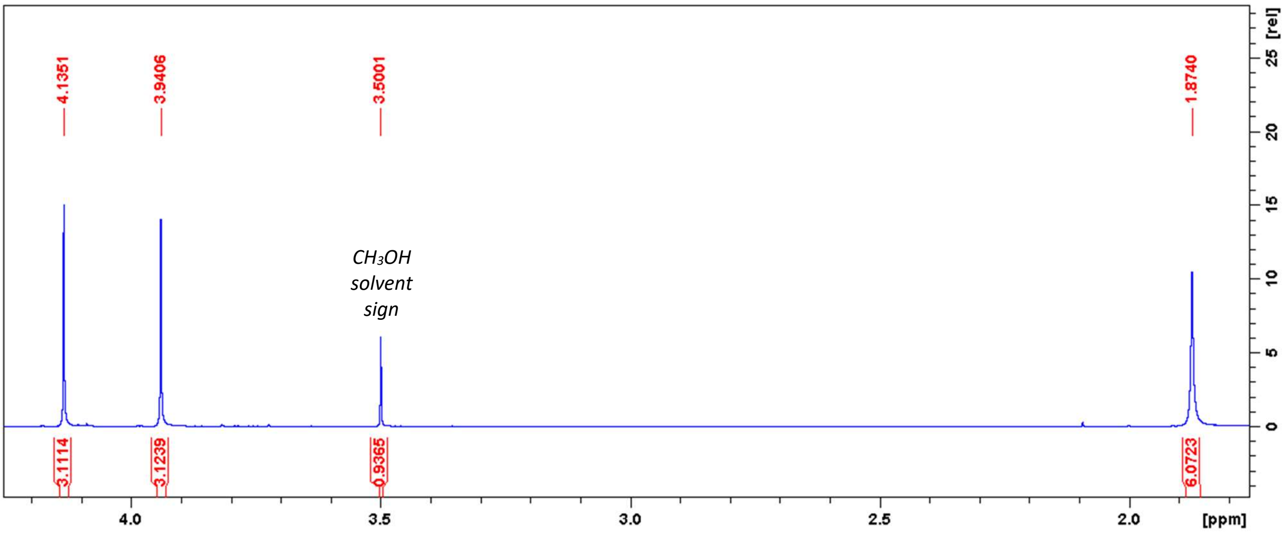

Figure S101. Expansion of the aliphatic hydrogen signals in the ^{1}H NMR spectrum of **gouregine** (**9**) in $CDCl_3$ at 500 MHz.

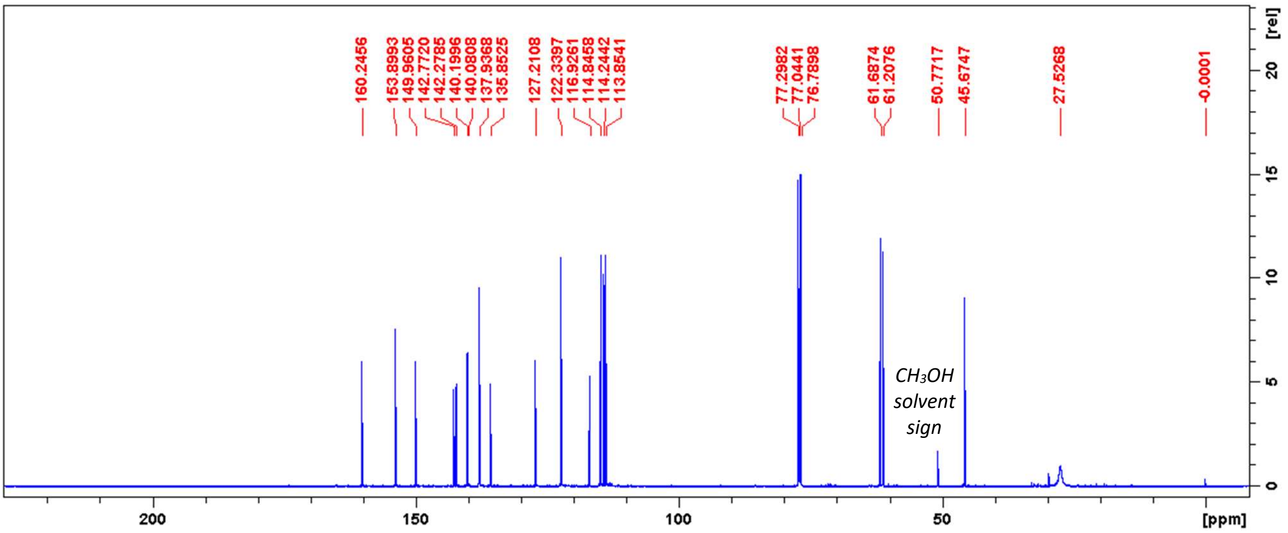

Figure S102. ^{13}C NMR spectrum of **gouregine** (**9**) in $CDCl_3$ at 125 MHz.

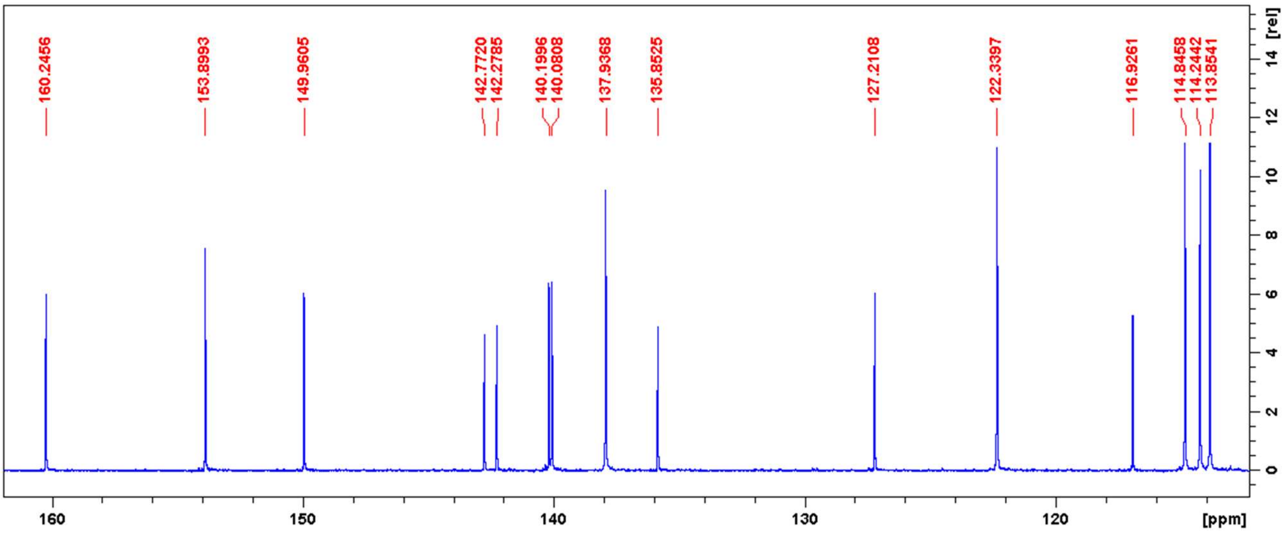

Figure S103. Expansion of aromatic carbon signals in the ^{13}C NMR spectrum of **gouregine** (**9**) in $CDCl_3$ at 125 MHz.

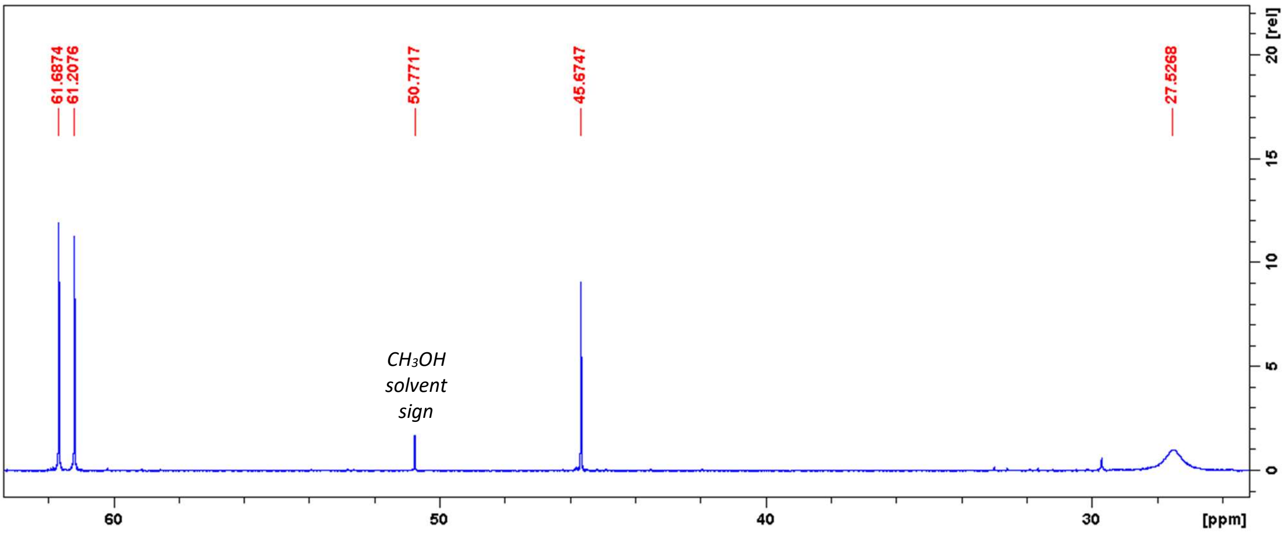

Figure S104. Expansion of the aliphatic carbon signals in the ^{13}C NMR spectrum of **gouregine 9** in $CDCl_3$ at 125 MHz.

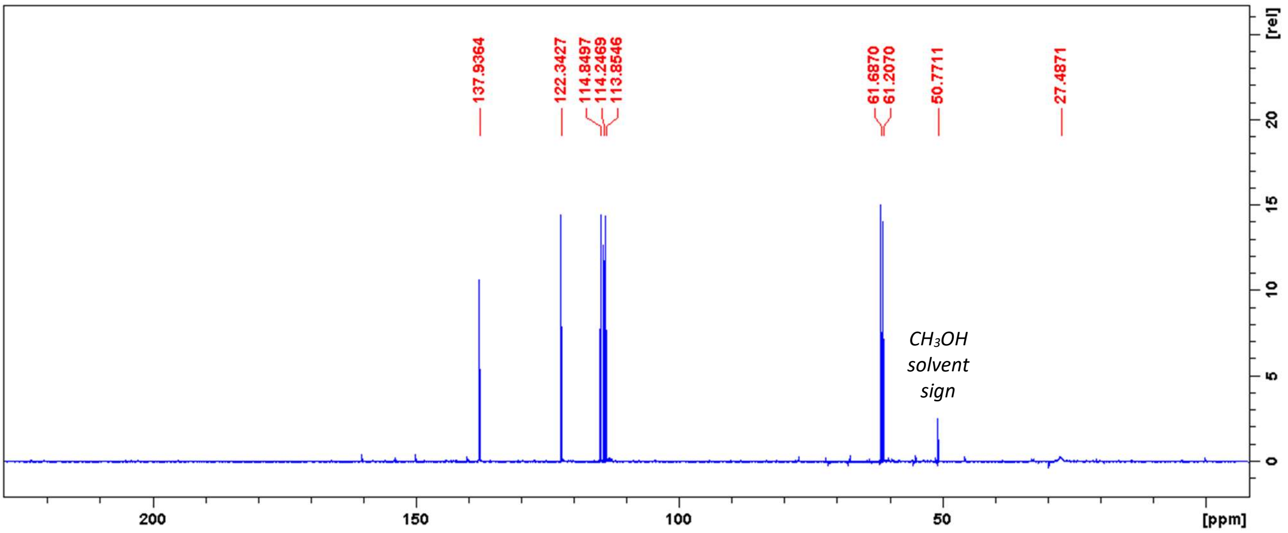

Figure S105. ^{13}C NMR DEPT 135 spectrum of **gouregine** (**9**) in $CDCl_3$ at 125 MHz.

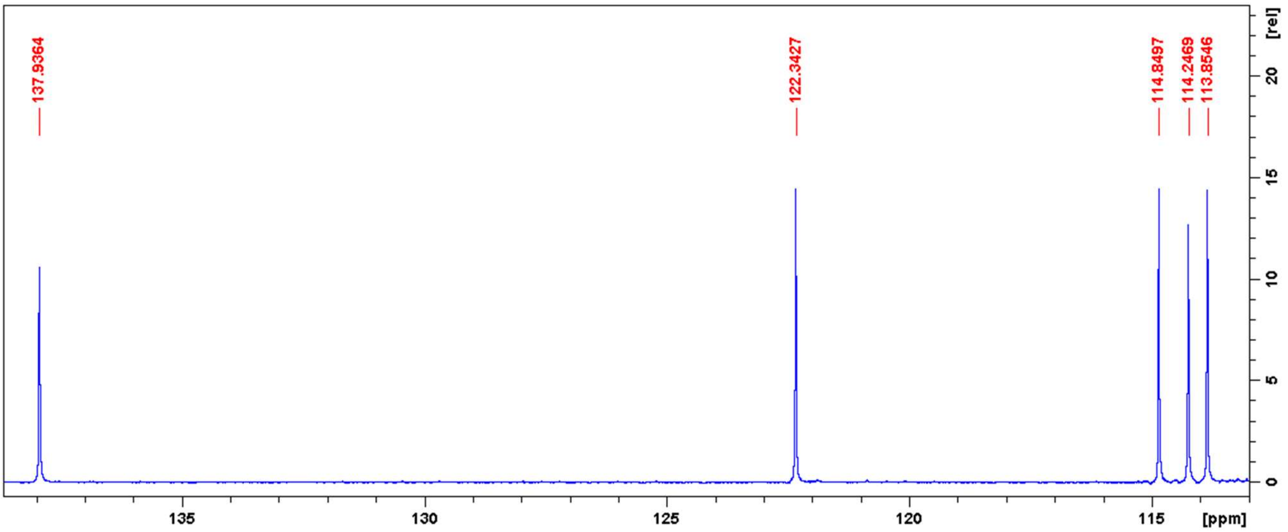

Figure S106. Expansion of aromatic carbon signals in the ^{13}C NMR DEPT 135 spectrum of **gouregine** (**9**) in $CDCl_3$ at 125 MHz.

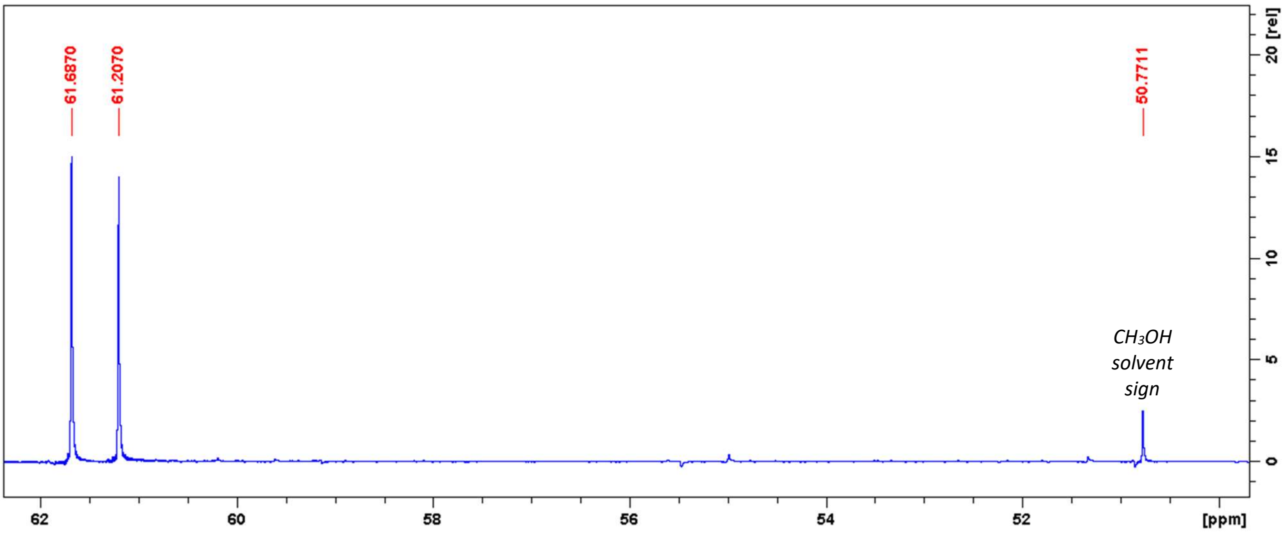

Figure S107. Expansion of the aliphatic carbon signals in the ^{13}C NMR DEPT 135 spectrum of **gouregine** (**9**) in $CDCl_3$ at 125 MHz.

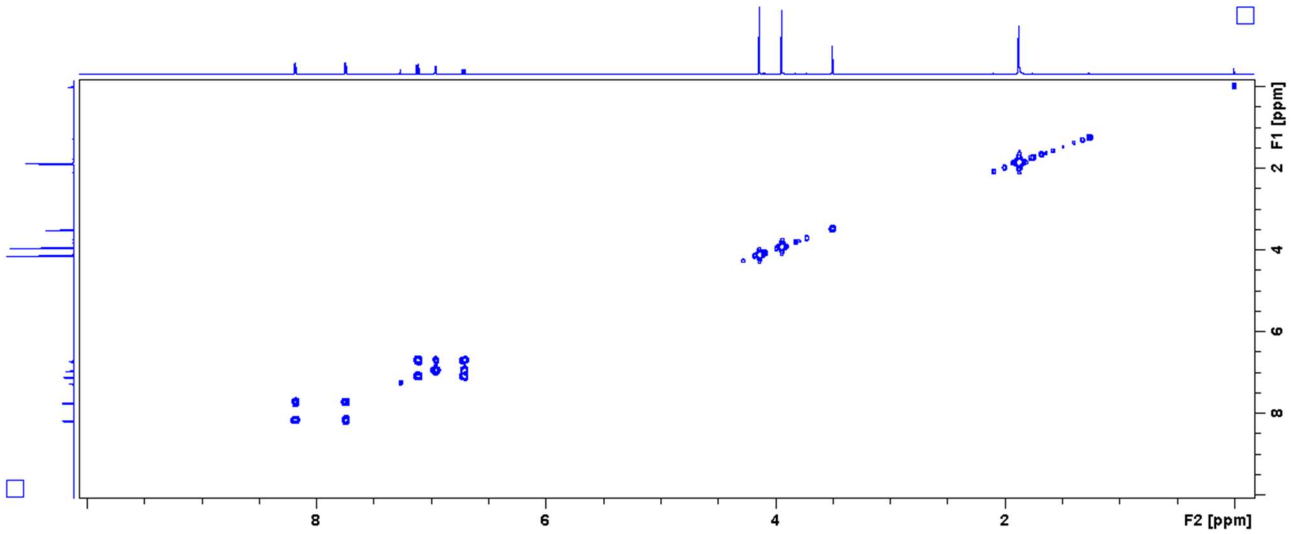

Figure S108. ^{1}H-^{1}H correlation map from the COSY NMR spectrum of **gouregine** (**9**) in $CDCl_3$ at 500 MHz.

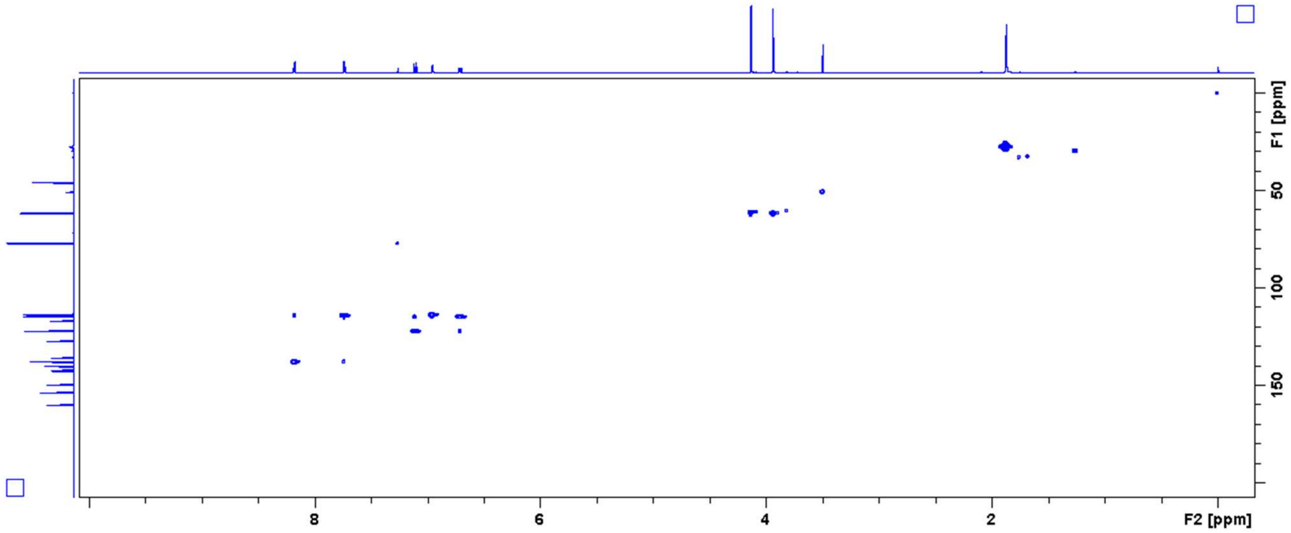

Figure S109. One-bond ^{1}H-^{13}C correlation map from the HSQC NMR spectrum of **gouregine** (**9**) in $CDCl_3$ at 500 (^{1}H) and 125 MHz (^{13}C).

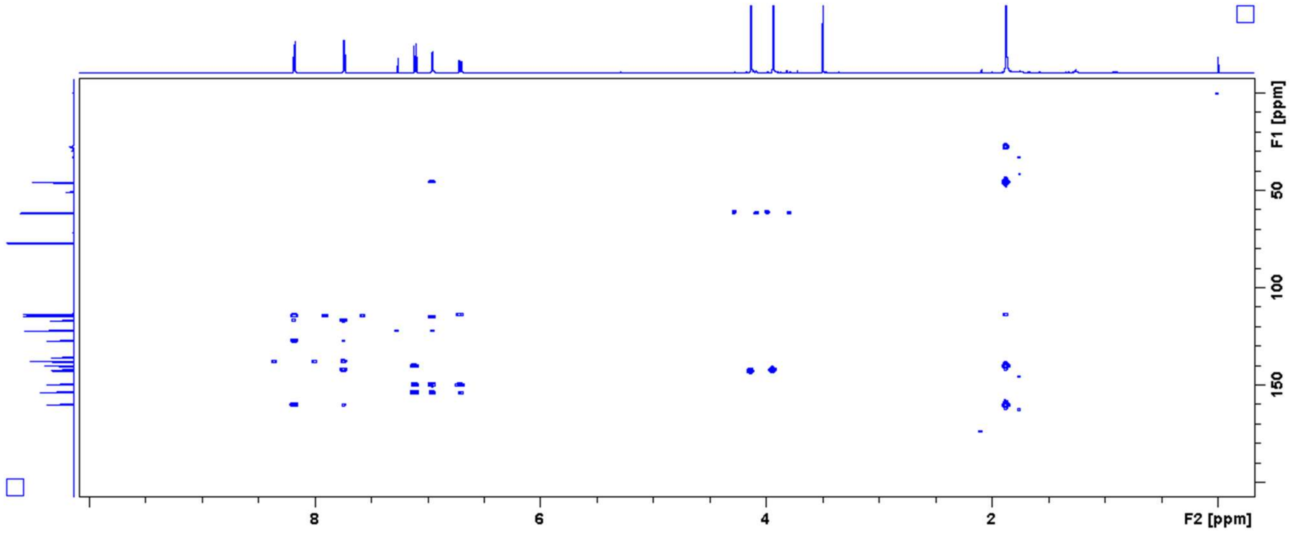

Figure S110. Long-range ^{1}H-^{13}C correlation map from the HMBC NMR spectrum of **gouregine** (**9**) in $CDCl_3$ at 500 (^{1}H) and 125 MHz (^{13}C).

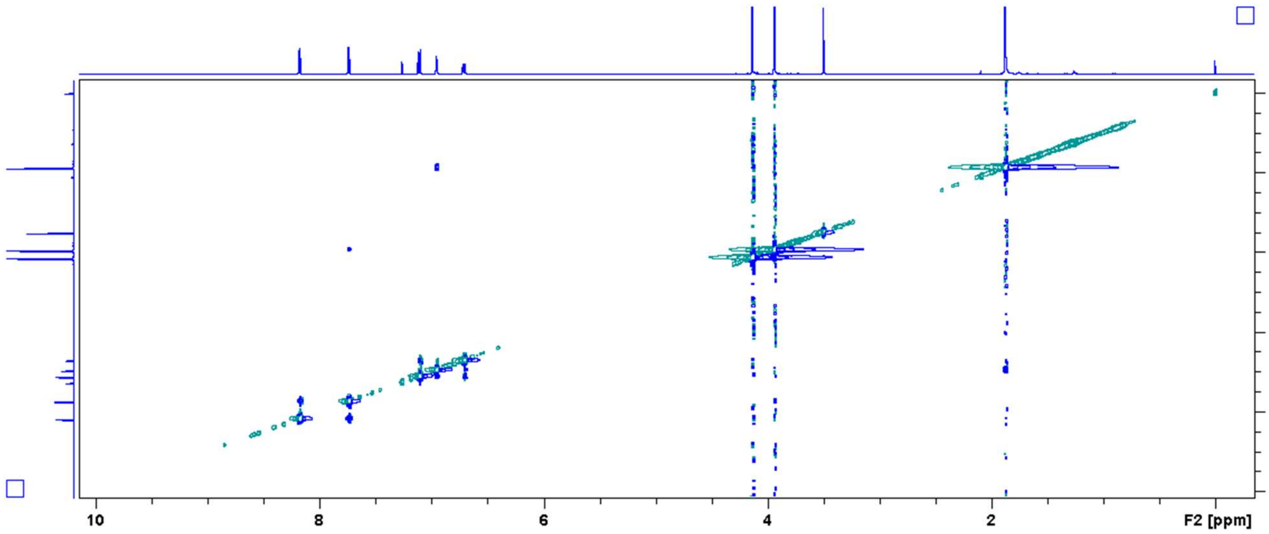

Figure S111. ^{1}H-^{1}H correlation map from NOESY NMR experiments of **gouregine** (**9**) in $CDCl_3$ at 500 (^{1}H) and 125 MHz (^{13}C).

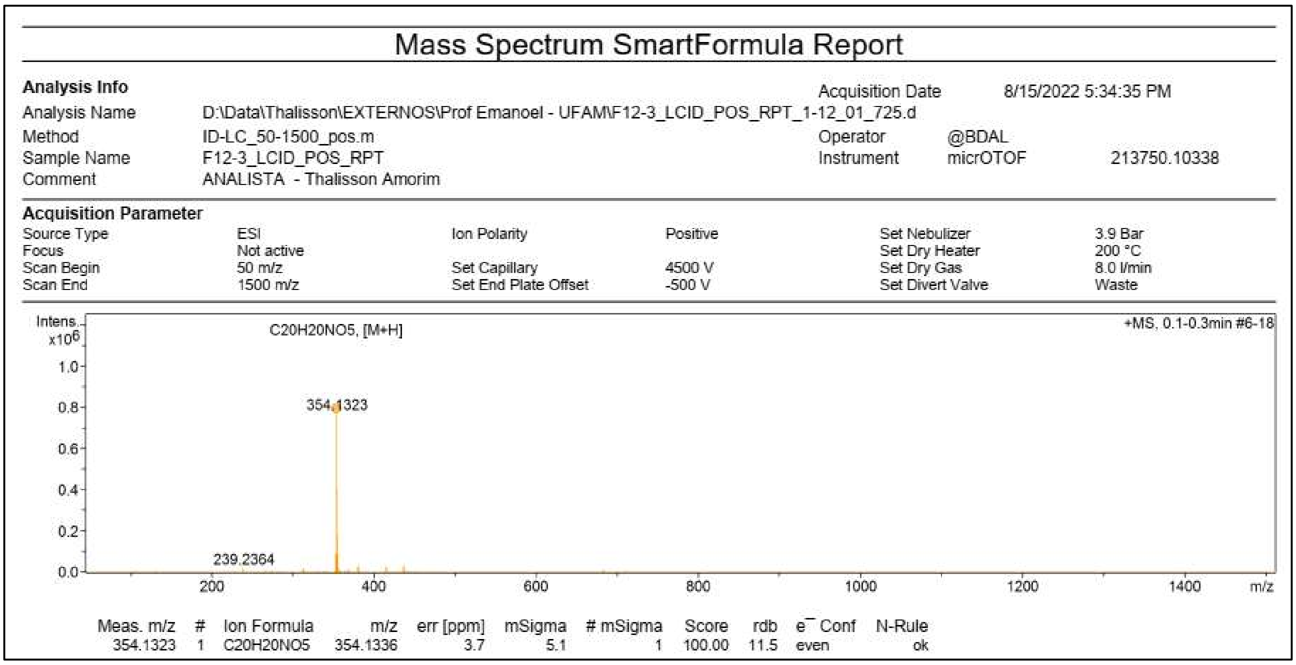

Figure S112. Full HRESIMS (+) spectrum of **gouregine** (**9**) (*m/z* 354.1323 [M+H]$^{+}$).

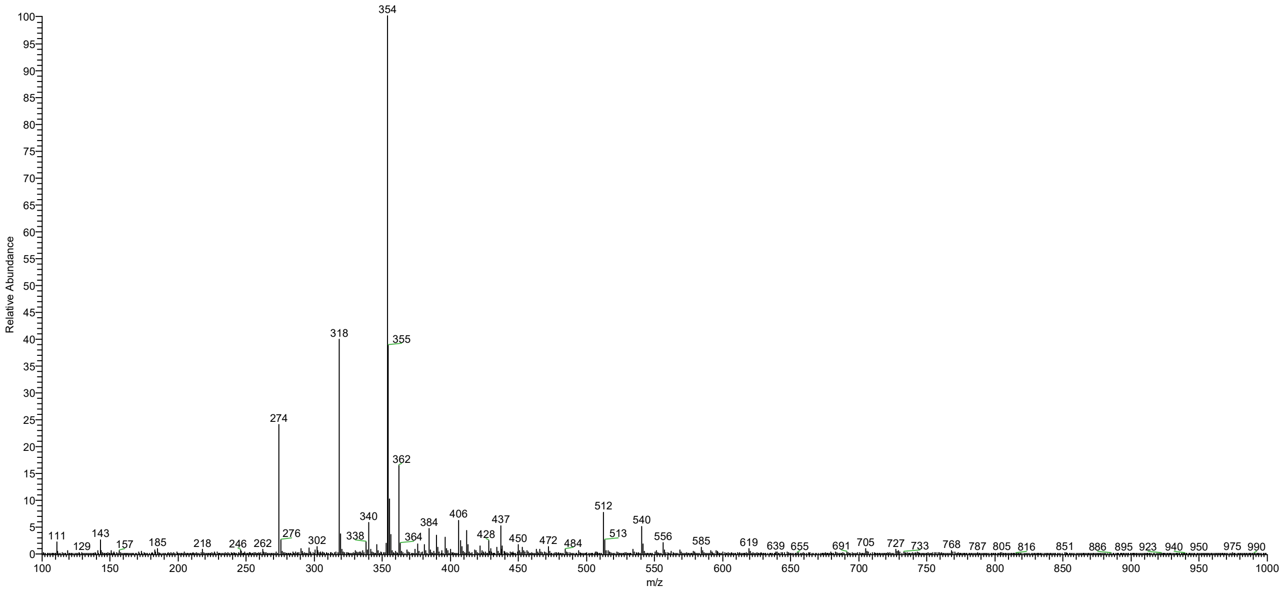

Figure S113. Full LR-APCI(+)-MS spectrum of **gouregine** (**9**) (*m/z* 340 [M+H]$^+$).

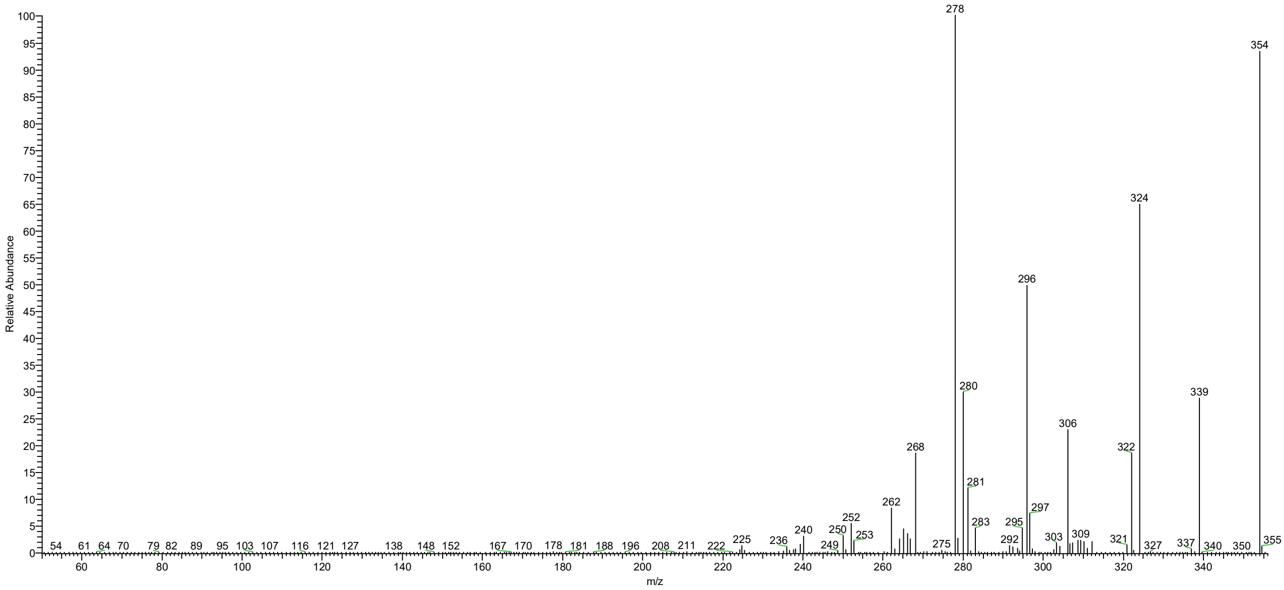

Figure S114. MS/MS fragmentation of the protonated molecule of **gouregine** (**9**) at *m/z* 354 [M+H]$^+$).

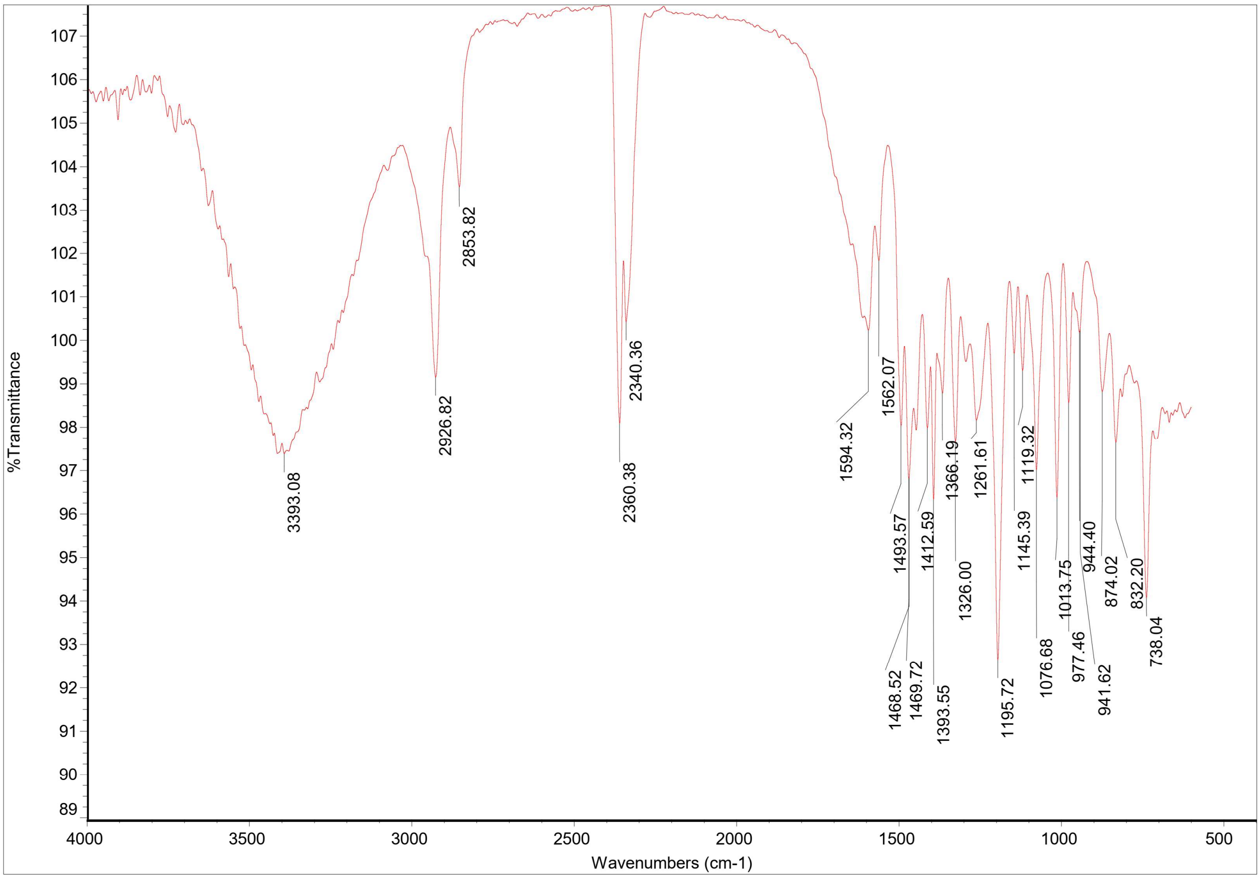

Figure S115. IR spectrum of **gouregine** (**9**) in KBr.

Table S1. Selectivity indices (SI) for compounds **1-9.**

**Compound**	**SI for noncancerous cells (MRC-5)**		
	**HepG2**	**KG-1a**	**HCT116**
Isopiline (**1**)	N.d.	N.d.	N.d.
O-Methylisopiline (**2**)	N.d.	N.d.	N.d.
Melosmine (**3**)	N.d.	N.d.	1.41
9-hydroxyiguattescine (**4**)	N.d.	N.d.	N.d.
Dihydromelosmine (**5**)	N.d.	N.d.	N.d.
Lysicamine (**6**)	N.d.	N.d.	2.60
Acanthoic acid (**7**)	N.d.	N.d.	N.d.
Guattouregidine (**8**)	N.d.	N.d.	N.d.
Gouregine (**9**)	N.d.	N.d.	N.d.
Doxorubicin	35.50	6.45	7.10

The data were calculated via the following formula: SI = IC_{50} [noncancerous cells]/IC_{50} [cancer cells]. N.d. = not determined.
